# Association of Healthy Lifestyle Factors and Obesity-Related Diseases in Adults in the UK

**DOI:** 10.1001/jamanetworkopen.2023.14741

**Published:** 2023-05-26

**Authors:** Nathalie Rassy, Alexis Van Straaten, Claire Carette, Mark Hamer, Claire Rives-Lange, Sébastien Czernichow

**Affiliations:** 1 Assistance Publique-Hôpitaux de Paris, Service de Nutrition, Centre Spécialisé Obésité, Hôpital Européen Georges Pompidou, Paris, France; 2 Assistance Publique-Hôpitaux de Paris, Service d’informatique Médicale, Biostatistiques Et Santé Publique, Hôpital Européen Georges Pompidou, Paris, France; 3 Université Paris Cité, Paris, France; 4 Division of Surgery Interventional Science, Institute of Sport Exercise and Health, University College London, United Kingdom; 5 INSERM, UMR1153, Methods Team, Epidemiology and Biostatistics Sorbonne Paris Cité Center, Paris, France; 6 Centre d’investigation clinique, INSERM 1418, Hôpital Européen Georges Pompidou, Assistance Publique-Hôpitaux de Paris, Paris, France

## Abstract

**Importance:**

A healthy lifestyle is associated with a reduced risk of cardiovascular disease in adults with obesity. Little is known about the associations between a healthy lifestyle and the risk of other obesity-attributable diseases in this population.

**Objective:**

To examine the association between healthy lifestyle factors and the incidence of major obesity-related diseases in adults with obesity compared with those with normal weight.

**Design, Setting, and Participants:**

This cohort study evaluated UK Biobank participants aged 40 to 73 years and free of major obesity-attributable disease at baseline. Participants were enrolled from 2006 to 2010 and prospectively followed up for disease diagnosis.

**Exposures:**

A healthy lifestyle score was constructed using information on not smoking, exercising regularly, no or moderate alcohol consumption, and eating a healthy diet. For each lifestyle factor, participants scored 1 if they met the criterion for a healthy lifestyle and 0 otherwise.

**Main Outcomes and Measures:**

The risk of outcomes according to the healthy lifestyle score in adults with obesity compared with those with normal weight were examined using multivariable Cox proportional hazards models with Bonferroni correction for multiple testing. The data analysis was performed between December 1, 2021, and October 31, 2022.

**Results:**

A total of 438 583 adult participants in the UK Biobank were evaluated (female, 55.1%; male, 44.9%; mean [SD] age, 56.5 [8.1] years), of whom 107 041 (24.4%) had obesity. During a mean (SD) follow-up of 12.8 (1.7) years, 150 454 participants (34.3%) developed at least 1 of the studied diseases. Compared with adults with obesity and 0 healthy lifestyle factors, individuals with obesity who met all 4 healthy lifestyle factors were at lower risk of hypertension (HR, 0.84; 95% CI, 0.78-0.90), ischemic heart disease (HR, 0.72; 95% CI, 0.65-0.80), arrhythmias (HR, 0.71; 95% 0.61-0.81), heart failure (HR, 0.65; 95% CI, 0.53-0.80), arteriosclerosis (HR, 0.19; 95% CI, 0.07-0.56), kidney failure (HR, 0.73; 95% CI, 0.63-0.85), gout (HR, 0.51; 95% CI, 0.38-0.69), sleep disorders (HR, 0.68; 95% CI, 0.56-0.83), and mood disorders (HR, 0.66; 95% CI, 0.56-0.78). The lifestyle profiles associated with the lowest risks included a healthy diet and at least 1 of the 2 healthy behaviors of physical activity and never smoking. Compared with adults with normal weight, those with obesity were at higher risk of several outcomes, irrespective of the lifestyle score (adjusted HRs ranged from 1.41 [95% CI, 1.27-1.56] for arrhythmias to 7.16 [95% CI, 6.36-8.05] for diabetes for adults with obesity and 4 healthy lifestyle factors).

**Conclusion and Relevance:**

In this large cohort study, adherence to a healthy lifestyle was associated with reduced risk of a wide range of obesity-related diseases, but this association was modest in adults with obesity. The findings suggest that although a healthy lifestyle seems to be beneficial, it does not entirely offset the health risks associated with obesity.

## Key Points

**Question:**

Is a healthy lifestyle more important for lowering the risk of obesity-related disease than obesity itself?

**Findings:**

In this cohort study of 438 583 UK Biobank participants, adults with obesity were at a higher risk of various diseases compared with those who maintained a healthy body weight and lifestyle. For individuals with obesity, engaging in regular exercise, refraining from smoking, consuming alcohol in moderation or not at all, and eating a healthy diet were associated with a decreased risk of cardiovascular disease, kidney failure, gout, sleep disorders, and mood disorders.

**Meaning:**

These findings suggest that although adherence to a healthy lifestyle is associated with a reduced risk of several adverse health outcomes in adults with obesity, it does not entirely eradicate the risk of obesity-related diseases.

## Teaser

This cohort study uses UK Biobank data to examine the association of adhering to healthy lifestyle factors with the incidence of obesity-related diseases.

## Introduction

Obesity is the most prevalent chronic disease worldwide. It is estimated that more than 1 billion people will be living with obesity by 2030.^1^ The higher rates of obesity-associated mortality and comorbidities, such as diabetes, cardiovascular disease, chronic kidney disease, and several types of cancer, are equally staggering, with an average of 5 million deaths and 160 million disability-adjusted life-years.^1^ As a result, trends toward reduced productivity and greater intensity of health care service use are expected to result in a major economic burden.^2,3^ A need exists for the treatment of obesity as a chronic, progressive, and relapsing disease to improve outcomes for people living with this disease. A suboptimal lifestyle is a major preventable cause of obesity and its associated comorbidities.^4^ As such, interventions to improve lifestyle are an opportunity to optimize the management of obesity.

Several healthy lifestyle factors, such as being physically active, avoiding smoking, and adhering to healthy diet patterns, both individually and jointly, are associated with lower disease and mortality rates in the general population.^5,6,7,8,9,10^ Despite nutritional guidelines, less is known about such correlations across the body mass index (BMI) spectrum, particularly in adults with obesity. The few existing investigations showed that individual healthy lifestyle factors, such as smoking avoidance, healthy dietary patterns (eg, adherence to the Mediterranean diet), or more leisure-time physical activity, may reduce the risk of chronic disease and mortality associated with obesity.^11,12,13,14^

Behavioral factors are often mutually linked, and people tend to follow interrelated lifestyle patterns.^15^ Lifestyle factors should therefore be analyzed jointly to better evaluate their health impact. It has been shown that meeting more than 1 healthy lifestyle factor reduces the risk of cardiovascular disease, cardiovascular mortality, and all-cause mortality in adults with overweight and obesity.^16,17,18,19^ It remains unclear to what extent specific combinations of healthy lifestyle factors are associated with a reduced risk of obesity-related diseases other than cardiovascular disease. Furthermore, whether a healthy lifestyle offsets the risk of obesity remains to be elucidated. The aim of this study is to estimate the association between healthy lifestyle factors, such as physical activity, never smoking, no or moderate alcohol consumption, and a healthy diet, and the incidence of major obesity-related diseases in adults with obesity compared with those with a normal weight.

## Methods

### Study Population

Data for this cohort study were obtained from the UK Biobank. The UK Biobank is a large prospective cohort study that recruited more than 500 000 volunteers aged 40 and 69 years in 2006-2010. Details of the UK Biobank cohort are described elsewhere.^20^ For this study, demographic data were collected on age, sex, socioeconomic status (Townsend deprivation index), and lifestyle factors. In the UK Biobank cohort study, participants self-identified their ethnicity via a touchscreen questionnaire, but we did not collect or analyze data on race and ethnicity for this study. The North West Multi-centre Research Ethics Committee approved the collection and use of UK Biobank data in accordance with the principles of the Declaration of Helsinki. The UK Biobank study was approved by the North West Multi-centre Research Ethics Committee, and written informed consent was already provided by each participant. This research was conducted using the UK Biobank Resource under application number 23476, and the study followed the Strengthening the Reporting of Observational Studies in Epidemiology (STROBE) reporting guideline.

### Assessment of Lifestyle Factors

Four lifestyle factors were investigated, including smoking, alcohol consumption, physical activity, and diet. Data about these lifestyle factors were obtained from touchscreen baseline questionnaires at the UK Biobank assessment center. The codes used to define the variables included in this study are reported in eTable 1 in Supplement 1. For tobacco smoking, participants were grouped into never, former, and current smokers based on their responses to the questions, “Do you smoke tobacco now?” and “In the past, how often have you smoked tobacco?” For alcohol consumption, the number of drinks per week was computed by summing responses to the average weekly consumption of champagne, white wine, beer, cider, spirits, fortified wine, and other types of alcohol consumption. For physical activity, the number of days per week and duration of vigorous physical activity, moderate physical activity, and walking were estimated via validated short International Physical Activity Questionnaire.^21^ For dietary assessment, participants were asked to complete a validated questionnaire that included questions on food consumption frequency over the past year and the avoidance of some foods.^22^

For each lifestyle factor, participants were assigned a score of 1 if they met the criterion for a healthy lifestyle and 0 otherwise. For smoking status, a score of 1 was assigned to never smoker and 0 to current or former smoker. For alcohol consumption, a score of 1 was assigned to nonheavy drinking (men, <10 drinks per week; women, <5 drinks per week) and 0 otherwise (1 drink contains 8 g of ethanol in the UK). For physical activity, a score of 1 was assigned to those who met the World Health Organization recommendations (≥2.5 hours of moderate-intensity activity per week, ≥1.25 hours of vigorous-intensity activity per week, or an equivalent combination of moderate- and vigorous-intensity activity).^23^ For diet, a score of 1 was assigned to those who met at least 5 of the dietary recommendations for cardiovascular health^24^ (increased consumption of fruits, vegetables, whole grains, fish or shellfish, dairy products, and vegetable oils and reduced or no consumption of refined grains, processed and unprocessed meats, and sugar-sweetened beverages) and 0 otherwise. The definitions and variables used for dietary components were based on 2 previous UK Biobank studies.^25,26^ The healthy lifestyle score was computed by summing the 4 scores. It ranged between 0 (lowest healthy score) and 4 (highest healthy score). Furthermore, lifestyle profiles were created based on the number of healthy lifestyle factors, as described in Nyberg et al^9^: 1 profile with any healthy lifestyle factor, 4 profiles with 1 healthy lifestyle factor, 6 profiles with 2 healthy lifestyle factors, 4 profiles with 3 healthy lifestyle factors, and 1 profile with 4 healthy lifestyle factors. The definition and scoring of lifestyle factors are reported in eTable 2 in Supplement 1.

### Ascertainment of Outcomes

The outcomes of interest were classified using the *International Statistical Classification of Diseases, Tenth Revision* (*ICD-10*). The outcomes included the incidence of the major diseases correlated with obesity^27,28^: infections (bacterial and viral), diabetes, cancer (esophageal adenocarcinoma, gastric cardia, pancreatic, liver, kidney, colorectal, ovarian [in women], endometrial [in women], and breast cancer [in women]), cardiovascular disease (hypertension, ischemic heart disease, pulmonary embolism, arrhythmias, heart failure, cerebrovascular diseases, arteriosclerosis, and deep vein thrombosis), asthma, liver disease, kidney failure, gout, osteoarthritis, sleep disorders, and mood disorders. The diagnosis of the diseases during follow-up was obtained from primary and secondary hospital inpatient records and reported causes of death.^29^ The *ICD-10* codes used to define diseases are reported in eTable 3 in Supplement 1.

### Statistical Analysis

The data analysis was performed between December 1, 2021, and October 31, 2022. Data were reported as mean (SD) or number (percentage). Baseline characteristics were described across different levels of BMI (as measured by weight in kilograms divided by height in meters squared). Differences across BMI classes were tested by 2-tailed analysis of variance and Pearson χ^2^ test. After examining the proportional hazards assumption, Cox proportional hazards regression models were used to estimate the hazard ratios (HRs) and 95% CIs of incident diseases according to the BMI group and stratified by the overall healthy lifestyle score using a BMI of 18.5 to 24.9 as the reference group. Follow-up started from baseline at study entry until the onset of the diseases of interest, loss to follow-up, death, or end of follow-up, whichever came first. All data were censored on September 30, 2021, for England; July 31, 2021, for Scotland; and February 28, 2018, for Wales. A multivariable-adjusted model was constructed to account for age, sex, and Townsend deprivation index. A further analysis was conducted to estimate the associations of healthy lifestyle scores with incident diseases by BMI category using the lowest healthy lifestyle score as the reference category. We repeated the main analyses with an alternative outcome that included all-cause mortality. Date of death was obtained from death certificates held within the National Health Service Information Centre for England and Wales and the National Health Service Central Register Scotland for Scotland. In sensitivity analysis, we repeated all analyses after the exclusion of participants with diseases diagnosed within the first 2 years of follow-up to minimize the effect of reverse causation. Bonferroni correction was used to account for multiple testing in the cause-specific analysis and stratified analysis. After Bonferroni correction, all *P* values were adjusted and compared with the significance level α of .05. Data were analyzed using R, version 4.1.3 statistical software (R Foundation for Statistical Computing).

## Results

Of the 502 616 participants in the UK Biobank, 5820 with a BMI less than 18; 40 146 extreme outliers; and 16 340 with missing information on age, sex, socioeconomic status, and lifestyle factors were excluded from the analysis. Furthermore, 1727 participants with a record of any of the studied diseases at baseline were excluded. The final analytic sample included 438 583 participants (eFigure 1 in Supplement 1).

The baseline characteristics of participants in the UK Biobank are presented in Table 1. The mean (SD) age at baseline was 56.5 (8.1) years; 241 790 participants were women (55.1% vs 196 793 men [44.9%]), 107 041 (24.4%) had obesity, and the mean (SD) Townsend deprivation index was −1.38 (3.05). A total of 242 133 participants (55.2%) were never smokers, 256 002 (58.4%) were nonheavy alcohol drinkers, 301 397 (68.7%) were physically active, and 276 957 (63.2%) reported eating a healthy diet as defined in the study. The proportion of participants with healthy lifestyle scores of 0, 1, 2, 3, and 4 was 3.1% (n = 13 428), 14.9% (n = 65 594), 32.0% (n = 140 453), 33.4% (n = 146 443), and 16.6% (n = 72 665), respectively.

**Table 1.** Baseline Characteristics of Participants From the UK Biobank According to BMI

Characteristic	No. (%)				*P* value
	Total population (N = 438 583)	BMI 18.5-24.9 (n = 144 579)	BMI 25.0-29.9 (n = 186 963)	BMI ≥30.0 (n = 107 041)	
Age, mean (SD), y	56.5 (8.1)	55.6 (8.2)	56.9 (8.1)	56.8 (7.9)	<.001
Sex					
Female	241 790 (55.1)	95 283 (65.9)	89 480 (47.9)	57 027 (53.3)	<.001
Male	196 793 (44.9)	49 296 (34.1)	97 483 (54.1)	50 014 (46.7)	
Townsend deprivation index, mean (SD)^a^	−1.38 (3.1)	−1.56 (2.9)	−1.51 (2.9)	−0.91 (3.2)	<.001
BMI, mean (SD)	27.5 (4.8)	22.9 (1.5)	27.3 (1.4)	33.9 (3.9)	<.001
Healthy lifestyle factors					
Never smoking	242 133 (55.2)	86 388 (59.8)	100 936 (53.9)	54 807 (51.2)	<.001
No or moderate alcohol consumption^b^	256 002 (58.4)	82 232 (56.9)	106 293 (56.9)	67 477 (63.1)	<.001
Physical activity^c^	301 397 (68.7)	106 948 (73.8)	130 450 (69.8)	63 999 (59.8)	<.001
Healthy diet^d^	276 957 (63.2)	97 720 (67.6)	116 562 (62.3)	62 675 (58.6)	<.001
Healthy lifestyle score^e^					<.001
0	13 428 (3.1)	3190 (2.2)	5857 (3.1)	4381 (4.1)	
1	65 594 (14.9)	17 689 (12.2)	28 851 (15.4)	19 054 (17.8)	
2	140 453 (32.1)	44 001 (30.4)	60 935 (32.6)	35 517 (33.2)	
3	146 443 (33.4)	51 199 (35.4)	61 760 (33.1)	33 484 (31.3)	
4	72 665 (16.6)	28 500 (19.7)	29 560 (15.8)	14 605 (13.6)	
No. of cases of diseases					
Infections	17 932 (4.1)	5051 (3.5)	7312 (3.9)	5569 (5.2)	<.001
Diabetes	20 411 (4.7)	2061 (1.4)	6860 (3.7)	11 500 (10.7)	<.001
Esophageal adenocarcinoma	726 (0.2)	164 (0.1)	329 (0.2)	233 (0.2)	<.001
Gastric cardia cancer	307 (0.1)	60 (0.04)	132 (0.1)	115 (0.1)	<.001
Pancreatic cancer	790 (0.2)	219 (0.2)	345 (0.2)	226 (0.2)	<.001
Liver cancer	451 (0.1)	114 (0.1)	172 (0.1)	165 (0.2)	<.001
Colorectal cancer	3793 (0.9)	1008 (0.7)	1758 (0.1)	1027 (1.0)	<.001
Breast cancer	6759 (1.5)	2436 (1.7)	2566 (1.4)	1757 (1.6)	<.001
Ovarian cancer	763 (0.2)	265 (0.2)	303 (0.2)	195 (0.2)	.26
Endometrial cancer	962 (0.2)	216 (0.2)	320 (0.2)	426 (0.4)	<.001
Kidney cancer	933 (0.2)	178 (0.1)	426 (0.2)	329 (0.3)	<.001
Hypertension	66 302 (15.1)	12 778 (8.8)	28 693 (15.4)	24 831 (23.2)	<.001
Ischemic heart disease	28 625 (6.5)	5521 (3.8)	12 615 (6.8)	10 489 (9.8)	<.001
Pulmonary embolism	3258 (0.7)	654 (0.5)	1382 (0.7)	1222 (1.1)	<.001
Arrhythmias	17 997 (4.1)	4257 (2.9)	7481 (4.0)	6259 (5.9)	<.001
Heart failure	6137 (1.4)	1136 (0.8)	2308 (1.2)	2693 (2.5)	<.001
Cerebrovascular diseases	8001 (1.8)	2037 (1.4)	3432 (1.8)	2532 (2.4)	<.001
Arteriosclerosis	456 (0.1)	110 (0.1)	198 (0.1)	148 (0.2)	<.001
Deep vein thrombosis	3164 (0.7)	731 (0.5)	1303 (0.7)	1130 (1.1)	<.001
Asthma	19 414 (4.4)	5082 (3.5)	7914 (4.2)	6418 (6.0)	<.001
Liver disease	5513 (1.3)	1094 (0.8)	2114 (1.1)	2305 (2.2)	<.001
Kidney failure	13 027 (2.9)	2485 (1.7)	5147 (2.8)	5395 (5.0)	<.001
Gout	2449 (0.6)	213 (0.2)	990 (0.5)	1246 (1.2)	<.001
Osteoarthritis	36 665 (8.4)	7112 (4.9)	14 853 (7.9)	14 700 (13.7)	<.001
Sleep disorders	4471 (1.0)	497 (0.3)	1294 (0.7)	2698 (2.5)	<.001
Mood disorders	13 375 (3.1)	3413 (2.4)	5116 (2.7)	4846 (4.5)	<.001

Abbreviation: BMI, body mass index (calculated as weight in kilograms divided by height in meters squared).

^a^ Higher scores indicate more deprivation.

^b^ Moderate alcohol consumption was considered an intake of fewer than 10 drinks per week for men and fewer than 5 drinks per week for women.

^c^ Moderate intensity physical activity was considered to be greater than or equal to 2.5 hours per week, and vigorous intensity activity was considered to be greater than or equal to 1.25 hours per week or an equivalent combination.

^d^ A healthy diet was considered an adequate intake of 5 or more healthy dietary components for cardiovascular health.

^e^ The healthy lifestyle score included 4 lifestyle factors (never smoking, no or moderate alcohol consumption, physical activity, and healthy diet). Participants scored 1 if they met the criterion for a healthy lifestyle and 0 otherwise.

During a mean (SD) follow-up of 12.8 (1.7) years, 150 454 participants (34.3%) developed at least 1 incident disease. Participants with obesity had a higher incidence of all studied diseases except ovarian cancer after adjustment for age, sex, Townsend deprivation index, and healthy lifestyle score (adjusted HRs varied between 1.17 [95% CI, 1.10-1.24] and 6.66 [95% CI, 6.36-6.98], with significant *P* values after Bonferroni correction for multiple testing) (eFigures 2 and 3 in Supplement 1).

eFigures 11 and 12 in Supplement 1 show the association between lifestyle score and the incidence of diseases in adults with normal weight and obesity after adjustment for age, sex, and Townsend deprivation index. In adults with obesity, the healthiest lifestyle score was associated with a lower risk of hypertension (HR, 0.84; 95% CI, 0.78-0.90), ischemic heart disease (HR, 0.72; 95% CI, 0.65-0.80), arrhythmias (HR, 0.71; 95% CI, 0.61-0.81), heart failure (HR, 0.65; 95% CI, 0.53-0.80), arteriosclerosis (HR, 0.19; 95% CI, 0.07-0.56), kidney failure (HR, 0.73; 95% CI, 0.63-0.85), gout (HR, 0.51; 95% CI, 0.38-0.69), sleep disorders (HR, 0.68; 95% CI, 0.56-0.83), and mood disorders (HR, 0.66; 95% CI, 0.56-0.78), with significant *P* values after Bonferroni correction for multiple testing. In the absence of adjustments (eFigure 4 in Supplement 1), the HRs were comparatively lower. Before adjustment, the healthiest lifestyle score was associated with a lower risk of hypertension (HR, 0.80; 95% CI, 0.74-0.85), ischemic heart disease (HR, 0.55; 95% CI, 0.49-0.61), arrhythmias (HR, 0.58; 95% CI, 0.51-0.67), heart failure (HR, 0.50; 95% CI, 0.41-0.62), arteriosclerosis (HR, 0.14; 95% CI, 0.05-0.39), kidney failure (HR, 0.67; 95% CI, 0.57-0.77), gout (HR, 0.31; 95% CI, 0.23-0.42), sleep disorders (HR, 0.53; 95% CI, 0.43-0.65), and mood disorders (HR, 0.77; 95% CI, 0.66-0.90). The HRs of diseases for adults with overweight with 1 or more healthy lifestyle factors compared with those without any healthy lifestyle factors are shown in eFigures 4 and 5 in Supplement 1. The healthiest lifestyle score was associated with a lower risk of all-cause mortality in adults with normal weight (HR, 0.32; 95% CI, 0.27-0.39), overweight (HR, 0.45; 95% CI, 0.39-0.52), and obesity (HR, 0.57; 95% CI, 0.48-0.68) after adjustment for age, sex, and Townsend deprivation index (eFigure 6 in Supplement 1).

Table 2 provides the adjusted HRs (95% CIs) for obesity-related diseases according to 16 lifestyle profiles in the overall study population and in each BMI category. In the overall population, the lifestyle profiles that were associated with the lowest HRs included a healthy diet and at least 2 healthy behaviors of physical activity and never smoking, with adjusted HRs less than 0.80 (0.79 [95% CI, 0.76-0.82], 0.70 [95% CI, 0.64-0.76], and 0.76 [95% CI, 0.71-0.81] in the overall, normal weight, and overweight cohorts, respectively). In adults with obesity, the HR was 0.87 (95% CI, 0.82-0.93) for the profile that included a healthy diet and never smoking, 0.84 (95% CI, 0.78-0.89) for the profile that included a healthy diet and physical activity, and 0.80 (95% CI, 0.75-0.85) for the profile that included a healthy diet, never smoking, and physical activity. The unadjusted HRs for incident diseases by lifestyle profile in each BMI category were comparatively lower (eTable 4 in Supplement 1). In individuals with obesity, the HRs before adjustment were 0.80 (95% CI, 0.75-0.86) for the profile that included a healthy diet and never smoking and 0.74 (95% CI, 0.69-0.79) for the profile that included a healthy diet, physical activity, and never smoking. The HRs for all-cause mortality according to the lifestyle profiles are presented in eTable 5 in Supplement 1. In adults with obesity, all the studied lifestyle profiles were associated with a reduced risk of all-cause mortality, except for the profiles that included never smoking and no or moderate alcohol consumption alone or combined (eTables 5 and 6 in Supplement 1).

**Table 2.** Cox Proportional Hazards Models for Incident Diseases by Lifestyle Profile in Each BMI Category

Healthy lifestyle factor				Overall population (N = 438 583)		BMI 18.5-24.9 (n = 144 579)			BMI 25.0-29.9 (n = 186 963)			BMI ≥30.0 (n = 107 041)	
Physically active	No or moderate alcohol consumption	Never smoking	Healthy diet	Adjusted HR (95% CI)^a^^,^^b^	*P* value	Adjusted HR (95% CI)^a^^,^^b^	*P* value	Adjusted HR (95% CI)^a^^,^^b^		*P* value	Adjusted HR (95% CI)^a^^,^^b^		*P* value
No	No	No	No	1 [Reference]	NA	1 [Reference]	NA	1 [Reference]		NA	1 [Reference]		NA
No	No	No	Yes	0.84 (0.80-0.88)	<.001	0.70 (0.64-0.78)	<.001	0.81 (0.76-0.87)		<.001	0.93 (0.87-1.00)		>.99
No	No	Yes	No	0.93 (0.90-0.97)	.005	0.88 (0.81-0.95)	.04	0.88 (0.84-0.94)		<.001	1.01 (0.95-1.07)		>.99
No	No	Yes	Yes	0.79 (0.76-0.82)	<.001	0.70 (0.64-0.76)	<.001	0.76 (0.71-0.81)		<.001	0.87 (0.82-0.93)		.002
No	Yes	No	No	1.10 (1.06-1.14)	<.001	1.04 (0.95-1.13)	>.99	1.08 (1.02-1.14)		.10	1.17 (1.10-1.23)		<.001
No	Yes	No	Yes	0.87 (0.87-0.90)	<.001	0.71 (0.65-0.77)	<.001	0.85 (0.81-0.90)		<.001	0.97 (0.91-1.02)		>.99
No	Yes	Yes	No	1.03 (0.99-1.06)	>.99	0.92 (0.85-1.00)	.88	1.00 (0.95-1.05)		>.99	1.12 (1.06-1.18)		<.001
No	Yes	Yes	Yes	0.88 (0.85-0.91)	<.001	0.73 (0.68-0.78)	<.001	0.85 (0.81-0.90)		<.001	0.99 (0.94-1.04)		>.99
Yes	No	No	No	0.88 (0.85-0.91)	<.001	0.82 (0.76-0.88)	<.001	0.86 (0.82-0.90)		<.001	0.93 (0.88-0.98)		.15
Yes	No	No	Yes	0.73 (0.70-0.76)	<.001	0.62 (0.57-0.67)	<.001	0.71 (0.67-0.75)		<.001	0.84 (0.78-0.89)		<.001
Yes	No	Yes	No	0.81 (0.78-0.83)	<.001	0.69 (0.64-0.73)	<.001	0.81 (0.77-0.85)		<.001	0.90 (0.85-0.95)		<.001
Yes	No	Yes	Yes	0.70 (0.68-0.72)	<.001	0.61 (0.57-0.65)	<.001	0.69 (0.65-0.72)		<.001	0.80 (0.75-0.85)		<.001
Yes	Yes	No	No	0.95 (0.92-0.98)	.050	0.84 (0.78-0.91)	<.001	0.95 (0.90-1.00)		>.99	1.00 (0.95-1.06)		>.99
Yes	Yes	No	Yes	0.77 (0.74-0.79)	<.001	0.64 (0.60-0.69)	<.001	0.75 (0.71-0.79)		<.001	0.88 (0.83-0.93)		<.001
Yes	Yes	Yes	No	0.88 (0.85-0.91)	<.001	0.76 (0.71-0.82)	<.001	0.86 (0.82-0.90)		<.001	0.98 (0.93-1.03)		>.99
Yes	Yes	Yes	Yes	0.76 (0.73-0.78)	<.001	0.64 (0.60-0.68)	<.001	0.75 (0.71-0.78)		<.001	0.88 (0.84-0.92)		<.001

Abbreviations: BMI, body mass index (calculated as weight in kilograms divided by height in meters squared); HR, hazard ratio; NA, not applicable.

^a^ Hazard ratios adjusted for age, sex, BMI, and Townsend deprivation index.

^b^ The HRs are for all obesity-associated diseases.

The association between obesity and the incidence of diseases at each level of the healthy lifestyle score is shown in Table 3. For each lifestyle score, adults with obesity were at higher risk of several primary outcomes compared with those with normal weight and the healthiest lifestyle score (ie, meeting all 4 healthy lifestyle factors) (HRs adjusted for age, sex, and Townsend deprivation index ranged from 1.41 [95% CI, 1.27-1.56] for arrhythmias to 7.16 [95% CI, 6.36-8.05] for diabetes in adults with obesity and 4 healthy lifestyle factors). The HRs for diseases in adults with overweight at each level of the healthy lifestyle score compared with those with normal weight and the healthiest lifestyle score are shown in eTable 7 in Supplement 1. Adults with obesity were at higher risk of all-cause mortality compared with those with healthy weight and the healthiest lifestyle score, although the relative increase in risk was smaller for adults with the highest lifestyle score (eTable 8 in Supplement 1). Among adults with obesity, the adjusted HRs for all-cause mortality were 1.37 (95% CI, 1.19-1.57) when meeting the 4 lifestyle factors and 2.43 (95% CI, 2.06-2.86) with 0 lifestyle factors. All the results were not substantially changed in the sensitivity analysis after exclusion of participants with diseases diagnosed within the first 2 years of follow-up (eTables 9-11 and eFigures 7-10 in Supplement 1).

**Table 3.** Associations of Obesity as Measured by BMI With Incident Obesity-Related Diseases by Healthy Lifestyle Score

Disease	Healthy lifestyle score^a^											
	4		3		2			1			0	
	Adjusted HR (95% CI)^b^	*P* value	Adjusted HR (95% CI)^b^	*P* value	Adjusted HR (95% CI)^b^	*P* value	Adjusted HR (95% CI)^b^		*P* value	Adjusted HR (95% CI)^b^		*P* value
**Infections**												
BMI 18.5-24.9	1 [Reference]	NA	1.13 (1.04-1.23)	.06	1.28 (1.18-1.40)	<.001	1.56 (1.42-1.72)		<.001	1.86 (1.57-2.19)		<.001
BMI ≥30.0	1.46 (1.32-1.62)	<.001	1.66 (1.53-1.81)	<.001	1.75 (1.61-1.90)	<.001	1.87 (1.70-2.05)		<.001	1.68 (1.44-1.95)		<.001
**Diabetes**												
BMI 18.5-24.9	1 [Reference]	NA	1.17 (1.03-1.33)	>.99	1.15 (1.01-1.31)	.59	1.19 (1.02-1.40)		.45	1.51 (1.16-1.96)		.04
BMI ≥30.0	7.16 (6.36-8.05)	<.001	7.76 (6.95-8.66)	<.001	7.71 (6.91-8.61)	<.001	7.91 (7.07-8.86)		<.001	7.87 (6.87-9.02)		<.001
**Esophageal adenocarcinoma**												
BMI 18.5-24.9	1 [Reference]	NA	1.27 (0.73-2.21)	>.99	2.15 (1.27-3.64)	.07	2.61 (1.47-4.63)		.02	4.49 (2.12-9.53)		.002
BMI ≥30.0	1.69 (0.87-3.28)	>.99	1.86 (1.08-3.20)	.43	3.16 (1.9-5.23)	<.001	3.46 (2.01-5.87)		<.001	2.57 (1.21-5.45)		.24
**Gastric cardia cancer**												
BMI 18.5-24.9	1 [Reference]	NA	1.51 (0.64-3.60)	>.99	1.64 (0.69-3.94)	>.99	2.21 (0.85-5.70)		>.99	5.16 (1.64-16.30)		.09
BMI ≥30.0	2.99 (1.16-7.71)	.40	3.02 (1.33-6.88)	.14	3.87 (1.74-8.56)	.01	3.17 (1.69-7.42)		.13	2.24 (0.66-7.68)		>.99
**Pancreatic cancer**												
BMI 18.5-24.9	1 [Reference]	NA	0.92 (0.62-1.37)	>.99	1.20 (0.81-1.78)	>.99	1.67 (1.07-2.59)		.39	1.40 (0.59-3.30)		>.99
BMI ≥30.0	0.83 (0.48-1.46)	>.99	1.50 (1.01-2.20)	.71	1.55 (1.06-2.27)	.42	1.40 (0.90-2.17)		>.99	1.19 (0.56-2.56)		>.99
**Liver cancer**												
BMI 18.5-24.9	1 [Reference]	NA	1.74 (0.93-3.24)	>.99	1.70 (0.90-3.22)	>.99	2.50 (1.25-5.00)		.16	3.87 (1.47-10.20)		.10
BMI ≥30.0	2.33 (1.13-4.80)	.37	2.06 (1.09-3.89)	.43	3.67 (2.03-6.63)	<.001	3.45 (1.83-6.51)		.002	1.59 (0.52-4.90)		>.99
**Colorectal cancer**												
BMI 18.5-24.9	1 [Reference]	NA	1.04 (0.87-1.24)	>.99	1.16 (0.97-1.39)	>.99	1.3 (1.05-1.62)		.29	1.07 (0.69-1.67)		>.99
BMI ≥30.0	1.25 (0.99-1.57)	.99	1.19 (0.99-1.44)	>.99	1.35 (1.13-1.61)	.02	1.61 (1.32-1.95)		<.001	1.65 (1.22-2.24)		.02
**Breast cancer (in women)**												
BMI 18.5-24.9	1 [Reference]	NA	1.11 (0.99-1.24)	>.99	1.17 (1.04-1.31)	.14	1.29 (1.12-1.50)		.009	1.19 (0.87-1.62)		>.99
BMI ≥30.0	1.23 (1.06-1.43)	.10	1.24 (1.09-1.40)	.01	1.40 (1.24-1.59)	<.001	1.44 (1.24-1.68)		<.001	1.49 (1.12-1.99)		.11
**Ovarian cancer (in women)**												
BMI 18.5-24.9	1 [Reference]	NA	0.90 (0.66-1.24)	>.99	0.83 (0.59-1.16)	>.99	0.78 (0.49-1.24)		>.99	0.41 (0.10-1.66)		>.99
BMI ≥30.0	1.14 (0.76-1.70)	>.99	1.00 (0.70-1.40)	>.99	0.93 (0.65-1.34)	>.99	1.11 (0.71-1.72)		>.99	0.83 (0.30-2.29)		>.99
**Endometrial cancer (in women)**												
BMI 18.5-24.9	1 [Reference]	NA	0.96 (0.68-1.35)	>.99	0.68 (0.46-0.99)	.77	0.86 (0.53-1.40)		>.99	0 (0-∞)		>.99
BMI ≥30.0	2.64 (1.86-3.75)	<.001	2.91 (2.13-3.97)	<.001	2.73 (1.99-3.75)	<.001	1.98 (1.32-2.96)		.02	1.51 (0.65-3.51)		>.99
**Kidney cancer**												
BMI 18.5-24.9	1 [Reference]	NA	1.33 (0.86-2.07)	>.99	1.15 (0.72-1.83)	>.99	1.52 (0.90-2.59)		>.99	2.47 (1.13-5.43)		.41
BMI ≥30.0	2.14 (1.29-3.55)	.06	2.44 (1.59-3.73)	<.001	3.14 (2.09-4.74)	<.001	3.15 (2.03-4.89)		<.001	2.08 (1.03-4.19)		.68
**Hypertension**												
BMI 18.5-24.9	1 [Reference]	NA	1.02 (0.97-1.08)	>.99	1.15 (1.09-1.21)	<.001	1.41 (1.32-1.49)		<.001	1.76 (1.59-1.95)		<.001
BMI ≥30.0	2.67 (2.53-2.82)	<.001	2.82 (2.69-2.95)	<.001	2.87 (2.74-3.01)	<.001	3.15 (3.00-3.32)		<.001	3.16 (2.94-3.39)		<.001
**Ischemic heart disease**												
BMI 18.5-24.9	1 [Reference]	NA	1.08 (0.99-1.17)	>.99	1.22 (1.13-1.32)	<.001	1.53 (1.40-1.68)		<.001	1.92 (1.65-2.22)		<.001
BMI ≥30.0	2.10 (1.92-2.30)	<.001	2.44 (2.26-2.63)	<.001	2.69 (2.50-2.90)	<.001	2.90 (2.69-3.14)		<.001	2.91 (2.61-3.24)		<.001
**Pulmonary embolism**												
BMI 18.5-24.9	1 [Reference]	NA	1.05 (0.83-1.32)	>.99	1.15 (0.91-1.45)	>.99	1.68 (1.30-2.19)		.002	2.48 (1.67-3.70)		<.001
BMI ≥30.0	2.57 (2.01-3.29)	<.001	2.52 (2.03-3.12)	<.001	2.70 (2.19-3.33)	<.001	2.98 (2.38-3.74)		<.001	2.54 (1.81-3.56)		<.001
**Arrhythmias**												
BMI 18.5-24.9	1 [Reference]	NA	0.98 (0.90-1.07)	>.99	1.08 (0.99-1.18)	>.99	1.21 (1.09-1.35)		.005	1.43 (1.19-1.72)		.002
BMI ≥30.0	1.41 (1.27-1.56)	<.001	1.74 (1.60-1.89)	<.001	1.86 (1.72-2.02)	<.001	2.09 (1.92-2.28)		<.001	2.00 (1.75-2.28)		<.001
**Heart failure**												
BMI 18.5-24.9	1 [Reference]	NA	1.06 (0.88-1.27)	>.99	1.32 (1.10-1.58)	.04	1.74 (1.43-2.13)		<.001	2.29 (1.68-3.12)		<.001
BMI ≥30.0	2.39 (1.97-2.90)	<.001	2.82 (2.39-3.32)	<.001	3.49 (2.97-4.10)	<.001	3.88 (3.28-4.59)		<.001	3.67 (2.94-4.59)		<.001
**Cerebrovascular diseases**												
BMI 18.5-24.9	1 [Reference]	NA	0.94 (0.83-1.08)	>.99	1.18 (1.04-1.35)	.19	1.50 (1.30-1.74)		<.001	1.98 (1.56-2.51)		<.001
BMI ≥30.0	1.22 (1.03-1.43)	.31	1.55 (1.37-1.76)	<.001	1.62 (1.44-1.84)	<.001	1.80 (1.58-2.06)		<.001	1.63 (1.32-2.01)		<.001
**Arteriosclerosis**												
BMI 18.5-24.9	1 [Reference]	NA	1.05 (0.49-2.26)	>.99	2.23 (1.11-4.49)	.42	4.50 (2.21-9.14)		<.001	8.27 (3.57-19.19)		<.001
BMI ≥30.0	0.83 (0.28-2.43)	>.99	3.02 (1.52-5.99)	.03	2.48 (1.25-4.94)	.16	4.14 (2.07-8.27)		<.001	4.45 (1.88-10.50)		.01
**Deep vein thrombosis**												
BMI 18.5-24.9	1 [Reference]	NA	0.85 (0.69-1.05)	>.99	1.03 (0.84-1.27)	>.99	1.26 (0.98-1.61)		>.99	1.81 (1.22-2.68)		.050
BMI ≥30.0	1.74 (1.37-2.20)	<.001	1.81 (1.49-2.21)	<.001	1.92 (1.58-2.33)	<.001	1.99 (1.61-2.46)		<.001	2.31 (1.70-3.13)		<.001
**Asthma**												
BMI 18.5-24.9	1 [Reference]	NA	1.02 (0.94-1.10)	>.99	1.05 (0.97-1.13)	>.99	1.17 (1.06-1.30)		.02	1.53 (1.30-1.82)		<.001
BMI ≥30.0	1.70 (1.55-1.87)	<.001	1.79 (1.65-1.93)	<.001	1.87 (1.73-2.01)	<.001	1.93 (1.77-2.10)		<.001	1.78 (1.55-2.05)		<.001
**Liver disease**												
BMI 18.5-24.9	1 [Reference]	NA	1.19 (0.98-1.45)	>.99	1.60 (1.32-1.95)	<.001	2.46 (1.99-3.03)		<.001	3.05 (2.23-4.18)		<.001
BMI ≥30.0	3.13 (2.55-3.84)	<.001	3.50 (2.92-4.19)	<.001	3.91 (3.27-4.67)	<.001	4.51 (3.74-5.43)		<.001	4.18 (3.25-5.38)		<.001
**Kidney failure**												
BMI 18.5-24.9	1 [Reference]	NA	0.99 (0.88-1.12)	>.99	1.23 (1.09-1.38)	.01	1.62 (1.41-1.85)		<.001	1.88 (1.51-2.36)		<.001
BMI ≥30.0	2.35 (2.07-2.67)	<.001	2.73 (2.45-3.04)	<.001	3.07 (2.76-3.41)	<.001	3.36 (3.00-3.76)		<.001	3.17 (2.71-3.71)		<.001
**Gout**												
BMI 18.5-24.9	1 [Reference]	NA	1.30 (0.82-2.05)	>.99	1.74 (1.11-2.72)	.26	2.13 (1.30-3.49)		.04	3.40 (1.75-6.62)		.005
BMI ≥30.0	6.58 (4.25-10.17)	<.001	7.59 (5.08-11.34)	<.001	9.86 (6.64-14.65)	<.001	11.66 (7.82-17.40)		<.001	12.25 (7.89-19.02)		<.001
**Osteoarthritis**												
BMI 18.5-24.9	1 [Reference]	NA	1.10 (1.02-1.17)	.13	1.16 (1.08-1.24)	<.001	1.28 (1.18-1.40)		<.001	1.15 (0.97-1.36)		>.99
BMI ≥30.0	2.95 (2.75-3.16)	<.001	3.12 (2.94-3.32)	<.001	3.24 (3.05-3.45)	<.001	3.27 (3.06-3.50)		<.001	3.33 (3.02-3.68)		<.001
**Sleep disorders**												
BMI 18.5-24.9	1 [Reference]	NA	1.06 (0.81-1.38)	>.99	1.05 (0.80-1.37)	>.99	1.41 (1.04-1.91)		.48	1.43 (0.84-2.45)		>.99
BMI ≥30.0	6.12 (4.79-7.83)	<.001	6.67 (5.32-8.37)	<.001	7.60 (6.07-9.51)	<.001	8.60 (6.83-10.83)		<.001	8.98 (6.86-11.74)		<.001
**Mood disorders**												
BMI 18.5-24.9	1 [Reference]	NA	1.24 (1.12-1.39)	<.001	1.49 (1.34-1.65)	<.001	1.98 (1.76-2.24)		<.001	2.50 (2.06-3.03)		<.001
BMI ≥30.0	2.13 (1.89-2.41)	<.001	2.59 (2.34-2.87)	<.001	2.80 (2.53-3.10)	<.001	3.08 (2.76-3.44)		<.001	3.17 (2.70-3.72)		<.001

Abbreviations: BMI, body mass index (calculated as weight in kilograms divided by height in meters squared); HR, hazard ratio; NA, not applicable.

^a^ Healthy lifestyle score included 4 lifestyle factors (never smoking, no or moderate alcohol consumption, physical activity, and healthy diet). Participants scored 1 if they met the criterion for a healthy lifestyle and 0 otherwise.

^b^ The HRs are adjusted for age, sex, and Townsend deprivation index.

## Discussion

The findings from this population-based cohort study suggest that adherence to a healthy lifestyle as a composite score is associated with a reduced risk of several health outcomes in adults with obesity but does not entirely offset the negative effects of obesity. Studies that estimate the risk of a wide range of outcomes according to adherence to lifestyle factors among adults with obesity seem to be lacking. Most of the research has focused on cardiovascular diseases and mortality and individual lifestyle factors.

The novelty of our study is the examination of the association between combinations of lifestyle factors (adequate physical activity, never smoking, no or moderate alcohol consumption) and the incidence of several comorbid diseases. Our results confirmed the lower risk of mortality and cardiovascular disease in adults with obesity meeting the 4 healthy lifestyle factors^6,17,19^ and extended the findings to kidney failure, gout, sleep disorders, and mood disorders. The association between a healthy lifestyle and diseases was independent of other potential confounders, such as age, sex, and socioeconomic status. In addition, we observed a reduced risk of diabetes, liver disease, and osteoarthritis, though the interactions were not statistically significant after the Bonferroni correction. The lifestyle profiles that were associated with the lowest risk of developing diseases included a healthy diet and at least 1 of the 2 healthy behaviors of adequate physical activity and never smoking. Tobacco affects health mainly via increasing DNA damage and oxidative stress, and smokers with obesity have been shown to be at higher risk of morbidity and premature mortality compared with nonsmokers without obesity.^11^ The health benefits of a healthy diet and physical activity include reduced insulin resistance, oxidative stress, and inflammation and improvements in lipid lipoprotein profiles.^30,31,32^

Although adhering to a healthy lifestyle was associated with a reduced risk of health outcomes, adults with obesity still had an increased risk of diseases compared with those with a normal BMI and meeting the 4 lifestyle factors. Obesity is a strong risk factor for comorbidities, whereas not smoking, reducing alcohol intake, exercising regularly, and eating a healthy diet may not be sufficient to attenuate the risk. Many studies have highlighted the potential benefits of weight loss in preventing comorbidities among adults with obesity.^33^ Thus, supporting people to reduce their body weight in addition to promoting healthy behaviors may bring additional benefits to reduce the risk of developing comorbid diseases and extend disease-free life expectancy in obesity.

This study is the first in our knowledge to assess the association of combined healthy lifestyle factors with a wide range of obesity-related outcomes in adults with obesity. Our findings have important clinical and public health implications. Individual counseling by clinicians and national-level public policies to decrease smoking, moderate alcohol consumption, increase exercise frequency, adhere to a healthy diet, and reduce body weight are essential to reduce the burden of obesity.

### Limitations

This study has several limitations. First, the study population may not be completely representative of the UK population because participants are more likely to have a healthy lifestyle,^34^ which might lead to an underestimation of health hazards in individuals with the lowest healthy lifestyle score.^34^ Second, we used measures of lifestyle behavior at baseline and did not consider changes over the follow-up period. Third, although lifestyle factors may have different effects on obesity-related diseases, we did not weigh them by their association with the outcomes in our analysis. We wanted to evaluate overall lifestyle as a cluster, and the weighted score cannot fully account for the complex interactions between lifestyle factors. Fourth, although our study included data on infectious diseases, we did not differentiate among them, including SARS-CoV-2 infection (COVID-19), and we did not specifically evaluate the impact of healthy lifestyle on COVID-19 morbidity and mortality. Obesity is associated with an increased risk of COVID-19 complications and mortality,^35^ and investigating the specific association of a healthy lifestyle with COVID-19 outcomes in patients with a high BMI is of particular interest. Fifth, despite the exclusion of participants with diseases diagnosed within the first 2 years of follow-up, reverse causality and residual confounding remain limitations because of the nature of observational studies.

## Conclusions

In this cohort study of UK Biobank participants, adherence to a healthy lifestyle as a composite score, including not smoking, exercising regularly, consuming no or moderate amounts of alcohol, and eating a healthy diet, was associated with a reduced risk of several health outcomes among adults with obesity. Although a healthy lifestyle appeared to be beneficial, it did not entirely offset the health risks associated with obesity.

## Notes

Supplement 1. **eTable 1.** Codes Used to Define the Variables Included in the Study **eTable 2.** Definition and Scoring of Lifestyle Factors **eTable 3.** *International Classification of Diseases, Tenth Revision* (*ICD-10*) Codes for Diseases Included in the Study **eTable 4.** Hazard Ratios for Incident Diseases by Lifestyle Profile in Each Body Mass Index Category **eTable 5.** Hazard Ratios for All-Cause Mortality by Lifestyle Profile in Each Body Mass Index Category **eTable 6.** Adjusted Hazard Ratios for All-Cause Mortality by Lifestyle Profile in Each Body Mass Index Category **eTable 7.** Associations of Overweight With Incident Diseases by Healthy Lifestyle Score **eTable 8.** Associations of Body Mass Index With All-Cause Mortality by Healthy Lifestyle Score **eFigure 1.** Flowchart of Sample Selection **eFigure 2.** Hazard Ratios for Incident Diseases by Body Mass Index Category **eFigure 3.** Adjusted Hazard Ratios for Incident Diseases by Body Mass Index Category **eFigure 4.** A. Hazard Ratios for Incident Diseases by Lifestyle Profile in Individuals With Normal Weight (BMI 1.5-24.9), B. Overweight (BMI 25-29.9), and C. Obesity (BMI ≥30) **eFigure 5.** Adjusted Hazard Ratios for Incident Diseases by Lifestyle Profile in Individuals With Overweight (BMI 25-29.9) **eFigure 6.** Hazard Ratios for All-Cause Mortality by Lifestyle Profile in Individuals With Normal Weight (BMI 18.5-24.9 kg/m^2^), Overweight (BMI 25-29.9), and Obesity (BMI ≥30) Before (A) and After Adjustment (B) for Age, Sex, and Townsend Deprivation Index **eTable 9.** Associations of Body Mass Index With Incident Diseases by Healthy Lifestyle Score After Exclusion of Participants With Diseases Diagnosed Within the First 2 Years of Follow-up **eTable 10.** Hazard Ratios for Incident Diseases by Lifestyle Profile in Each Body Mass Index Category After Exclusion of Participants With Diseases Diagnosed With the First 2 Years of Follow-up **eTable 11.** Adjusted Hazard Ratios for Incident Diseases by Lifestyle Profile in Each Body Mass Index Category After Exclusion of Participants With Diseases Diagnosed With the First 2 Years of Follow-up **eFigure 7.** Hazard Ratios for Incident Diseases by Body Mass Index Category After Exclusion of Participants With Diseases Diagnosed Within the First 2 Years of Follow-up **eFigure 8.** Adjusted Hazard Ratios for Incident Diseases by Body Mass Index Category After Exclusion of Participants With Diseases Diagnosed Within the First 2 Years of Follow-up **eFigure 9.** Hazard Ratios for Incident Diseases by Lifestyle Profile in Individuals With Normal Weight (BMI 18.5-24.9) (A), Overweight (BMI 25-29.9) (B), and Obesity (BMI ≥30) (C) After Exclusion of Participants With Diseases Diagnosed Within the First 2 Years of Follow-up **eFigure 10.** Adjusted Hazard Ratios for Incident Diseases by Lifestyle Profile in Individuals With Normal Weight (BMI 18.5-24.9) (A), Overweight (BMI 25-29.9) (B), and Obesity (BMI ≥30) (C) After Exclusion of Participants With Diseases Diagnosed Within the First 2 Years of Follow-up **eFigure 11. **Association of Healthy Lifestyle Score With Incident Diseases in Adults With a Healthy Weight **eFigure 12.** Association of Healthy Lifestyle Score With Incident Diseases in Adults With Obesity

Supplement 2.
Data Sharing Statement
