# Supplementary material for: Association of Healthy Lifestyle Factors and Obesity-Related Diseases in Adults in the UK
Source: JAMA Netw Open. 2023 May 26;6(5):e2314741. doi: 10.1001/jamanetworkopen.2023.14741 (PMC10220514; doi:10.1001/jamanetworkopen.2023.14741)
Supplement: Supplement 1. — eTable 1. Codes Used to Define the Variables Included in the Study eTable 2. Definition and Scoring of Lifestyle Factors eTable 3. International Classification of Diseases, Tenth Revision (ICD-10) Codes for Diseases Included in the Study eTable 4. Hazard Ratios for Incident Diseases by Lifestyle Profile in Each Body Mass Index Category eTable 5. Hazard Ratios for All-Cause Mortality by Lifestyle Profile in Each Body Mass Index Category eTable 6. Adjusted Hazard Ratios for All-Cause Mortality by Lifestyle Profile in Each Body Mass Index Category eTable 7. Associations of Overweight With Incident Diseases by Healthy Lifestyle Score eTable 8. Associations of Body Mass Index With All-Cause Mortality by Healthy Lifestyle Score eFigure 1. Flowchart of Sample Selection eFigure 2. Hazard Ratios for Incident Diseases by Body Mass Index Category eFigure 3. Adjusted Hazard Ratios for Incident Diseases by Body Mass Index Category eFigure 4. A. Hazard Ratios for Incident Diseases by Lifestyle Profile in Individuals With Normal Weight (BMI 1.5-24.9), B. Overweight (BMI 25-29.9), and C. Obesity (BMI ≥30) eFigure 5. Adjusted Hazard Ratios for Incident Diseases by Lifestyle Profile in Individuals With Overweight (BMI 25-29.9) eFigure 6. Hazard Ratios for All-Cause Mortality by Lifestyle Profile in Individuals With Normal Weight (BMI 18.5-24.9 kg/m2), Overweight (BMI 25-29.9), and Obesity (BMI ≥30) Before (A) and After Adjustment (B) for Age, Sex, and Townsend Deprivation Index eTable 9. Associations of Body Mass Index With Incident Diseases by Healthy Lifestyle Score After Exclusion of Participants With Diseases Diagnosed Within the First 2 Years of Follow-up eTable 10. Hazard Ratios for Incident Diseases by Lifestyle Profile in Each Body Mass Index Category After Exclusion of Participants With Diseases Diagnosed With the First 2 Years of Follow-up eTable 11. Adjusted Hazard Ratios for Incident Diseases by Lifestyle Profile in Each Body Mass Index Category After Exclusion of Partici [file jamanetwopen-e2314741-s001.pdf]

Supplemental Online Content

Rassy N, Van Straaten A, Carette C, Hamer M, Rives-Lange C, Czernichow S. Association of healthy lifestyle factors and obesity-related diseases in adults in the UK. *JAMA Netw Open*. 2023;6(5):e2314741. doi:10.1001/jamanetworkopen.2023.14741

eTable 1. Codes Used to Define the Variables Included in the Study

eTable 2. Definition and Scoring of Lifestyle Factors

eTable 3. *International Classification of Diseases, Tenth Revision* (*ICD-10*) Codes for Diseases Included in the Study

eTable 4. Hazard Ratios for Incident Diseases by Lifestyle Profile in Each Body Mass Index Category

eTable 5. Hazard Ratios for All-Cause Mortality by Lifestyle Profile in Each Body Mass Index Category

eTable 6. Adjusted Hazard Ratios for All-Cause Mortality by Lifestyle Profile in Each Body Mass Index Category

eTable 7. Associations of Overweight With Incident Diseases by Healthy Lifestyle Score

eTable 8. Associations of Body Mass Index With All-Cause Mortality by Healthy Lifestyle Score

eFigure 1. Flowchart of Sample Selection

eFigure 2. Hazard Ratios for Incident Diseases by Body Mass Index Category

eFigure 3. Adjusted Hazard Ratios for Incident Diseases by Body Mass Index Category

eFigure 4. A. Hazard Ratios for Incident Diseases by Lifestyle Profile in Individuals With Normal Weight (BMI 1.5-24.9), B. Overweight (BMI 25-29.9), and C. Obesity (BMI ≥30)

eFigure 5. Adjusted Hazard Ratios for Incident Diseases by Lifestyle Profile in Individuals With Overweight (BMI 25-29.9)

eFigure 6. Hazard Ratios for All-Cause Mortality by Lifestyle Profile in Individuals With Normal Weight (BMI 18.5-24.9 kg/m^2), Overweight (BMI 25-29.9), and Obesity (BMI ≥30) Before (A) and After Adjustment (B) for Age, Sex, and Townsend Deprivation Index

eTable 9. Associations of Body Mass Index With Incident Diseases by Healthy Lifestyle Score After Exclusion of Participants With Diseases Diagnosed Within the First 2 Years of Follow-up

eTable 10. Hazard Ratios for Incident Diseases by Lifestyle Profile in Each Body Mass Index Category After Exclusion of Participants With Diseases Diagnosed With the First 2 Years of Follow-up

© 2023 Rassy N et al. *JAMA Network Open*.

eTable 11. Adjusted Hazard Ratios for Incident Diseases by Lifestyle Profile in Each Body Mass Index Category After Exclusion of Participants With Diseases Diagnosed With the First 2 Years of Follow-up

eFigure 7. Hazard Ratios for Incident Diseases by Body Mass Index Category After Exclusion of Participants With Diseases Diagnosed Within the First 2 Years of Follow-up

eFigure 8. Adjusted Hazard Ratios for Incident Diseases by Body Mass Index Category After Exclusion of Participants With Diseases Diagnosed Within the First 2 Years of Follow-up

eFigure 9. Hazard Ratios for Incident Diseases by Lifestyle Profile in Individuals With Normal Weight (BMI 18.5-24.9) (A), Overweight (BMI 25-29.9) (B), and Obesity (BMI ≥30) (C) After Exclusion of Participants With Diseases Diagnosed Within the First 2 Years of Follow-up

eFigure 10. Adjusted Hazard Ratios for Incident Diseases by Lifestyle Profile in Individuals With Normal Weight (BMI 18.5-24.9) (A), Overweight (BMI 25-29.9) (B), and Obesity (BMI ≥30) (C) After Exclusion of Participants With Diseases Diagnosed Within the First 2 Years of Follow-up

eFigure 11. Association of Healthy Lifestyle Score With Incident Diseases in Adults With a Healthy Weight

eFigure 12. Association of Healthy Lifestyle Score With Incident Diseases in Adults With Obesity

This supplemental material has been provided by the authors to give readers additional information about their work.

© 2023 Rassy N et al. *JAMA Network Open*.

eTable 1. Codes Used to Define the Variables Included in the Study

Variable	Field IDs
Age at recruitment	21022
Sex	31
Townsend deprivation index	189
Body mass index	21001
Diagnoses – main and secondary ICD10	41270
Date of first in-patient diagnosis in either the primary or secondary position in the participant's hospital inpatient records ICD10	41280
Smoking status	20116
Alcohol intake (champagne, white wine, beer, cider, spirits, fortified wine, and other types of alcohol consumption)	1568, 1578, 1588, 1598, 1608, 5364
Cooked and raw vegetables intake	1289, 1299
Fresh and dried fruit intake	1309, 1319
Bread intake and type	1448, 1438
Cereal intake and type	1458, 1468
Oily and non-oily fish	1329, 1339
Cheese and milk intake	1408, 1418
Vegetable oils intake	1428, 2654
Poultry, beef, lamb, pork, processed meat intake	1359, 1369, 1379, 1389, 1349
Sugar-sweetened beverages intake	6144
Days/week of vigorous physical activity 10+ minutes<br>Duration of vigorous activity	904<br>914
Days/week of moderate physical activity 10+ minutes<br>Duration of moderate activity	884<br>894
Number of days/week walked 10+ minutes<br>Duration of walks	864<br>874
ICD: International Classification of Disease version 10	

© 2023 Rassy N et al. *JAMA Network Open*.

eTable 2. Definition and Scoring of Lifestyle Factors

Lifestyle factor	Score
**Physical activity[a]**	
≥2.5 hours of moderate activity/week or ≥1.25 hours of vigorous activity/week	1
<2.5 hours of moderate activity/week or <1.25 hours of vigorous activity/week	0
**Alcohol consumption**	
0-5 drink/week in women and 0-10 drink/week in men	1
> 5 drink/week in women and > 10 drink/week in men	0
**Smoking**	
Never smoker	1
Current or former smoker	0
**Healthy diet[b]**	
Adequate intake of ≥ 5 diet components	1
Adequate intake of < 5 diet components	0

[a] walking was considered moderate activity

[b] Components of dietary recommendations for cardiovascular health:
fruits: ≥ 3 servings/day; vegetables: ≥ 3 servings/day; whole grains: ≥ 3 servings/day; refined grains: ≤ 2 servings/day; fish and shellfish: ≥ 2 servings/week; dairy: ≥ 2 servings/day; vegetable oils: ≥ 2 servings/day; processed meats: ≤ 1 serving/week; unprocessed meats: ≤ 2 servings/week; Sugar sweetened beverages (including 100% fruit juice): no consumption

© 2023 Rassy N et al. *JAMA Network Open*.

eTable 3. ***International Classification of Diseases, Tenth Revision*** **(*ICD-10*) Codes for Diseases Included in the Study**

**Disease**	**ICD-10 code**
Bacterial infections	A01-A79
Viral infections	A80-B34
Diabetes	E10-E14
Esophageal adenocarcinoma	C15
Gastric cardia cancer	C160
Pancreatic cancer	C25
Liver cancer	C22
Colorectal cancer	C18-C19-C20
Breast cancer	C50
Ovarian cancer	C56
Endometrial cancer	C54
Kidney cancer	C64
Hypertension	I10-I15
Ischemic heart disease	I20-I25
Pulmonary embolism	I26
Arrhythmias	I46-I49
Heart failure	I50
Cerebrovascular diseases	I60-I69
Arteriosclerosis	I70
Deep vein thrombosis	I80-I82
Asthma	J45–J46
Liver disease	K70–K77
Renal failure	N17-N19
Gout	M10
Osteoarthritis	M15–M19
Sleep disorders	G47
Mood disorders	F30-F39

© 2023 Rassy N et al. *JAMA Network Open*.

eTable 4. Hazard Ratios for Incident Diseases by Lifestyle Profile in Each Body Mass Index Category

Healthy lifestyle factor				HR (95% CI)			
Physically active	No or moderate alcohol consumption	Never smoking	Healthy diet				
				Total population (n = 438 583)	BMI 18.5-24.9 (n = 144 579)	BMI 25-29.9 (n = 186 963)	BMI ≥ 30 (n = 107 041)
No	No	No	No	*Reference*	*Reference*	*Reference*	*Reference*
No	No	No	Yes	0.71 (0.68-0.74)	0.6 (0.55-0.67)	0.71 (0.66-0.76)	0.82 (0.77-0.88)
No	No	Yes	No	0.92 (0.89-0.96)	0.89 (0.82-0.97)	0.92 (0.87-0.97)	1.03 (0.97-1.09)
No	No	Yes	Yes	0.69 (0.67-0.72)	0.65 (0.60-0.71)	0.72 (0.68-0.77)	0.80 (0.75-0.86)
No	Yes	No	No	1.17 (1.13-1.22)	1.06 (0.98-1.16)	1.13 (1.07-1.19)	1.22 (1.16-1.29)
No	Yes	No	Yes	0.77 (0.75-0.8)	0.63 (0.58-0.68)	0.76 (0.72-0.81)	0.9 (0.85-0.95)
No	Yes	Yes	No	1.12 (1.08-1.16)	0.97 (0.9-1.05)	1.08 (1.02-1.14)	1.23 (1.17-1.30)
No	Yes	Yes	Yes	0.83 (0.80-0.86)	0.70 (0.65-0.75)	0.84 (0.80-0.88)	0.98 (0.93-1.03)
Yes	No	No	No	0.86 (0.83-0.88)	0.84 (0.78-0.91)	0.89 (0.85-0.94)	0.92 (0.87-0.98)
Yes	No	No	Yes	0.60 (0.58-0.63)	0.55 (0.51-0.60)	0.64 (0.61-0.68)	0.74 (0.69-0.79)
Yes	No	Yes	No	0.77 (0.74-0.79)	0.71 (0.66-0.76)	0.86 (0.82-0.9)	0.92 (0.87-0.97)
Yes	No	Yes	Yes	0.60 (0.58-0.62)	0.59 (0.55-0.63)	0.68 (0.64-0.71)	0.74 (0.69-0.79)
Yes	Yes	No	No	0.98 (0.95-1.01)	0.90 (0.83-0.97)	1.02 (0.97-1.07)	1.05 (0.99-1.10)
Yes	Yes	No	Yes	0.66 (0.64-0.69)	0.60 (0.56-0.65)	0.69 (0.66-0.73)	0.80 (0.76-0.85)
Yes	Yes	Yes	No	0.90 (0.88-0.93)	0.82 (0.76-0.88)	0.95 (0.90-0.99)	1.06 (1.01-1.11)
Yes	Yes	Yes	Yes	0.68 (0.66-0.71)	0.64 (0.60-0.68)	0.75 (0.72-0.78)	0.86 (0.8-0.90)

HR, hazard ratio; CI, confidence interval

© 2023 Rassy N et al. *JAMA Network Open*.

eTable 5. Hazard Ratios for All-Cause Mortality by Lifestyle Profile in Each Body Mass Index Category

Healthy lifestyle factor				HR (95% CI)			
**Physically active**	**No or moderate alcohol consumption**	**Never smoking**	**Healthy diet**				
				**Total population (n = 438 583)**	**BMI 18.5-24.9 (n = 144 579)**	**BMI 25-29.9 (n = 186 963)**	**BMI ≥ 30 (n = 107 041)**
No	No	No	No	*Reference*	*Reference*	*Reference*	*Reference*
No	No	No	Yes	0.38 (0.33-0.45)	0.26 (0.19-0.37)	0.40 (0.31-0.51)	0.49 (0.37-0.64)
No	No	Yes	No	0.72 (0.64-0.80)	0.58 (0.47-0.73)	0.73 (0.61-0.87)	0.84 (0.69-1.03)
No	No	Yes	Yes	0.39 (0.33-0.45)	0.32 (0.25-0.42)	0.37 (0.30-0.47)	0.48 (0.37-0.63)
No	Yes	No	No	1.19 (1.07-1.31)	1.15 (0.94-1.41)	1.20 (1.02-1.41)	1.19 (1.10-1.41)
No	Yes	No	Yes	0.45 (0.40-0.50)	0.32 (0.25-0.40)	0.47 (0.39-0.57)	0.54 (0.44-0.65)
No	Yes	Yes	No	1.02 (0.93-1.13)	0.93 (0.77-1.14)	0.95 (0.80-1.12)	1.16(0.98-1.37)
No	Yes	Yes	Yes	0.47 (0.42-0.52)	0.37 (0.30-0.46)	0.48 (0.41-0.57)	0.54 (0.45-0.65)
Yes	No	No	No	0.78 (0.71-0.86)	0.70 (0.58-0.85)	0.82 (0.70-0.95)	0.82 (0.68-0.99)
Yes	No	No	Yes	0.38 (0.33-0.43)	0.32 (0.25-0.4)	0.38 (0.31-0.46)	0.46 (0.36-0.59)
Yes	No	Yes	No	0.55 (0.50-0.60)	0.39 (0.32-0.47)	0.61 (0.53-0.71)	0.71 (0.59-0.84)
Yes	No	Yes	Yes	0.32 (0.28-0.35)	0.23 (0.19-0.28)	0.34 (0.29-0.40)	0.46 (0.38-0.57)
Yes	Yes	No	No	0.89 (0.81-0.99)	0.75 (0.62-0.92)	0.89 (0.76-1.04)	1.03 (0.87-1.22)
Yes	Yes	No	Yes	0.39 (0.35-0.44)	0.35 (0.28-0.42)	0.4 (0.34-0.48)	0.42 (0.35-0.52)
Yes	Yes	Yes	No	0.67 (0.61-0.74)	0.53 (0.44-0.63)	0.71(0.62-0.82)	0.78 (0.66-0.92)
Yes	Yes	Yes	Yes	0.37 (0.34-0.41)	0.28 (0.24-0.34)	0.40 (0.35-0.47)	0.47 (0.40-0.56)

HR, hazard ratio; CI, confidence interval

© 2023 Rassy N et al. *JAMA Network Open*.

eTable 6. Adjusted Hazard Ratios for All-Cause Mortality by Lifestyle Profile in Each Body Mass Index Category

Healthy lifestyle factor				HR (95% CI) [a]			
Physically active	No or moderate alcohol consumption	Never smoking	Healthy diet				
				Total population (n = 438 583)	BMI 18.5-24.9 (n = 144 579)	BMI 25-29.9 (n = 186 963)	BMI ≥ 30 (n = 107 041)
No	No	No	No	*Reference*	*Reference*	*Reference*	*Reference*
No	No	No	Yes	0.52 (0.44-0.61)	0.37 (0.26-0.53)	0.52 (0.41-0.67)	0.64 (0.49-0.83)
No	No	Yes	No	0.77 (0.68-0.86)	0.64 (0.51-0.80)	0.75 (0.63-0.90)	0.88 (0.72-1.07)
No	No	Yes	Yes	0.51 (0.44-0.59)	0.43 (0.33-0.56)	0.47 (0.37-0.59)	0.65 (0.50-0.84)
No	Yes	No	No	1.11 (1.01-1.23)	1.08 (0.89-1.33)	1.13 (0.96-1.32)	1.16 (0.98-1.38)
No	Yes	No	Yes	0.56 (0.50-0.63)	0.40 (0.31-0.51)	0.58 (0.48-0.70)	0.69 (0.56-0.84)
No	Yes	Yes	No	0.97 (0.88-1.08)	0.95 (0.78-1.15)	0.89 (0.76-1.05)	1.11 (0.94-1.32)
No	Yes	Yes	Yes	0.56 (0.50-0.63)	0.45 (0.36-0.55)	0.56 (0.47-0.66)	0.68 (0.56-0.82)
Yes	No	No	No	0.76 (0.69-0.84)	0.68 (0.56-0.82)	0.77 (0.66-0.89)	0.79 (0.66-0.95)
Yes	No	No	Yes	0.48 (0.43-0.55)	0.41 (0.32-0.52)	0.46 (0.38-0.56)	0.58 (0.46-0.74)
Yes	No	Yes	No	0.58 (0.53-0.64)	0.42 (0.35-0.50)	0.60 (0.52-0.69)	0.71 (0.60-0.85)
Yes	No	Yes	Yes	0.40 (0.36-0.45)	0.29 (0.23-0.35)	0.39 (0.33-0.47)	0.60 (0.48-0.74)
Yes	Yes	No	No	0.82 (0.74-0.90)	0.68 (0.56-0.82)	0.79 (0.68-0.93)	0.97 (0.82-1.15)
Yes	Yes	No	Yes	0.47 (0.42-0.53)	0.40 (0.33-0.49)	0.46 (0.39-0.55)	0.54 (0.44-0.66)
Yes	Yes	Yes	No	0.65 (0.59-0.71)	0.51 (0.43-0.61)	0.65 (0.56-0.76)	0.76 (0.64-0.89)
Yes	Yes	Yes	Yes	0.44 (0.40-0.48)	0.32 (0.27-0.38)	0.44 (0.38-0.51)	0.58 (0.49-0.69)

HR, hazard ratio; CI, confidence interval , BMI, Body mass index
[a] HR adjusted for age, sex, and Townsend deprivation index

© 2023 Rassy N et al. *JAMA Network Open*.

eTable 7. Associations of Overweight With Incident Diseases by Healthy Lifestyle Score

Diseases	Healthy lifestyle score Adjusted HR (95% CI)[a]				
	4	3	2	1	0
**Infections**					
BMI 18.5-24.9 kg/m²	1 (Reference)	1.13 (1.04-1.23)	1.28 (1.18-1.4)	1.56 (1.42-1.72)	1.86 (1.57-2.19)
BMI 25-29.9 kg/m²	1.19 (1.08-1.30)	1.24 (1.14-1.34)	1.35 (1.24-1.46)	1.40 (1.28-1.53)	1.61 (1.40-1.84)
**Diabetes**					
BMI 18.5-24.9 kg/m²	1 (Reference)	1.17 (1.03-1.33)	1.15 (1.01-1.31)	1.19 (1.02-1.4)	1.51 (1.16-1.96)
BMI 25-29.9 kg/m²	2.29 (2.02-2.59)	2.48 (2.21-2.77)	2.53 (2.26-2.84)	2.80 (2.48-3.15)	3.00 (2.56-3.52)
**Esophageal adenocarcinoma**					
BMI 18.5-24.9 kg/m²	1 (Reference)	1.27 (0.73-2.21)	2.15 (1.27-3.64)	2.61 (1.47-4.63)	4.49 (2.12-9.53)
BMI 25-29.9 kg/m²	1.01 (0.54-1.87)	1.58 (0.94-2.63)	2.21 (1.34-3.63)	3.17 (1.9-5.27)	3.49 (1.84-6.62)
**Gastric cardia cancer**					
BMI 18.5-24.9 kg/m²	1 (Reference)	1.51 (0.64-3.6)	1.64 (0.69-3.94)	2.21 (0.85-5.7)	5.16 (1.64-16.3)
BMI 25-29.9 kg/m²	0.98 (0.37-2.64)	1.73 (0.77-3.89)	2.26 (1.02-4.99)	2.66 (1.17-6.06)	2.03 (0.64-6.41)
**Pancreatic cancer**					
BMI 18.5-24.9 kg/m²	1 (Reference)	0.92 (0.62-1.37)	1.2 (0.81-1.78)	1.67 (1.07-2.59)	1.4 (0.59-3.30)
BMI 25-29.9 kg/m²	1.02 (0.67-1.56)	1.15 (0.80-1.66)	1.34 (0.93-1.92)	1.19 (0.79-1.81)	1.11 (0.55-2.22)
**Liver cancer**					
BMI 18.5-24.9 kg/m²	1 (Reference)	1.74 (0.93-3.24)	1.7 (0.9-3.22)	2.5 (1.25-5)	3.87 (1.47-10.2)
BMI 25-29.9 kg/m²	1.49 (0.75-2.94)	1.36 (0.74-2.53)	1.69 (0.93-3.1)	2.37 (1.26-4.45)	2.39 (0.99-5.78)
**Colorectal cancer**					
BMI 18.5-24.9 kg/m²	1 (Reference)	1.04 (0.87-1.24)	1.16 (0.97-1.39)	1.3 (1.05-1.62)	1.07 (0.69-1.67)
BMI 25-29.9 kg/m²	1.07 (0.88-1.3)	1.18 (0.99-1.39)	1.27 (1.07-1.5)	1.61 (1.35-1.93)	1.51 (1.14-1.99)
**Breast cancer**					
BMI 18.5-24.9 kg/m²	1 (Reference)	1.11 (0.99-1.24)	1.17 (1.04-1.31)	1.29 (1.12-1.5)	1.1 9 (0.87-1.62)
BMI 25-29.9 kg/m²	1.08 (0.94-1.23)	1.16 (1.03-1.3)	1.3 (1.16-1.46)	1.27 (1.10-1.46)	1.47 (1.13-1.91)
**Ovarian cancer**					
BMI 18.5-24.9 kg/m²	1 (Reference)	0.9 (0.66-1.24	0.83 (0.59-1.16)	0.78 (0.49-1.24)	0.41 (0.10-1.66)
BMI 25-29.9 kg/m²	0.86 (0.59-1.24)	0.95 (0.70-1.30)	0.97 (0.7-1.33)	1.21 (0.82-1.77)	1.78 (0.94-3.38)
**Endometrial cancer**					
BMI 18.5-24.9 kg/m²	1 (Reference)	0.96 (0.68-1.35)	0.68 (0.46-0.99)	0.86 (0.53-1.4)	0 (0-inf)
BMI 25-29.9 kg/m²	1.44 (1.01-2.05)	1.28 (0.93-1.77)	1.1 (0.78-1.55)	1.25 (0.83-1.89)	0.58 (0.18-1.86)
**Kidney cancer**					
BMI 18.5-24.9 kg/m²	1 (Reference)	1.33 (0.86-2.07)	1.15 (0.72-1.83)	1.52 (0.9-2.59)	2.47 (1.13-5.43)
BMI 25-29.9 kg/m²	1.57 (0.99-2.49)	1.69 (1.12-2.56)	2.2 (1.47-3.29)	2.34 (1.52-3.6)	1.66 (0.84-3.27)
**Hypertension**					
BMI 18.5-24.9 kg/m²	1 (Reference)	1.02 (0.97-1.08)	1.15 (1.09-1.21)	1.41 (1.32-1.49)	1.76 (1.59-1.95)
BMI 25-29.9 kg/m²	1.67 (1.58-1.75)	1.69 (1.61-1.77)	1.81 (1.73-1.89)	1.99 (1.89-2.09)	2.31 (2.15-2.48)
**Ischemic heart disease**					
BMI 18.5-24.9 kg/m²	1 (Reference)	1.08 (0.99-1.17)	1.22 (1.13-1.32)	1.53 (1.4-1.68)	1.92 (1.65-2.22)
BMI 25-29.9 kg/m²	1.49 (1.38-1.62)	1.59 (1.48-1.71)	1.73 (1.61-1.86)	1.88 (1.74-2.03)	2.00 (1.79-2.23)
**Pulmonary embolism**					
BMI 18.5-24.9 kg/m²	1 (Reference)	1.05 (0.83-1.32)	1.15 (0.91-1.45)	1.68 (1.30-2.19)	2.48 (1.67-3.70)
BMI 25-29.9 kg/m²	1.78 (1.42-2.24)	1.58 (1.28-1.95)	1.74 (1.41-2.15)	1.77 (1.41-2.22)	1.82 (1.29-2.56)

[a] HR adjusted for age, sex, and Townsend deprivation index, Healthy lifestyle score included 4 lifestyle factors (never smoking, no or moderate alcohol consumption, physical activity, healthy diet). Participants scored 1 if they met the criterion for a healthy lifestyle and 0 otherwise

© 2023 Rassy N et al. *JAMA Network Open*.

Diseases	Healthy lifestyle score<br>Adjusted HR (95% CI)[a]				
	4	3	2	1	0
**Arrhythmias**					
BMI 18.5-24.9 kg/m²<br>BMI 25-29.9 kg/m²	1 (Reference)<br>1.03 (0.94-1.13)	0.98 (0.90-1.07)<br>1.11 (1.12-1.32)	1.08 (0.99-1.18)<br>1.22 (1.12-1.32)	1.21 (1.09-1.35)<br>1.29 (1.18-1.41)	1.43 (1.19-1.72)<br>1.29 (1.12-1.48)
**Heart failure**					
BMI 18.5-24.9 kg/m²<br>BMI 25-29.9 kg/m²	1 (Reference)<br>1.27 (1.05-1.53)	1.06 (0.88-1.27)<br>1.32 (1.12-1.56)	1.32 (1.10-1.58)<br>1.55 (1.31-1.82)	1.74 (1.43-2.13)<br>2.05 (1.72-2.43)	2.29 (1.68-3.12)<br>2.19 (1.73-2.78)
**Cerebrovascular diseases**					
BMI 18.5-24.9 kg/m²<br>BMI 25-29.9 kg/m²	1 (Reference)<br>1.03 (0.9-1.19)	0.94 (0.83-1.08)<br>1.1 (0.97-1.24)	1.18 (1.04-1.35)<br>1.23 (1.09-1.38)	1.50 (1.30-1.74)<br>1.46 (1.28-1.66)	1.98 (1.56-2.51)<br>1.96 (1.63-2.34)
**Arteriosclerosis**					
BMI 18.5-24.9 kg/m²<br>BMI 25-29.9 kg/m²	1 (Reference)<br>0.79 (0.33-1.89)	1.05 (0.49-2.26)<br>1.52 (0.77-3.04)	2.23 (1.11-4.49)<br>2.28 (1.17-4.43)	4.5 (2.21-9.14)<br>4.45 (2.29-8.66)	8.27 (3.57-19.19)<br>2.83 (1.15-6.99)
**Deep vein thrombosis**					
BMI 18.5-24.9 kg/m²<br>BMI 25-29.9 kg/m²	1 (Reference)<br>1.34 (1.08-1.65)	0.85 (0.69-1.05)<br>1.19 (0.98-1.44)	1.03 (0.84-1.27)<br>1.2 (0.99-1.45)	1.26 (0.98-1.61)<br>1.24 (1-1.54)	1.81 (1.22-2.68)<br>1.31 (0.94-1.84)
**Asthma**					
BMI 18.5-24.9 kg/m²<br>BMI 25-29.9 kg/m²	1 (Reference)<br>1.26 (1.16-1.37)	1.02 (0.94-1.1)<br>1.29 (1.2-1.39)	1.05 (0.97-1.13)<br>1.29 (1.2-1.39)	1.17 (1.06-1.3)<br>1.37 (1.26-1.49)	1.53 (1.3-1.82)<br>1.78 (1.55-2.05)
**Liver disease**					
BMI 18.5-24.9 kg/m²<br>BMI 25-29.9 kg/m²	1 (Reference)<br>1.53 (1.25-1.89)	1.19 (0.98-1.45)<br>1.91 (1.59-2.29)	1.6 (1.32-1.95)<br>2.11 (1.76-2.53)	2.46 (1.99-3.03)<br>2.49 (2.06-3.02)	3.05 (2.23-4.18)<br>3.22 (2.51-4.15)
**Renal failure**					
BMI 18.5-24.9 kg/m²<br>BMI 25-29.9 kg/m²	1 (Reference)<br>1.37 (1.21-1.54)	0.99 (0.88-1.12)<br>1.43 (1.28-1.59)	1.23 (1.09-1.38)<br>1.61 (1.45-1.79)	1.62 (1.41-1.85)<br>1.87 (1.67-2.1)	1.88 (1.51-2.36)<br>2.09 (1.78-2.46)
**Gout**					
BMI 18.5-24.9 kg/m²<br>BMI 25-29.9 kg/m²	1 (Reference)<br>2.42 (1.56-3.76)	1.30 (0.82-2.05)<br>3.23 (2.16-4.83)	1.74 (1.11-2.72)<br>4.10 (2.75-6.11)	2.13 (1.3-3.49)<br>5.23 (3.49-7.85)	3.40 (1.75-6.62)<br>7.40 (4.71-11.61)
**Osteoarthritis**					
BMI 18.5-24.9 kg/m²<br>BMI 25-29.9 kg/m²	1 (Reference)<br>1.60 (1.49-1.71)	1.1 (1.02-1.17)<br>1.75 (1.64-1.86)	1.16 (1.08-1.24)<br>1.81 (1.7-1.93)	1.28 (1.18-1.4)<br>1.89 (1.76-2.02)	1.15 (0.97-1.36)<br>1.84 (1.66-2.05)
**Sleep disorders**					
BMI 18.5-24.9 kg/m²<br>BMI 25-29.9 kg/m²	1 (Reference)<br>1.69 (1.3-2.2)	1.06 (0.81-1.38)<br>1.80 (1.42-2.29)	1.05 (0.8-1.37)<br>2.06 (1.63-2.61)	1.41 (1.04-1.91)<br>2.42 (1.89-3.1)	1.43 (0.84-2.45)<br>2.48 (1.76-3.48)
**Mood disorders**					
BMI 18.5-24.9 kg/m²<br>BMI 25-29.9 kg/m²	1 (Reference)<br>1.40 (1.25-1.58)	1.24 (1.12-1.39)<br>1.58 (1.43-1.75)	1.49 (1.34-1.65)<br>1.82 (1.65-2.02)	1.98 (1.76-2.24)<br>2.06 (1.85-2.31)	2.5 (2.06-3.03)<br>2.49 (2.12-2.92)

[a] HR adjusted for age, sex, and Townsend deprivation index, Healthy lifestyle score included 4 lifestyle factors (never smoking, no or moderate alcohol consumption, physical activity, healthy diet). Participants scored 1 if they met the criterion for a healthy lifestyle and 0 otherwise

© 2023 Rassy N et al. *JAMA Network Open*.

eTable 8. Associations of Body Mass Index With All-Cause Mortality by Healthy Lifestyle Score

	Healthy lifestyle score Adjusted HR (95% CI)[a]				
**All-cause mortality**	**4**	**3**	**2**	**1**	**0**
BMI 18.5-24.9 kg/m²	1 (Reference)	1.28 (1.14-1.43)	1.63 (1.46-1.82)	2.27 (2.01-2.56)	3.16 (2.65-3.77)
BMI 25-29.9 kg/m²	1.03 (0.90-1.16)	1.21 (1.09-1.35)	1.47 (1.32-1.63)	1.85 (1.65-2.06)	2.25 (1.93-2.62)
BMI ≥ 30 kg/m²	1.37 (1.19-1.57)	1.57 (1.40-1.76)	1.99 (1.78-2.21)	2.21 (1.97-2.48)	2.43 (2.06-2.86)

HR, hazard ratio; CI, confidence interval , BMI, Body mass index

[a] HR adjusted for age, sex, and Townsend deprivation index

Healthy lifestyle score included 4 lifestyle factors (never smoking, no or moderate alcohol consumption, physical activity, healthy diet). Participants scored 1 if they met the criterion for a healthy lifestyle and 0 otherwise

© 2023 Rassy N et al. *JAMA Network Open*.

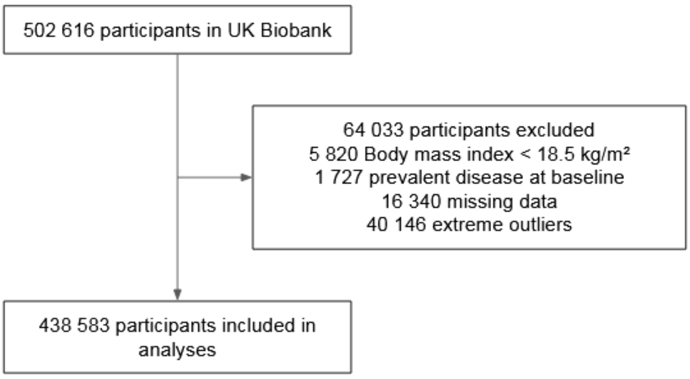

eFigure 1. Flowchart of Sample Selection

© 2023 Rassy N et al. *JAMA Network Open*.

eFigure 2. Hazard Ratios for Incident Diseases by Body Mass Index Category

BMI, body mass index; HR, hazard ratio; CI, confidence interval
Hazard ratios (95% CI) for obesity (BMI ≥30.0 kg/m²) and overweight (25-29.9 kg/m²) versus healthy weight (18.5–24.9 kg/m²) were calculated in Cox proportional hazards models. All the p-values are presented after Bonferroni multiple testing adjustments.
Healthy lifestyle score included 4 lifestyle factors (never smoking, no or moderate alcohol consumption, physical activity, healthy diet). Individuals scored 1 if they met the criterion for a healthy lifestyle and 0 otherwise

Disease	BMI	N (%) Cases	HR (95% CI)	Corrected pvalue
Infections	18.5 ≤ BMI < 25	5051 / 144579 (3.49%)	-	
	25 ≤ BMI < 30	7312 / 186962 (3.91%)	1.12 (1.08-1.16)	< 0.001
	BMI ≥ 30	5569 / 107041 (5.2%)	1.51 (1.45-1.57)	< 0.001
Diabete	18.5 ≤ BMI < 25	2061 / 144579 (1.43%)	-	
	25 ≤ BMI < 30	6850 / 186962 (3.66%)	2.6 (2.48-2.73)	< 0.001
	BMI ≥ 30	11500 / 107041 (10.74%)	7.94 (7.58-8.32)	< 0.001
Esophageal Adenocarcinoma	18.5 ≤ BMI < 25	164 / 144579 (0.11%)	-	
	25 ≤ BMI < 30	329 / 186962 (0.18%)	1.55 (1.29-1.87)	< 0.001
	BMI ≥ 30	233 / 107041 (0.22%)	1.93 (1.58-2.36)	< 0.001
Gastric Cardia Cancer	18.5 ≤ BMI < 25	60 / 144579 (0.04%)	-	
	25 ≤ BMI < 30	132 / 186962 (0.07%)	1.7 (1.26-2.31)	0.001
	BMI ≥ 30	115 / 107041 (0.11%)	2.6 (1.91-3.56)	< 0.001
Pancreatic Cancer	18.5 ≤ BMI < 25	219 / 144579 (0.15%)	-	
	25 ≤ BMI < 30	345 / 186962 (0.18%)	1.22 (1.03-1.45)	0.04
	BMI ≥ 30	226 / 107041 (0.21%)	1.4 (1.16-1.69)	< 0.001
Liver Cancer	18.5 ≤ BMI < 25	114 / 144579 (0.08%)	-	
	25 ≤ BMI < 30	172 / 186962 (0.09%)	1.17 (0.92-1.48)	0.39
	BMI ≥ 30	165 / 107041 (0.15%)	1.97 (1.55-2.5)	< 0.001
Colorectal Cancer	18.5 ≤ BMI < 25	1008 / 144579 (0.7%)	-	
	25 ≤ BMI < 30	1758 / 186962 (0.94%)	1.35 (1.25-1.46)	< 0.001
	BMI ≥ 30	1027 / 107041 (0.96%)	1.39 (1.27-1.51)	< 0.001
Breast Cancer	18.5 ≤ BMI < 25	2436 / 144579 (1.68%)	-	
	25 ≤ BMI < 30	2566 / 186962 (1.37%)	0.81 (0.77-0.86)	< 0.001
	BMI ≥ 30	1757 / 107041 (1.64%)	0.98 (0.92-1.04)	1
Ovarian Cancer	18.5 ≤ BMI < 25	265 / 144579 (0.18%)	-	
	25 ≤ BMI < 30	303 / 186962 (0.16%)	0.89 (0.75-1.04)	0.30
	BMI ≥ 30	195 / 107041 (0.18%)	1 (0.83-1.2)	1
Endometrial Cancer	18.5 ≤ BMI < 25	216 / 144579 (0.15%)	-	
	25 ≤ BMI < 30	320 / 186962 (0.17%)	1.15 (0.97-1.36)	0.24
	BMI ≥ 30	426 / 107041 (0.4%)	2.68 (2.28-3.16)	< 0.001
Kidney Cancer	18.5 ≤ BMI < 25	178 / 144579 (0.12%)	-	
	25 ≤ BMI < 30	426 / 186962 (0.23%)	1.85 (1.56-2.21)	< 0.001
	BMI ≥ 30	329 / 107041 (0.31%)	2.51 (2.09-3.02)	< 0.001
Hypertension	18.5 ≤ BMI < 25	12778 / 144579 (8.84%)	-	
	25 ≤ BMI < 30	28693 / 186962 (15.35%)	1.8 (1.77-1.84)	< 0.001
	BMI ≥ 30	24831 / 107041 (23.2%)	2.88 (2.82-2.94)	< 0.001
Ischemic Heart Disease	18.5 ≤ BMI < 25	5521 / 144579 (3.82%)	-	
	25 ≤ BMI < 30	12615 / 186962 (6.75%)	1.8 (1.74-1.85)	< 0.001
	BMI ≥ 30	10489 / 107041 (9.8%)	2.66 (2.58-2.75)	< 0.001
Pulmonary Embolism	18.5 ≤ BMI < 25	654 / 144579 (0.45%)	-	
	25 ≤ BMI < 30	1382 / 186962 (0.74%)	1.64 (1.49-1.8)	< 0.001
	BMI ≥ 30	1222 / 107041 (1.14%)	2.55 (2.32-2.8)	< 0.001
Arrhythmias	18.5 ≤ BMI < 25	4257 / 144579 (2.94%)	-	
	25 ≤ BMI < 30	7481 / 186962 (4%)	1.37 (1.32-1.42)	< 0.001
	BMI ≥ 30	6259 / 107041 (5.85%)	2.03 (1.95-2.11)	< 0.001
Heart Failure	18.5 ≤ BMI < 25	1136 / 144579 (0.79%)	-	
	25 ≤ BMI < 30	2308 / 186962 (1.23%)	1.58 (1.47-1.69)	< 0.001
	BMI ≥ 30	2693 / 107041 (2.52%)	3.25 (3.03-3.48)	< 0.001
Cerebrovascular Diseases	18.5 ≤ BMI < 25	2037 / 144579 (1.41%)	-	
	25 ≤ BMI < 30	3432 / 186962 (1.84%)	1.31 (1.24-1.38)	< 0.001
	BMI ≥ 30	2532 / 107041 (2.37%)	1.7 (1.6-1.8)	< 0.001
Arteriosclerosis	18.5 ≤ BMI < 25	110 / 144579 (0.08%)	-	
	25 ≤ BMI < 30	198 / 186962 (0.11%)	1.39 (1.1-1.76)	0.01
	BMI ≥ 30	148 / 107041 (0.14%)	1.82 (1.42-2.33)	< 0.001
Deep Vein Thrombosis	18.5 ≤ BMI < 25	731 / 144579 (0.51%)	-	
	25 ≤ BMI < 30	1303 / 186962 (0.7%)	1.38 (1.26-1.51)	< 0.001
	BMI ≥ 30	1130 / 107041 (1.06%)	2.11 (1.92-2.31)	< 0.001
Asthma	18.5 ≤ BMI < 25	5082 / 144579 (3.52%)	-	
	25 ≤ BMI < 30	7914 / 186962 (4.23%)	1.21 (1.17-1.25)	< 0.001
	BMI ≥ 30	6418 / 107041 (6%)	1.74 (1.68-1.8)	< 0.001
Liver Disease	18.5 ≤ BMI < 25	1094 / 144579 (0.76%)	-	
	25 ≤ BMI < 30	2114 / 186962 (1.13%)	1.5 (1.39-1.61)	< 0.001
	BMI ≥ 30	2305 / 107041 (2.15%)	2.88 (2.68-3.1)	< 0.001
Renal Failure	18.5 ≤ BMI < 25	2485 / 144579 (1.72%)	-	
	25 ≤ BMI < 30	5147 / 186962 (2.75%)	1.61 (1.54-1.69)	< 0.001
	BMI ≥ 30	5395 / 107041 (5.04%)	3 (2.86-3.14)	< 0.001
Gout	18.5 ≤ BMI < 25	213 / 144579 (0.15%)	-	
	25 ≤ BMI < 30	990 / 186962 (0.53%)	3.61 (3.11-4.18)	< 0.001
	BMI ≥ 30	1246 / 107041 (1.16%)	8 (6.92-9.25)	< 0.001
Osteoarthritis	18.5 ≤ BMI < 25	7112 / 144579 (4.92%)	-	
	25 ≤ BMI < 30	14853 / 186962 (7.94%)	1.64 (1.6-1.69)	< 0.001
	BMI ≥ 30	14700 / 107041 (13.73%)	2.95 (2.87-3.04)	< 0.001
Sleep Disorders	18.5 ≤ BMI < 25	479 / 144579 (0.33%)	-	
	25 ≤ BMI < 30	1294 / 186962 (0.69%)	2.1 (1.89-2.33)	< 0.001
	BMI ≥ 30	2698 / 107041 (2.52%)	7.74 (7.02-8.53)	< 0.001
Mood Disorders	18.5 ≤ BMI < 25	3413 / 144579 (2.36%)	-	
	25 ≤ BMI < 30	5116 / 186962 (2.74%)	1.16 (1.11-1.21)	< 0.001
	BMI ≥ 30	4846 / 107041 (4.53%)	1.95 (1.87-2.04)	< 0.001

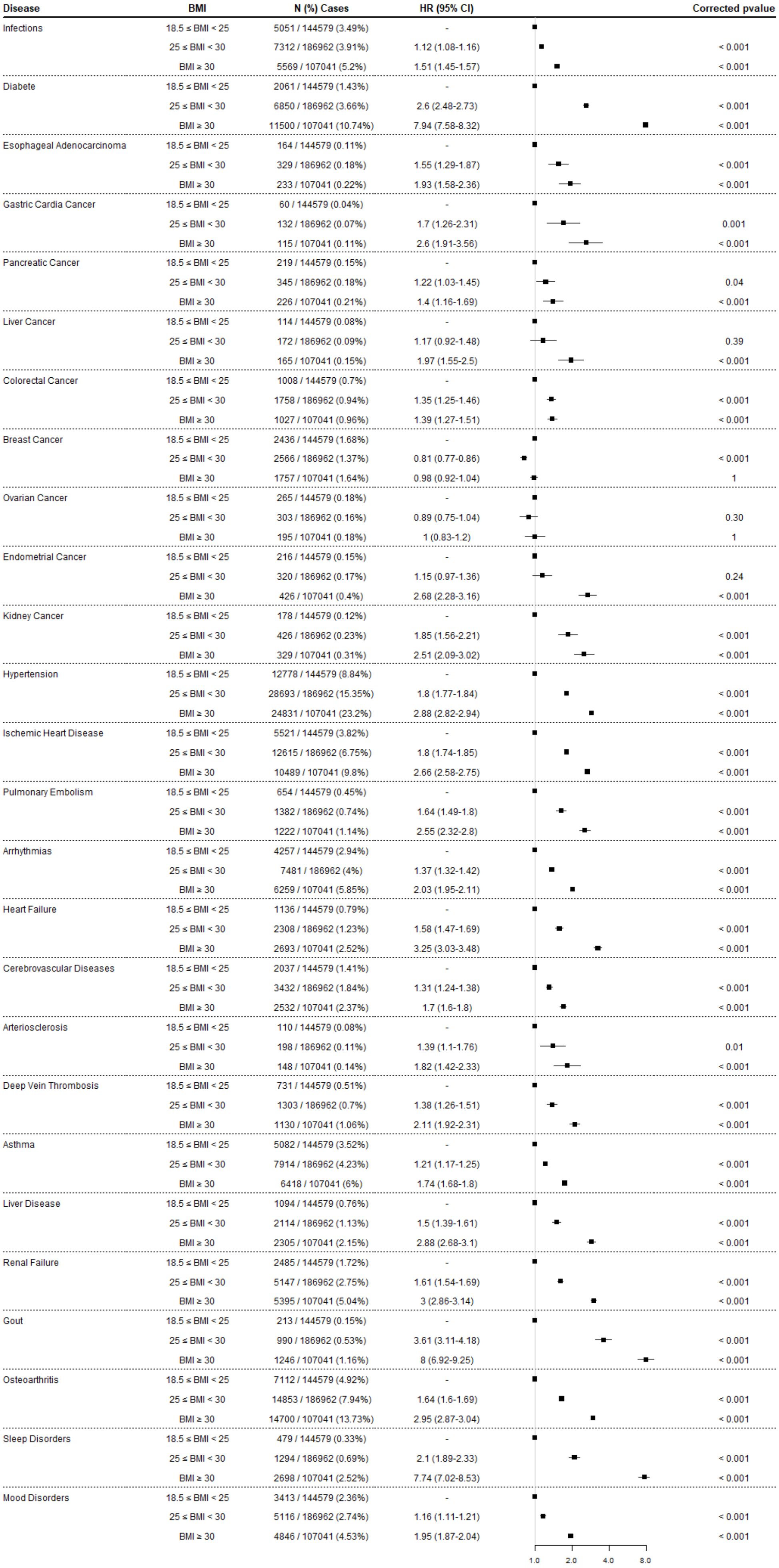

© 2023 Rassy N et al. *JAMA Network Open*.

eFigure 3. Adjusted Hazard Ratios for Incident Diseases by Body Mass Index Category

BMI, body mass index; HR, hazard ratio; CI, confidence interval

Hazard ratios (95% CI) for obesity (BMI ≥30.0 kg/m²) and overweight (25-29.9 kg/m²) versus healthy weight (18.5–24.9 kg/m²) were calculated in Cox proportional hazards models after adjustment of age, sex, Townsend deprivation index, and healthy lifestyle score. All the p-values are presented after Bonferroni multiple testing adjustments.

Healthy lifestyle score included 4 lifestyle factors (never smoking, no or moderate alcohol consumption, physical activity, healthy diet). Individuals scored 1 if they met the criterion for a healthy lifestyle and 0 otherwise

Disease	BMI	N (%) Cases	HR (95% CI)	Corrected pvalue
Infections	18.5 ≤ BMI < 25	5051 / 144579 (3.49%)	-	
	25 ≤ BMI < 30	7312 / 186962 (3.91%)	1.05 (1.02-1.09)	0.05
	BMI ≥ 30	5569 / 107041 (5.2%)	1.36 (1.31-1.42)	< 0.001
Diabete	18.5 ≤ BMI < 25	2061 / 144579 (1.43%)	-	
	25 ≤ BMI < 30	6850 / 186962 (3.66%)	2.21 (2.1-2.32)	< 0.001
	BMI ≥ 30	11500 / 107041 (10.74%)	6.66 (6.36-6.98)	< 0.001
Esophageal Adenocarcinoma	18.5 ≤ BMI < 25	164 / 144579 (0.11%)	-	
	25 ≤ BMI < 30	329 / 186962 (0.18%)	1.1 (0.91-1.33)	1
	BMI ≥ 30	233 / 107041 (0.22%)	1.37 (1.12-1.67)	0.02
Gastric Cardia Cancer	18.5 ≤ BMI < 25	60 / 144579 (0.04%)	-	
	25 ≤ BMI < 30	132 / 186962 (0.07%)	1.16 (0.85-1.58)	1
	BMI ≥ 30	115 / 107041 (0.11%)	1.89 (1.38-2.59)	< 0.001
Pancreatic Cancer	18.5 ≤ BMI < 25	219 / 144579 (0.15%)	-	
	25 ≤ BMI < 30	345 / 186962 (0.18%)	1.05 (0.88-1.24)	1
	BMI ≥ 30	226 / 107041 (0.21%)	1.21 (1-1.46)	0.42
Liver Cancer	18.5 ≤ BMI < 25	114 / 144579 (0.08%)	-	
	25 ≤ BMI < 30	172 / 186962 (0.09%)	0.95 (0.75-1.2)	1
	BMI ≥ 30	165 / 107041 (0.15%)	1.58 (1.24-2.02)	0.002
Colorectal Cancer	18.5 ≤ BMI < 25	1008 / 144579 (0.7%)	-	
	25 ≤ BMI < 30	1758 / 186962 (0.94%)	1.13 (1.05-1.23)	0.01
	BMI ≥ 30	1027 / 107041 (0.96%)	1.19 (1.09-1.3)	0.001
Breast Cancer	18.5 ≤ BMI < 25	2436 / 144579 (1.68%)	-	
	25 ≤ BMI < 30	2566 / 186962 (1.37%)	1.07 (1.01-1.13)	0.17
	BMI ≥ 30	1757 / 107041 (1.64%)	1.17 (1.1-1.24)	< 0.001
Ovarian Cancer	18.5 ≤ BMI < 25	265 / 144579 (0.18%)	-	
	25 ≤ BMI < 30	303 / 186962 (0.16%)	1.12 (0.95-1.32)	1
	BMI ≥ 30	195 / 107041 (0.18%)	1.15 (0.95-1.38)	1
Endometrial Cancer	18.5 ≤ BMI < 25	216 / 144579 (0.15%)	-	
	25 ≤ BMI < 30	320 / 186962 (0.17%)	1.46 (1.23-1.73)	< 0.001
	BMI ≥ 30	426 / 107041 (0.4%)	3.13 (2.66-3.7)	< 0.001
Kidney Cancer	18.5 ≤ BMI < 25	178 / 144579 (0.12%)	-	
	25 ≤ BMI < 30	426 / 186962 (0.23%)	1.51 (1.26-1.8)	< 0.001
	BMI ≥ 30	329 / 107041 (0.31%)	2.11 (1.76-2.54)	< 0.001
Hypertension	18.5 ≤ BMI < 25	12778 / 144579 (8.84%)	-	
	25 ≤ BMI < 30	28693 / 186962 (15.35%)	1.58 (1.55-1.61)	< 0.001
	BMI ≥ 30	24831 / 107041 (23.2%)	2.53 (2.48-2.59)	< 0.001
Ischemic Heart Disease	18.5 ≤ BMI < 25	5521 / 144579 (3.82%)	-	
	25 ≤ BMI < 30	12615 / 186962 (6.75%)	1.39 (1.35-1.44)	< 0.001
	BMI ≥ 30	10489 / 107041 (9.8%)	2.12 (2.06-2.2)	< 0.001
Pulmonary Embolism	18.5 ≤ BMI < 25	654 / 144579 (0.45%)	-	
	25 ≤ BMI < 30	1382 / 186962 (0.74%)	1.43 (1.3-1.57)	< 0.001
	BMI ≥ 30	1222 / 107041 (1.14%)	2.23 (2.02-2.45)	< 0.001
Arrhythmias	18.5 ≤ BMI < 25	4257 / 144579 (2.94%)	-	
	25 ≤ BMI < 30	7481 / 186962 (4%)	1.09 (1.05-1.13)	< 0.001
	BMI ≥ 30	6259 / 107041 (5.85%)	1.68 (1.61-1.75)	< 0.001
Heart Failure	18.5 ≤ BMI < 25	1136 / 144579 (0.79%)	-	
	25 ≤ BMI < 30	2308 / 186962 (1.23%)	1.2 (1.11-1.29)	< 0.001
	BMI ≥ 30	2693 / 107041 (2.52%)	2.47 (2.3-2.65)	< 0.001
Cerebrovascular Diseases	18.5 ≤ BMI < 25	2037 / 144579 (1.41%)	-	
	25 ≤ BMI < 30	3432 / 186962 (1.84%)	1.06 (1-1.12)	0.31
	BMI ≥ 30	2532 / 107041 (2.37%)	1.36 (1.28-1.44)	< 0.001
Arteriosclerosis	18.5 ≤ BMI < 25	110 / 144579 (0.08%)	-	
	25 ≤ BMI < 30	198 / 186962 (0.11%)	1 (0.79-1.26)	1
	BMI ≥ 30	148 / 107041 (0.14%)	1.22 (0.95-1.57)	1
Deep Vein Thrombosis	18.5 ≤ BMI < 25	731 / 144579 (0.51%)	-	
	25 ≤ BMI < 30	1303 / 186962 (0.7%)	1.21 (1.11-1.33)	< 0.001
	BMI ≥ 30	1130 / 107041 (1.06%)	1.86 (1.69-2.04)	< 0.001
Asthma	18.5 ≤ BMI < 25	5082 / 144579 (3.52%)	-	
	25 ≤ BMI < 30	7914 / 186962 (4.23%)	1.23 (1.19-1.27)	< 0.001
	BMI ≥ 30	6418 / 107041 (6%)	1.72 (1.65-1.78)	< 0.001
Liver Disease	18.5 ≤ BMI < 25	1094 / 144579 (0.76%)	-	
	25 ≤ BMI < 30	2114 / 186962 (1.13%)	1.35 (1.25-1.45)	< 0.001
	BMI ≥ 30	2305 / 107041 (2.15%)	2.45 (2.28-2.64)	< 0.001
Renal Failure	18.5 ≤ BMI < 25	2485 / 144579 (1.72%)	-	
	25 ≤ BMI < 30	5147 / 186962 (2.75%)	1.32 (1.26-1.39)	< 0.001
	BMI ≥ 30	5395 / 107041 (5.04%)	2.43 (2.32-2.55)	< 0.001
Gout	18.5 ≤ BMI < 25	213 / 144579 (0.15%)	-	
	25 ≤ BMI < 30	990 / 186962 (0.53%)	2.42 (2.09-2.81)	< 0.001
	BMI ≥ 30	1246 / 107041 (1.16%)	5.6 (4.84-6.48)	< 0.001
Osteoarthritis	18.5 ≤ BMI < 25	7112 / 144579 (4.92%)	-	
	25 ≤ BMI < 30	14853 / 186962 (7.94%)	1.57 (1.53-1.62)	< 0.001
	BMI ≥ 30	14700 / 107041 (13.73%)	2.81 (2.73-2.89)	< 0.001
Sleep Disorders	18.5 ≤ BMI < 25	479 / 144579 (0.33%)	-	
	25 ≤ BMI < 30	1294 / 186962 (0.69%)	1.78 (1.6-1.98)	< 0.001
	BMI ≥ 30	2698 / 107041 (2.52%)	6.51 (5.91-7.18)	< 0.001
Mood Disorders	18.5 ≤ BMI < 25	3413 / 144579 (2.36%)	-	
	25 ≤ BMI < 30	5116 / 186962 (2.74%)	1.22 (1.17-1.28)	< 0.001
	BMI ≥ 30	4846 / 107041 (4.53%)	1.88 (1.8-1.97)	< 0.001

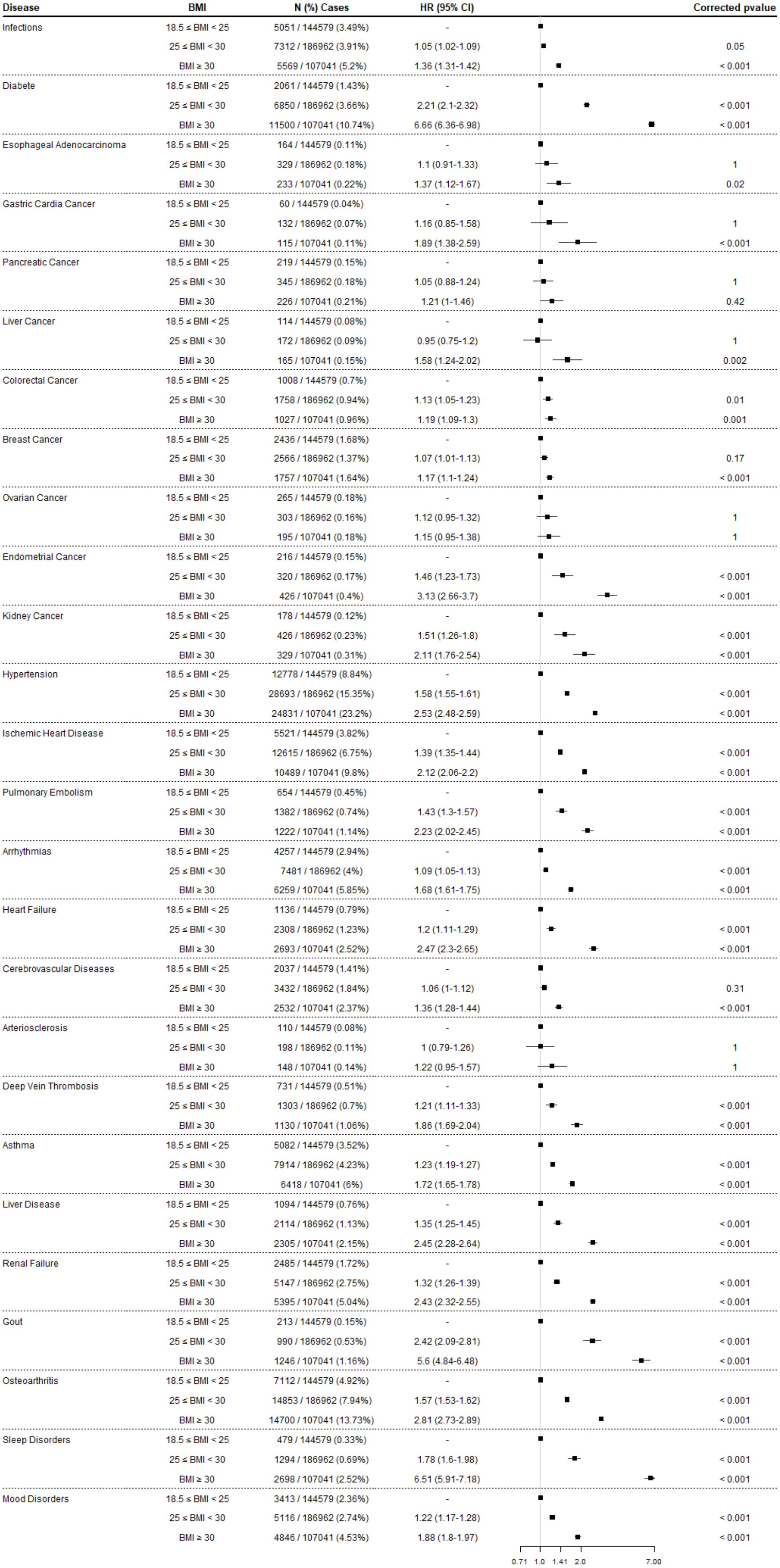

© 2023 Rassy N et al. *JAMA Network Open*.

eFigure 4 A. Hazard Ratios for Incident Diseases by Lifestyle Profile in Individuals With Normal Weight (BMI 18.5-24.9)

BMI, body mass index; HR, hazard ratio; CI, confidence interval

Hazard ratios (95% CI) for a lifestyle score equal to 1, 2, 3 or 4 versus a lifestyle score equal to zero were calculated in Cox proportional hazards models. All the p-values are presented after Bonferroni multiple testing adjustments.

For each lifestyle factor, individuals scored 1 if they met the criterion for a healthy lifestyle (never smoking, no or moderate alcohol consumption, physical activity, or healthy diet) and 0 otherwise. The healthy lifestyle score was computed by summing the four scores. It ranged between zero (lowest healthy score) and 4 (highest healthy score).

Disease	Healthy lifestyle factors	N (%) Cases	HR (95% CI)	Corrected pvalue
Infections	Zero	169 / 3190 (5.3%)	-	
	One	794 / 17689 (4.49%)	0.83 (0.71-0.99)	0.13
	Two	1617 / 44001 (3.67%)	0.68 (0.58-0.79)	< 0.001
	Three	1652 / 51199 (3.23%)	0.59 (0.5-0.69)	< 0.001
	Four	819 / 28500 (2.87%)	0.52 (0.44-0.62)	< 0.001
Diabete	Zero	67 / 3190 (2.1%)	-	
	One	281 / 17689 (1.59%)	0.75 (0.57-0.97)	0.13
	Two	633 / 44001 (1.44%)	0.67 (0.52-0.86)	0.008
	Three	732 / 51199 (1.43%)	0.67 (0.52-0.86)	0.006
	Four	348 / 28500 (1.22%)	0.57 (0.44-0.74)	< 0.001
Esophageal Adenocarcinoma	Zero	11 / 3190 (0.34%)	-	
	One	33 / 17689 (0.19%)	0.54 (0.27-1.06)	0.29
	Two	61 / 44001 (0.14%)	0.4 (0.21-0.75)	0.02
	Three	41 / 51199 (0.08%)	0.23 (0.12-0.44)	< 0.001
	Four	18 / 28500 (0.06%)	0.18 (0.08-0.38)	< 0.001
Gastric Cardia Cancer	Zero	5 / 3190 (0.16%)	-	
	One	11 / 17689 (0.06%)	0.39 (0.14-1.13)	0.33
	Two	18 / 44001 (0.04%)	0.26 (0.1-0.69)	0.03
	Three	19 / 51199 (0.04%)	0.23 (0.09-0.62)	0.01
	Four	7 / 28500 (0.02%)	0.15 (0.05-0.48)	0.005
Pancreatic Cancer	Zero	6 / 3190 (0.19%)	-	
	One	40 / 17689 (0.23%)	1.19 (0.5-2.81)	1
	Two	71 / 44001 (0.16%)	0.84 (0.37-1.94)	1
	Three	63 / 51199 (0.12%)	0.64 (0.28-1.48)	1
	Four	39 / 28500 (0.14%)	0.71 (0.3-1.68)	1
Liver Cancer	Zero	6 / 3190 (0.19%)	-	
	One	21 / 17689 (0.12%)	0.62 (0.25-1.55)	1
	Two	34 / 44001 (0.08%)	0.4 (0.17-0.96)	0.16
	Three	40 / 51199 (0.08%)	0.41 (0.17-0.96)	0.16
	Four	13 / 28500 (0.05%)	0.24 (0.09-0.62)	0.01
Colorectal Cancer	Zero	22 / 3190 (0.69%)	-	
	One	148 / 17689 (0.84%)	1.2 (0.77-1.88)	1
	Two	320 / 44001 (0.73%)	1.04 (0.67-1.6)	1
	Three	335 / 51199 (0.65%)	0.93 (0.6-1.43)	1
	Four	183 / 28500 (0.64%)	0.91 (0.59-1.42)	1
Breast Cancer	Zero	43 / 3190 (1.35%)	-	
	One	302 / 17689 (1.71%)	1.26 (0.91-1.73)	0.64
	Two	771 / 44001 (1.75%)	1.28 (0.94-1.74)	0.45
	Three	871 / 51199 (1.7%)	1.24 (0.91-1.69)	0.66
	Four	449 / 28500 (1.58%)	1.15 (0.84-1.57)	1
Ovarian Cancer	Zero	2 / 3190 (0.06%)	-	
	One	25 / 17689 (0.14%)	2.23 (0.53-9.43)	1
	Two	76 / 44001 (0.17%)	2.71 (0.67-11.05)	0.65
	Three	99 / 51199 (0.19%)	3.03 (0.75-12.29)	0.48
	Four	63 / 28500 (0.22%)	3.46 (0.85-14.13)	0.34
Endometrial Cancer	Zero	0 / 3190 (0%)	-	
	One	23 / 17689 (0.13%)	2952470.06 (0-Inf)	1
	Two	52 / 44001 (0.12%)	2665362.57 (0-Inf)	1
	Three	88 / 51199 (0.17%)	3866486.14 (0-Inf)	1
	Four	53 / 28500 (0.19%)	4174347.09 (0-Inf)	1
Kidney Cancer	Zero	8 / 3190 (0.25%)	-	
	One	27 / 17689 (0.15%)	0.6 (0.27-1.33)	0.83
	Two	49 / 44001 (0.11%)	0.44 (0.21-0.92)	0.12
	Three	66 / 51199 (0.13%)	0.5 (0.24-1.05)	0.27
	Four	28 / 28500 (0.1%)	0.38 (0.17-0.84)	0.07
Hypertension	Zero	436 / 3190 (13.67%)	-	
	One	1961 / 17689 (11.09%)	0.79 (0.71-0.88)	< 0.001
	Two	3966 / 44001 (9.01%)	0.63 (0.57-0.7)	< 0.001
	Three	4134 / 51199 (8.07%)	0.56 (0.51-0.62)	< 0.001
	Four	2281 / 28500 (8%)	0.56 (0.5-0.62)	< 0.001
Ischemic Heart Disease	Zero	219 / 3190 (6.87%)	-	
	One	930 / 17689 (5.26%)	0.75 (0.65-0.87)	< 0.001
	Two	1720 / 44001 (3.91%)	0.55 (0.48-0.64)	< 0.001
	Three	1744 / 51199 (3.41%)	0.48 (0.42-0.55)	< 0.001
	Four	908 / 28500 (3.19%)	0.45 (0.39-0.52)	< 0.001
Pulmonary Embolism	Zero	31 / 3190 (0.97%)	-	
	One	116 / 17689 (0.66%)	0.67 (0.45-0.99)	0.18
	Two	193 / 44001 (0.44%)	0.44 (0.3-0.65)	< 0.001
	Three	204 / 51199 (0.4%)	0.4 (0.27-0.58)	< 0.001
	Four	110 / 28500 (0.39%)	0.39 (0.26-0.58)	< 0.001
Arrhythmias	Zero	134 / 3190 (4.2%)	-	
	One	619 / 17689 (3.5%)	0.82 (0.68-0.99)	0.17
	Two	1317 / 44001 (2.99%)	0.7 (0.58-0.83)	< 0.001
	Three	1387 / 51199 (2.71%)	0.63 (0.53-0.75)	< 0.001
	Four	800 / 28500 (2.81%)	0.65 (0.54-0.78)	< 0.001
Heart Failure	Zero	52 / 3190 (1.63%)	-	
	One	209 / 17689 (1.18%)	0.72 (0.53-0.97)	0.12
	Two	364 / 44001 (0.83%)	0.5 (0.37-0.66)	< 0.001
	Three	334 / 51199 (0.65%)	0.39 (0.29-0.52)	< 0.001
	Four	177 / 28500 (0.62%)	0.37 (0.27-0.5)	< 0.001
Cerebrovascular Diseases	Zero	85 / 3190 (2.66%)	-	
	One	349 / 17689 (1.97%)	0.73 (0.58-0.93)	0.04
	Two	650 / 44001 (1.48%)	0.54 (0.43-0.68)	< 0.001
	Three	597 / 51199 (1.17%)	0.43 (0.34-0.53)	< 0.001
	Four	356 / 28500 (1.25%)	0.46 (0.36-0.58)	< 0.001
Arteriosclerosis	Zero	12 / 3190 (0.38%)	-	
	One	33 / 17689 (0.19%)	0.49 (0.25-0.95)	0.14
	Two	36 / 44001 (0.08%)	0.22 (0.11-0.41)	< 0.001
	Three	19 / 51199 (0.04%)	0.1 (0.05-0.2)	< 0.001
	Four	10 / 28500 (0.04%)	0.09 (0.04-0.21)	< 0.001
Deep Vein Thrombosis	Zero	30 / 3190 (0.94%)	-	
	One	114 / 17689 (0.64%)	0.68 (0.45-1.01)	0.23
	Two	227 / 44001 (0.52%)	0.54 (0.37-0.79)	0.006
	Three	217 / 51199 (0.42%)	0.44 (0.3-0.65)	< 0.001
	Four	143 / 28500 (0.5%)	0.52 (0.35-0.77)	0.005
Asthma	Zero	156 / 3190 (4.89%)	-	
	One	682 / 17689 (3.86%)	0.78 (0.65-0.92)	0.02
	Two	1540 / 44001 (3.5%)	0.7 (0.59-0.82)	< 0.001
	Three	1745 / 51199 (3.41%)	0.68 (0.58-0.8)	< 0.001
	Four	959 / 28500 (3.36%)	0.67 (0.56-0.79)	< 0.001
Liver Disease	Zero	53 / 3190 (1.66%)	-	
	One	230 / 17689 (1.3%)	0.77 (0.57-1.04)	0.36
	Two	361 / 44001 (0.82%)	0.48 (0.36-0.65)	< 0.001
	Three	307 / 51199 (0.6%)	0.35 (0.26-0.47)	< 0.001
	Four	143 / 28500 (0.5%)	0.29 (0.21-0.4)	< 0.001
Renal Failure	Zero	93 / 3190 (2.92%)	-	
	One	436 / 17689 (2.46%)	0.83 (0.67-1.04)	0.45
	Two	794 / 44001 (1.8%)	0.6 (0.49-0.75)	< 0.001
	Three	740 / 51199 (1.45%)	0.48 (0.39-0.6)	< 0.001
	Four	422 / 28500 (1.48%)	0.49 (0.39-0.62)	< 0.001
Gout	Zero	13 / 3190 (0.41%)	-	
	One	41 / 17689 (0.23%)	0.56 (0.3-1.05)	0.28
	Two	72 / 44001 (0.16%)	0.39 (0.22-0.71)	0.008
	Three	61 / 51199 (0.12%)	0.28 (0.16-0.52)	< 0.001
	Four	26 / 28500 (0.09%)	0.22 (0.11-0.42)	< 0.001
Osteoarthritis	Zero	149 / 3190 (4.67%)	-	
	One	964 / 17689 (5.45%)	1.16 (0.98-1.38)	0.38
	Two	2224 / 44001 (5.05%)	1.07 (0.9-1.26)	1
	Three	2480 / 51199 (4.84%)	1.02 (0.86-1.2)	1
	Four	1295 / 28500 (4.54%)	0.95 (0.8-1.13)	1
Sleep Disorders	Zero	16 / 3190 (0.5%)	-	
	One	81 / 17689 (0.46%)	0.9 (0.53-1.55)	1
	Two	139 / 44001 (0.32%)	0.62 (0.37-1.04)	0.28
	Three	160 / 51199 (0.31%)	0.61 (0.37-1.02)	0.24
	Four	83 / 28500 (0.29%)	0.57 (0.33-0.97)	0.15
Mood Disorders	Zero	131 / 3190 (4.11%)	-	
	One	592 / 17689 (3.35%)	0.8 (0.66-0.97)	0.09
	Two	1121 / 44001 (2.55%)	0.6 (0.5-0.72)	< 0.001
	Three	1085 / 51199 (2.12%)	0.5 (0.42-0.6)	< 0.001
	Four	484 / 28500 (1.7%)	0.4 (0.33-0.48)	< 0.001

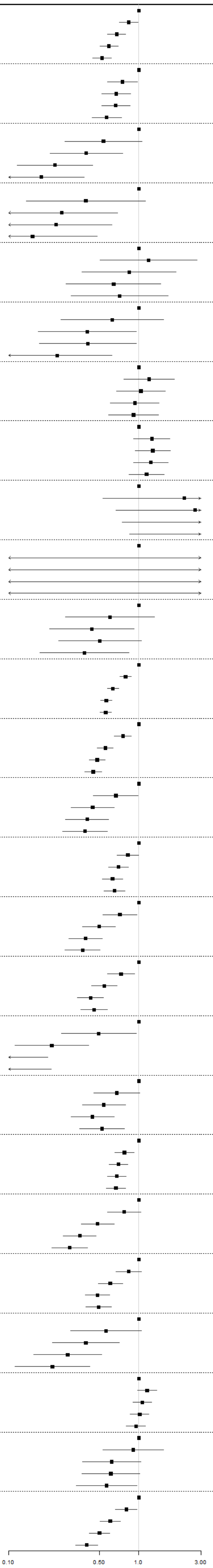

© 2023 Rassy N et al. *JAMA Network Open*.

eFigure 4 B. Hazard Ratios for Incident Diseases by Lifestyle Profile in Individuals With Overweight (BMI 25-29.9)

Disease	Healthy lifestyle factors	N (%) Cases	HR (95% CI)	Corrected pvalue
Infections	Zero	278 / 5857 (4.75%)	-	
	One	1212 / 28851 (4.2%)	0.88 (0.77-1)	0.21
	Two	2461 / 60934 (4.04%)	0.84 (0.74-0.95)	0.02
	Three	2301 / 61760 (3.73%)	0.77 (0.68-0.87)	< 0.001
	Four	1060 / 29560 (3.59%)	0.74 (0.65-0.85)	< 0.001
Diabete	Zero	271 / 5857 (4.63%)	-	
	One	1234 / 28851 (4.28%)	0.92 (0.8-1.05)	0.80
	Two	2265 / 60934 (3.72%)	0.79 (0.7-0.9)	0.001
	Three	2154 / 61760 (3.49%)	0.74 (0.65-0.84)	< 0.001
	Four	926 / 29560 (3.13%)	0.66 (0.58-0.76)	< 0.001
Esophageal Adenocarcinoma	Zero	20 / 5857 (0.34%)	-	
	One	87 / 28851 (0.3%)	0.88 (0.54-1.43)	1
	Two	119 / 60934 (0.2%)	0.57 (0.35-0.91)	0.08
	Three	80 / 61760 (0.13%)	0.37 (0.23-0.61)	< 0.001
	Four	23 / 29560 (0.08%)	0.22 (0.12-0.41)	< 0.001
Gastric Cardia Cancer	Zero	5 / 5857 (0.09%)	-	
	One	31 / 28851 (0.11%)	1.25 (0.49-3.22)	1
	Two	51 / 60934 (0.08%)	0.97 (0.39-2.44)	1
	Three	36 / 61760 (0.06%)	0.68 (0.26-1.72)	1
	Four	9 / 29560 (0.03%)	0.35 (0.12-1.05)	0.24
Pancreatic Cancer	Zero	10 / 5857 (0.17%)	-	
	One	54 / 28851 (0.19%)	1.09 (0.56-2.14)	1
	Two	126 / 60934 (0.21%)	1.2 (0.63-2.28)	1
	Three	109 / 61760 (0.18%)	1.02 (0.53-1.95)	1
	Four	46 / 29560 (0.16%)	0.9 (0.45-1.78)	1
Liver Cancer	Zero	8 / 5857 (0.14%)	-	
	One	39 / 28851 (0.14%)	0.98 (0.46-2.11)	1
	Two	57 / 60934 (0.09%)	0.68 (0.32-1.42)	1
	Three	45 / 61760 (0.07%)	0.53 (0.25-1.12)	0.38
	Four	23 / 29560 (0.08%)	0.56 (0.25-1.25)	0.64
Colorectal Cancer	Zero	67 / 5857 (1.14%)	-	
	One	355 / 28851 (1.23%)	1.07 (0.82-1.39)	1
	Two	578 / 60934 (0.95%)	0.82 (0.64-1.06)	0.51
	Three	530 / 61760 (0.86%)	0.74 (0.57-0.95)	0.08
	Four	228 / 29560 (0.77%)	0.66 (0.51-0.87)	0.01
Breast Cancer	Zero	65 / 5857 (1.11%)	-	
	One	333 / 28851 (1.15%)	1.04 (0.79-1.35)	1
	Two	852 / 60934 (1.4%)	1.25 (0.97-1.61)	0.33
	Three	880 / 61760 (1.42%)	1.27 (0.99-1.64)	0.25
	Four	436 / 29560 (1.47%)	1.31 (1.01-1.7)	0.16
Ovarian Cancer	Zero	11 / 5857 (0.19%)	-	
	One	45 / 28851 (0.16%)	0.83 (0.43-1.6)	1
	Two	91 / 60934 (0.15%)	0.79 (0.42-1.47)	1
	Three	105 / 61760 (0.17%)	0.89 (0.48-1.66)	1
	Four	51 / 29560 (0.17%)	0.91 (0.47-1.74)	1
Endometrial Cancer	Zero	3 / 5857 (0.05%)	-	
	One	39 / 28851 (0.14%)	2.63 (0.81-8.5)	0.43
	Two	87 / 60934 (0.14%)	2.76 (0.87-8.74)	0.33
	Three	119 / 61760 (0.19%)	3.72 (1.18-11.7)	0.10
	Four	72 / 29560 (0.24%)	4.69 (1.48-14.89)	0.03
Kidney Cancer	Zero	12 / 5857 (0.2%)	-	
	One	83 / 28851 (0.29%)	1.4 (0.76-2.56)	1
	Two	159 / 60934 (0.26%)	1.26 (0.7-2.27)	1
	Three	120 / 61760 (0.19%)	0.94 (0.52-1.7)	1
	Four	52 / 29560 (0.18%)	0.85 (0.45-1.58)	1
Hypertension	Zero	1141 / 5857 (19.48%)	-	
	One	4961 / 28851 (17.2%)	0.87 (0.81-0.92)	< 0.001
	Two	9484 / 60934 (15.56%)	0.77 (0.73-0.82)	< 0.001
	Three	8913 / 61760 (14.43%)	0.71 (0.67-0.76)	< 0.001
	Four	4194 / 29560 (14.19%)	0.7 (0.65-0.74)	< 0.001
Ischemic Heart Disease	Zero	513 / 5857 (8.76%)	-	
	One	2341 / 28851 (8.11%)	0.92 (0.84-1.01)	0.37
	Two	4311 / 60934 (7.07%)	0.8 (0.73-0.87)	< 0.001
	Three	3808 / 61760 (6.17%)	0.69 (0.63-0.76)	< 0.001
	Four	1642 / 29560 (5.55%)	0.62 (0.56-0.68)	< 0.001
Pulmonary Embolism	Zero	47 / 5857 (0.8%)	-	
	One	227 / 28851 (0.79%)	0.98 (0.71-1.34)	1
	Two	464 / 60934 (0.76%)	0.94 (0.7-1.27)	1
	Three	420 / 61760 (0.68%)	0.84 (0.62-1.13)	0.98
	Four	224 / 29560 (0.76%)	0.93 (0.68-1.27)	1
Arrhythmias	Zero	273 / 5857 (4.66%)	-	
	One	1340 / 28851 (4.64%)	0.99 (0.87-1.13)	1
	Two	2575 / 60934 (4.23%)	0.9 (0.79-1.02)	0.36
	Three	2301 / 61760 (3.73%)	0.79 (0.69-0.89)	< 0.001
	Four	992 / 29560 (3.36%)	0.71 (0.62-0.81)	< 0.001
Heart Failure	Zero	112 / 5857 (1.91%)	-	
	One	511 / 28851 (1.77%)	0.92 (0.75-1.13)	1
	Two	773 / 60934 (1.27%)	0.65 (0.54-0.8)	< 0.001
	Three	633 / 61760 (1.02%)	0.53 (0.43-0.64)	< 0.001
	Four	279 / 29560 (0.94%)	0.48 (0.39-0.6)	< 0.001
Cerebrovascular Diseases	Zero	180 / 5857 (3.07%)	-	
	One	667 / 28851 (2.31%)	0.75 (0.63-0.88)	0.002
	Two	1143 / 60934 (1.88%)	0.6 (0.51-0.7)	< 0.001
	Three	1002 / 61760 (1.62%)	0.52 (0.44-0.61)	< 0.001
	Four	440 / 29560 (1.49%)	0.47 (0.4-0.56)	< 0.001
Arteriosclerosis	Zero	9 / 5857 (0.15%)	-	
	One	68 / 28851 (0.24%)	1.53 (0.76-3.07)	0.92
	Two	68 / 60934 (0.11%)	0.72 (0.36-1.45)	1
	Three	43 / 61760 (0.07%)	0.45 (0.22-0.92)	0.12
	Four	10 / 29560 (0.03%)	0.22 (0.09-0.54)	0.004
Deep Vein Thrombosis	Zero	45 / 5857 (0.77%)	-	
	One	210 / 28851 (0.73%)	0.94 (0.68-1.3)	1
	Two	419 / 60934 (0.69%)	0.89 (0.65-1.21)	1
	Three	411 / 61760 (0.67%)	0.86 (0.63-1.16)	1
	Four	218 / 29560 (0.74%)	0.95 (0.69-1.3)	1
Asthma	Zero	256 / 5857 (4.37%)	-	
	One	1256 / 28851 (4.35%)	0.99 (0.87-1.14)	1
	Two	2547 / 60934 (4.18%)	0.95 (0.83-1.08)	1
	Three	2612 / 61760 (4.23%)	0.96 (0.84-1.09)	1
	Four	1243 / 29560 (4.21%)	0.95 (0.83-1.09)	1
Liver Disease	Zero	107 / 5857 (1.83%)	-	
	One	407 / 28851 (1.41%)	0.77 (0.62-0.95)	0.06
	Two	714 / 60934 (1.17%)	0.63 (0.52-0.78)	< 0.001
	Three	642 / 61760 (1.04%)	0.56 (0.46-0.69)	< 0.001
	Four	244 / 29560 (0.83%)	0.44 (0.35-0.56)	< 0.001
Renal Failure	Zero	220 / 5857 (3.76%)	-	
	One	979 / 28851 (3.39%)	0.9 (0.78-1.04)	0.59
	Two	1734 / 60934 (2.85%)	0.75 (0.65-0.86)	< 0.001
	Three	1526 / 61760 (2.47%)	0.65 (0.56-0.74)	< 0.001
	Four	688 / 29560 (2.33%)	0.61 (0.52-0.71)	< 0.001
Gout	Zero	70 / 5857 (1.2%)	-	
	One	233 / 28851 (0.81%)	0.67 (0.51-0.88)	0.01
	Two	351 / 60934 (0.58%)	0.48 (0.37-0.61)	< 0.001
	Three	253 / 61760 (0.41%)	0.34 (0.26-0.44)	< 0.001
	Four	83 / 29560 (0.28%)	0.23 (0.17-0.32)	< 0.001
Osteoarthritis	Zero	449 / 5857 (7.67%)	-	
	One	2357 / 28851 (8.17%)	1.06 (0.96-1.18)	0.92
	Two	4913 / 60934 (8.06%)	1.04 (0.95-1.15)	1
	Three	4916 / 61760 (7.96%)	1.03 (0.93-1.13)	1
	Four	2218 / 29560 (7.5%)	0.96 (0.87-1.07)	1
Sleep Disorders	Zero	56 / 5857 (0.96%)	-	
	One	259 / 28851 (0.9%)	0.93 (0.7-1.25)	1
	Two	443 / 60934 (0.73%)	0.75 (0.57-0.99)	0.18
	Three	375 / 61760 (0.61%)	0.63 (0.47-0.83)	0.004
	Four	161 / 29560 (0.54%)	0.56 (0.41-0.76)	< 0.001
Mood Disorders	Zero	217 / 5857 (3.7%)	-	
	One	908 / 28851 (3.15%)	0.84 (0.73-0.98)	0.10
	Two	1741 / 60934 (2.86%)	0.76 (0.66-0.88)	< 0.001
	Three	1569 / 61760 (2.54%)	0.67 (0.58-0.78)	< 0.001
	Four	681 / 29560 (2.3%)	0.61 (0.52-0.71)	< 0.001

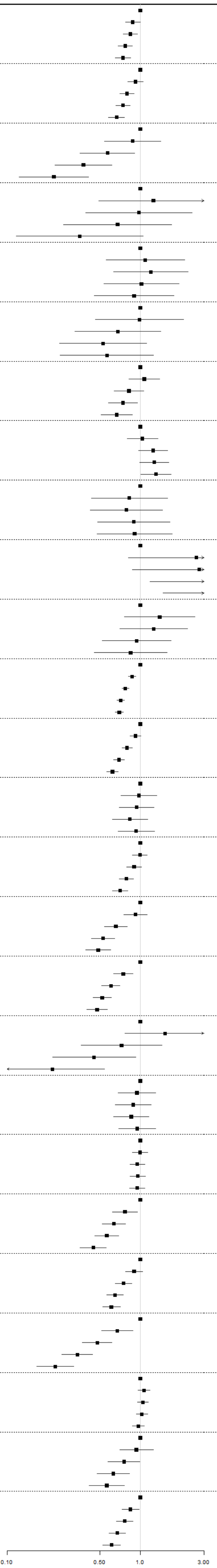

BMI, body mass index; HR, hazard ratio; CI, confidence interval

Hazard ratios (95% CI) for a lifestyle score equal to 1, 2, 3 or 4 versus a lifestyle score equal to zero were calculated in Cox proportional hazards models. All the p-values are presented after Bonferroni multiple testing adjustments.

For each lifestyle factor, individuals scored 1 if they met the criterion for a healthy lifestyle (never smoking, no or moderate alcohol consumption, physical activity, or healthy diet) and 0 otherwise. The healthy lifestyle score was computed by summing the four scores. It ranged between zero (lowest healthy score) and 4 (highest healthy score).

© 2023 Rassy N et al. *JAMA Network Open*.

eFigure 4 C. Hazard Ratios for Incident Diseases by Lifestyle Profile in Individuals With Obesity (BMI ≥30)

Disease	Healthy lifestyle factors	N (%) Cases	HR (95% CI)	Corrected pvalue
Infections	Zero	221 / 4381 (5.04%)	-	
	One	1079 / 19054 (5.66%)	1.12 (0.97-1.3)	0.47
	Two	1899 / 35517 (5.35%)	1.06 (0.92-1.21)	1
	Three	1707 / 33484 (5.1%)	1 (0.87-1.15)	1
	Four	663 / 14605 (4.54%)	0.89 (0.76-1.03)	0.49
Diabete	Zero	522 / 4381 (11.92%)	-	
	One	2218 / 19054 (11.64%)	0.97 (0.88-1.07)	1
	Two	3884 / 35517 (10.94%)	0.91 (0.83-1)	0.16
	Three	3518 / 33484 (10.51%)	0.87 (0.79-0.95)	0.009
	Four	1358 / 14605 (9.3%)	0.76 (0.69-0.84)	< 0.001
Esophageal Adenocarcinoma	Zero	11 / 4381 (0.25%)	-	
	One	61 / 19054 (0.32%)	1.27 (0.67-2.42)	1
	Two	96 / 35517 (0.27%)	1.07 (0.57-2)	1
	Three	48 / 33484 (0.14%)	0.56 (0.29-1.09)	0.35
	Four	17 / 14605 (0.12%)	0.46 (0.21-0.98)	0.17
Gastric Cardia Cancer	Zero	4 / 4381 (0.09%)	-	
	One	23 / 19054 (0.12%)	1.32 (0.46-3.81)	1
	Two	47 / 35517 (0.13%)	1.44 (0.52-4)	1
	Three	30 / 33484 (0.09%)	0.97 (0.34-2.75)	1
	Four	11 / 14605 (0.08%)	0.81 (0.26-2.55)	1
Pancreatic Cancer	Zero	8 / 4381 (0.18%)	-	
	One	41 / 19054 (0.22%)	1.17 (0.55-2.51)	1
	Two	84 / 35517 (0.24%)	1.29 (0.62-2.66)	1
	Three	75 / 33484 (0.22%)	1.21 (0.58-2.51)	1
	Four	18 / 14605 (0.12%)	0.66 (0.29-1.53)	1
Liver Cancer	Zero	4 / 4381 (0.09%)	-	
	One	37 / 19054 (0.19%)	2.12 (0.76-5.95)	0.61
	Two	71 / 35517 (0.2%)	2.18 (0.79-5.96)	0.52
	Three	36 / 33484 (0.11%)	1.16 (0.41-3.27)	1
	Four	17 / 14605 (0.12%)	1.26 (0.42-3.73)	1
Colorectal Cancer	Zero	54 / 4381 (1.23%)	-	
	One	226 / 19054 (1.19%)	0.96 (0.71-1.29)	1
	Two	346 / 35517 (0.97%)	0.79 (0.59-1.05)	0.39
	Three	278 / 33484 (0.83%)	0.67 (0.5-0.89)	0.02
	Four	123 / 14605 (0.84%)	0.67 (0.49-0.93)	0.06
Breast Cancer	Zero	51 / 4381 (1.16%)	-	
	One	264 / 19054 (1.39%)	1.19 (0.88-1.6)	1
	Two	582 / 35517 (1.64%)	1.4 (1.05-1.87)	0.08
	Three	573 / 33484 (1.71%)	1.46 (1.09-1.94)	0.04
	Four	287 / 14605 (1.97%)	1.67 (1.24-2.25)	0.003
Ovarian Cancer	Zero	4 / 4381 (0.09%)	-	
	One	29 / 19054 (0.15%)	1.66 (0.58-4.73)	1
	Two	56 / 35517 (0.16%)	1.72 (0.62-4.74)	1
	Three	67 / 33484 (0.2%)	2.17 (0.79-5.95)	0.53
	Four	39 / 14605 (0.27%)	2.89 (1.03-8.08)	0.17
Endometrial Cancer	Zero	6 / 4381 (0.14%)	-	
	One	43 / 19054 (0.23%)	1.64 (0.7-3.86)	1
	Two	137 / 35517 (0.39%)	2.8 (1.24-6.35)	0.05
	Three	164 / 33484 (0.49%)	3.54 (1.57-8)	0.009
	Four	76 / 14605 (0.52%)	3.76 (1.64-8.62)	0.007
Kidney Cancer	Zero	11 / 4381 (0.25%)	-	
	One	71 / 19054 (0.37%)	1.48 (0.78-2.79)	0.91
	Two	127 / 35517 (0.36%)	1.42 (0.76-2.62)	1
	Three	88 / 33484 (0.26%)	1.03 (0.55-1.94)	1
	Four	32 / 14605 (0.22%)	0.86 (0.43-1.71)	1
Hypertension	Zero	1120 / 4381 (25.56%)	-	
	One	4843 / 19054 (25.42%)	0.99 (0.93-1.06)	1
	Two	8270 / 35517 (23.28%)	0.9 (0.84-0.95)	0.002
	Three	7517 / 33484 (22.45%)	0.86 (0.8-0.91)	< 0.001
	Four	3081 / 14605 (21.1%)	0.8 (0.74-0.85)	< 0.001
Ischemic Heart Disease	Zero	544 / 4381 (12.42%)	-	
	One	2261 / 19054 (11.87%)	0.95 (0.87-1.05)	1
	Two	3709 / 35517 (10.44%)	0.83 (0.76-0.91)	< 0.001
	Three	2944 / 33484 (8.79%)	0.69 (0.63-0.75)	< 0.001
	Four	1031 / 14605 (7.06%)	0.55 (0.49-0.61)	< 0.001
Pulmonary Embolism	Zero	49 / 4381 (1.12%)	-	
	One	249 / 19054 (1.31%)	1.17 (0.86-1.58)	1
	Two	414 / 35517 (1.17%)	1.04 (0.77-1.39)	1
	Three	355 / 33484 (1.06%)	0.94 (0.69-1.26)	1
	Four	155 / 14605 (1.06%)	0.94 (0.68-1.29)	1
Arrhythmias	Zero	306 / 4381 (6.98%)	-	
	One	1355 / 19054 (7.11%)	1.02 (0.9-1.15)	1
	Two	2180 / 35517 (6.14%)	0.87 (0.77-0.98)	0.09
	Three	1809 / 33484 (5.4%)	0.76 (0.67-0.86)	< 0.001
	Four	609 / 14605 (4.17%)	0.58 (0.51-0.67)	< 0.001
Heart Failure	Zero	140 / 4381 (3.2%)	-	
	One	620 / 19054 (3.25%)	1.01 (0.84-1.22)	1
	Two	991 / 35517 (2.79%)	0.86 (0.72-1.03)	0.43
	Three	701 / 33484 (2.09%)	0.64 (0.54-0.77)	< 0.001
	Four	241 / 14605 (1.65%)	0.5 (0.41-0.62)	< 0.001
Cerebrovascular Diseases	Zero	114 / 4381 (2.6%)	-	
	One	537 / 19054 (2.82%)	1.08 (0.88-1.32)	1
	Two	877 / 35517 (2.47%)	0.94 (0.78-1.15)	1
	Three	756 / 33484 (2.26%)	0.86 (0.7-1.04)	0.49
	Four	248 / 14605 (1.7%)	0.64 (0.51-0.8)	< 0.001
Arteriosclerosis	Zero	11 / 4381 (0.25%)	-	
	One	42 / 19054 (0.22%)	0.88 (0.45-1.7)	1
	Two	44 / 35517 (0.12%)	0.49 (0.25-0.95)	0.14
	Three	46 / 33484 (0.14%)	0.54 (0.28-1.05)	0.28
	Four	5 / 14605 (0.03%)	0.14 (0.05-0.39)	< 0.001
Deep Vein Thrombosis	Zero	59 / 4381 (1.35%)	-	
	One	219 / 19054 (1.15%)	0.85 (0.64-1.13)	1
	Two	385 / 35517 (1.08%)	0.8 (0.61-1.05)	0.44
	Three	332 / 33484 (0.99%)	0.73 (0.55-0.96)	0.10
	Four	135 / 14605 (0.92%)	0.68 (0.5-0.92)	0.05
Asthma	Zero	243 / 4381 (5.55%)	-	
	One	1167 / 19054 (6.12%)	1.11 (0.96-1.27)	0.62
	Two	2160 / 35517 (6.08%)	1.09 (0.96-1.25)	0.74
	Three	1998 / 33484 (5.97%)	1.07 (0.93-1.22)	1
	Four	850 / 14605 (5.82%)	1.04 (0.9-1.2)	1
Liver Disease	Zero	107 / 4381 (2.44%)	-	
	One	496 / 19054 (2.6%)	1.06 (0.86-1.31)	1
	Two	792 / 35517 (2.23%)	0.91 (0.74-1.11)	1
	Three	657 / 33484 (1.96%)	0.79 (0.65-0.97)	0.11
	Four	253 / 14605 (1.73%)	0.7 (0.56-0.87)	0.007
Renal Failure	Zero	250 / 4381 (5.71%)	-	
	One	1132 / 19054 (5.94%)	1.04 (0.91-1.19)	1
	Two	1901 / 35517 (5.35%)	0.93 (0.82-1.06)	1
	Three	1546 / 33484 (4.62%)	0.8 (0.7-0.91)	0.004
	Four	566 / 14605 (3.88%)	0.67 (0.57-0.77)	< 0.001
Gout	Zero	85 / 4381 (1.94%)	-	
	One	325 / 19054 (1.71%)	0.88 (0.69-1.11)	1
	Two	458 / 35517 (1.29%)	0.66 (0.52-0.83)	0.002
	Three	287 / 33484 (0.86%)	0.43 (0.34-0.55)	< 0.001
	Four	91 / 14605 (0.62%)	0.31 (0.23-0.42)	< 0.001
Osteoarthritis	Zero	583 / 4381 (13.31%)	-	
	One	2587 / 19054 (13.58%)	1.02 (0.93-1.12)	1
	Two	4944 / 35517 (13.92%)	1.05 (0.96-1.14)	1
	Three	4612 / 33484 (13.77%)	1.03 (0.94-1.12)	1
	Four	1974 / 14605 (13.52%)	1 (0.92-1.1)	1
Sleep Disorders	Zero	152 / 4381 (3.47%)	-	
	One	603 / 19054 (3.16%)	0.91 (0.76-1.09)	1
	Two	937 / 35517 (2.64%)	0.75 (0.63-0.89)	0.005
	Three	732 / 33484 (2.19%)	0.62 (0.52-0.74)	< 0.001
	Four	274 / 14605 (1.88%)	0.53 (0.43-0.65)	< 0.001
Mood Disorders	Zero	214 / 4381 (4.88%)	-	
	One	935 / 19054 (4.91%)	1 (0.87-1.16)	1
	Two	1642 / 35517 (4.62%)	0.94 (0.82-1.09)	1
	Three	1495 / 33484 (4.46%)	0.9 (0.78-1.04)	0.68
	Four	560 / 14605 (3.83%)	0.77 (0.66-0.9)	0.005

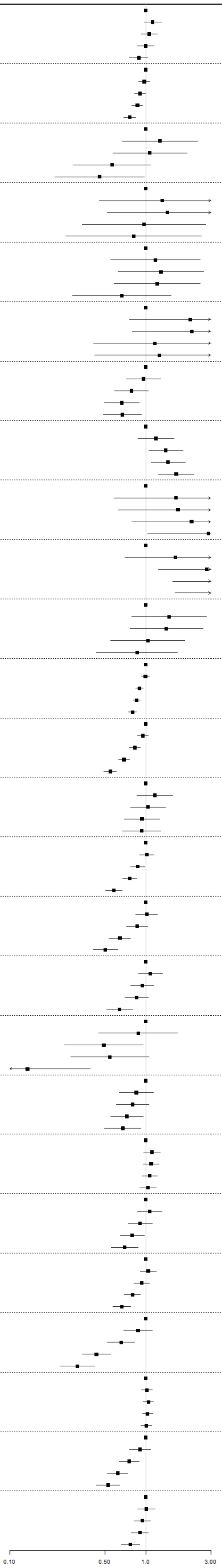

BMI, body mass index; HR, hazard ratio; CI, confidence interval

Hazard ratios (95% CI) for a lifestyle score equal to 1, 2, 3 or 4 versus a lifestyle score equal to zero were calculated in Cox proportional hazards models. All the p-values are presented after Bonferroni multiple testing adjustments.

For each lifestyle factor, individuals scored 1 if they met the criterion for a healthy lifestyle (never smoking, no or moderate alcohol consumption, physical activity, or healthy diet) and 0 otherwise. The healthy lifestyle score was computed by summing the four scores. It ranged between zero (lowest healthy score) and 4 (highest healthy score).

© 2023 Rassy N et al. *JAMA Network Open*.

eFigure 5. Adjusted Hazard Ratios for Incident Diseases by Lifestyle Profile in Individuals With Overweight (BMI 25-29.9)

BMI, body mass index; HR, hazard ratio; CI, confidence interval

Hazard ratios (95% CI) for a lifestyle score equal to 1, 2, 3 or 4 versus a lifestyle score equal to zero were calculated in Cox proportional hazards models after adjustment of age, sex, Townsend deprivation index. All the p-values are presented after Bonferroni multiple testing adjustments.

For each lifestyle factor, individuals scored 1 if they met the criterion for a healthy lifestyle (never smoking, no or moderate alcohol consumption, physical activity, or healthy diet) and 0 otherwise. The healthy lifestyle score was computed by summing the four scores. It ranged between zero (lowest healthy score) and 4 (highest healthy score).

Disease	Healthy lifestyle factors	N (%) Cases	HR (95% CI)	Corrected pvalue
Infections	Zero	278 / 5857 (4.75%)	-	
	One	1212 / 28851 (4.2%)	0.87 (0.76-0.99)	0.24
	Two	2461 / 60934 (4.04%)	0.83 (0.74-0.95)	0.03
	Three	2301 / 61760 (3.73%)	0.77 (0.68-0.87)	< 0.001
	Four	1060 / 29560 (3.59%)	0.74 (0.64-0.84)	< 0.001
Diabete	Zero	271 / 5857 (4.63%)	-	
	One	1234 / 28851 (4.28%)	0.93 (0.82-1.06)	1
	Two	2265 / 60934 (3.72%)	0.85 (0.75-0.96)	0.08
	Three	2154 / 61760 (3.49%)	0.83 (0.74-0.95)	0.04
	Four	926 / 29560 (3.13%)	0.78 (0.68-0.89)	0.002
Esophageal Adenocarcinoma	Zero	20 / 5857 (0.34%)	-	
	One	87 / 28851 (0.3%)	0.91 (0.56-1.48)	1
	Two	119 / 60934 (0.2%)	0.63 (0.39-1.01)	0.40
	Three	80 / 61760 (0.13%)	0.45 (0.28-0.74)	0.01
	Four	23 / 29560 (0.08%)	0.29 (0.16-0.53)	< 0.001
Gastric Cardia Cancer	Zero	5 / 5857 (0.09%)	-	
	One	31 / 28851 (0.11%)	1.32 (0.51-3.4)	1
	Two	51 / 60934 (0.08%)	1.13 (0.45-2.84)	1
	Three	36 / 61760 (0.06%)	0.88 (0.35-2.25)	1
	Four	9 / 29560 (0.03%)	0.51 (0.17-1.51)	1
Pancreatic Cancer	Zero	10 / 5857 (0.17%)	-	
	One	54 / 28851 (0.19%)	1.07 (0.55-2.11)	1
	Two	126 / 60934 (0.21%)	1.19 (0.63-2.28)	1
	Three	109 / 61760 (0.18%)	1.03 (0.54-1.97)	1
	Four	46 / 29560 (0.16%)	0.9 (0.45-1.79)	1
Liver Cancer	Zero	8 / 5857 (0.14%)	-	
	One	39 / 28851 (0.14%)	0.98 (0.46-2.11)	1
	Two	57 / 60934 (0.09%)	0.7 (0.33-1.47)	1
	Three	45 / 61760 (0.07%)	0.56 (0.26-1.2)	0.94
	Four	23 / 29560 (0.08%)	0.61 (0.27-1.37)	1
Colorectal Cancer	Zero	67 / 5857 (1.14%)	-	
	One	355 / 28851 (1.23%)	1.07 (0.82-1.39)	1
	Two	578 / 60934 (0.95%)	0.84 (0.65-1.08)	1
	Three	530 / 61760 (0.86%)	0.78 (0.6-1)	0.35
	Four	228 / 29560 (0.77%)	0.7 (0.54-0.93)	0.08
Breast Cancer	Zero	65 / 5857 (1.11%)	-	
	One	333 / 28851 (1.15%)	0.86 (0.66-1.12)	1
	Two	852 / 60934 (1.4%)	0.88 (0.69-1.14)	1
	Three	880 / 61760 (1.42%)	0.78 (0.61-1.01)	0.40
	Four	436 / 29560 (1.47%)	0.73 (0.56-0.94)	0.11
Ovarian Cancer	Zero	11 / 5857 (0.19%)	-	
	One	45 / 28851 (0.16%)	0.68 (0.35-1.31)	1
	Two	91 / 60934 (0.15%)	0.54 (0.29-1.02)	0.40
	Three	105 / 61760 (0.17%)	0.54 (0.29-1)	0.34
	Four	51 / 29560 (0.17%)	0.48 (0.25-0.93)	0.20
Endometrial Cancer	Zero	3 / 5857 (0.05%)	-	
	One	39 / 28851 (0.14%)	2.15 (0.66-6.95)	1
	Two	87 / 60934 (0.14%)	1.89 (0.6-5.98)	1
	Three	119 / 61760 (0.19%)	2.2 (0.7-6.92)	1
	Four	72 / 29560 (0.24%)	2.46 (0.78-7.82)	0.88
Kidney Cancer	Zero	12 / 5857 (0.2%)	-	
	One	83 / 28851 (0.29%)	1.4 (0.77-2.57)	1
	Two	159 / 60934 (0.26%)	1.31 (0.73-2.36)	1
	Three	120 / 61760 (0.19%)	1 (0.55-1.82)	1
	Four	52 / 29560 (0.18%)	0.93 (0.49-1.74)	1
Hypertension	Zero	1141 / 5857 (19.48%)	-	
	One	4961 / 28851 (17.2%)	0.86 (0.8-0.91)	< 0.001
	Two	9484 / 60934 (15.56%)	0.78 (0.73-0.83)	< 0.001
	Three	8913 / 61760 (14.43%)	0.73 (0.69-0.78)	< 0.001
	Four	4194 / 29560 (14.19%)	0.72 (0.67-0.77)	< 0.001
Ischemic Heart Disease	Zero	513 / 5857 (8.76%)	-	
	One	2341 / 28851 (8.11%)	0.94 (0.85-1.03)	1
	Two	4311 / 60934 (7.07%)	0.86 (0.79-0.95)	0.01
	Three	3808 / 61760 (6.17%)	0.79 (0.72-0.87)	< 0.001
	Four	1642 / 29560 (5.55%)	0.74 (0.67-0.82)	< 0.001
Pulmonary Embolism	Zero	47 / 5857 (0.8%)	-	
	One	227 / 28851 (0.79%)	0.98 (0.71-1.34)	1
	Two	464 / 60934 (0.76%)	0.96 (0.71-1.3)	1
	Three	420 / 61760 (0.68%)	0.88 (0.65-1.19)	1
	Four	224 / 29560 (0.76%)	0.99 (0.72-1.35)	1
Arrhythmias	Zero	273 / 5857 (4.66%)	-	
	One	1340 / 28851 (4.64%)	1 (0.88-1.14)	1
	Two	2575 / 60934 (4.23%)	0.94 (0.83-1.06)	1
	Three	2301 / 61760 (3.73%)	0.86 (0.76-0.97)	0.12
	Four	992 / 29560 (3.36%)	0.79 (0.69-0.9)	0.004
Heart Failure	Zero	112 / 5857 (1.91%)	-	
	One	511 / 28851 (1.77%)	0.93 (0.76-1.15)	1
	Two	773 / 60934 (1.27%)	0.71 (0.58-0.86)	0.004
	Three	633 / 61760 (1.02%)	0.6 (0.49-0.74)	< 0.001
	Four	279 / 29560 (0.94%)	0.58 (0.46-0.72)	< 0.001
Cerebrovascular Diseases	Zero	180 / 5857 (3.07%)	-	
	One	667 / 28851 (2.31%)	0.74 (0.63-0.88)	0.003
	Two	1143 / 60934 (1.88%)	0.62 (0.53-0.73)	< 0.001
	Three	1002 / 61760 (1.62%)	0.56 (0.47-0.65)	< 0.001
	Four	440 / 29560 (1.49%)	0.52 (0.44-0.62)	< 0.001
Arteriosclerosis	Zero	9 / 5857 (0.15%)	-	
	One	68 / 28851 (0.24%)	1.56 (0.78-3.13)	1
	Two	68 / 60934 (0.11%)	0.79 (0.39-1.58)	1
	Three	43 / 61760 (0.07%)	0.52 (0.25-1.07)	0.54
	Four	10 / 29560 (0.03%)	0.27 (0.11-0.66)	0.03
Deep Vein Thrombosis	Zero	45 / 5857 (0.77%)	-	
	One	210 / 28851 (0.73%)	0.95 (0.69-1.31)	1
	Two	419 / 60934 (0.69%)	0.92 (0.68-1.25)	1
	Three	411 / 61760 (0.67%)	0.92 (0.67-1.25)	1
	Four	218 / 29560 (0.74%)	1.03 (0.75-1.43)	1
Asthma	Zero	256 / 5857 (4.37%)	-	
	One	1256 / 28851 (4.35%)	0.98 (0.85-1.12)	1
	Two	2547 / 60934 (4.18%)	0.92 (0.81-1.04)	1
	Three	2612 / 61760 (4.23%)	0.91 (0.8-1.04)	1
	Four	1243 / 29560 (4.21%)	0.89 (0.78-1.02)	0.69
Liver Disease	Zero	107 / 5857 (1.83%)	-	
	One	407 / 28851 (1.41%)	0.77 (0.62-0.95)	0.11
	Two	714 / 60934 (1.17%)	0.65 (0.53-0.79)	< 0.001
	Three	642 / 61760 (1.04%)	0.58 (0.47-0.71)	< 0.001
	Four	244 / 29560 (0.83%)	0.46 (0.37-0.58)	< 0.001
Renal Failure	Zero	220 / 5857 (3.76%)	-	
	One	979 / 28851 (3.39%)	0.89 (0.77-1.03)	0.88
	Two	1734 / 60934 (2.85%)	0.77 (0.67-0.88)	0.001
	Three	1526 / 61760 (2.47%)	0.68 (0.59-0.78)	< 0.001
	Four	688 / 29560 (2.33%)	0.65 (0.56-0.76)	< 0.001
Gout	Zero	70 / 5857 (1.2%)	-	
	One	233 / 28851 (0.81%)	0.71 (0.55-0.93)	0.09
	Two	351 / 60934 (0.58%)	0.56 (0.44-0.73)	< 0.001
	Three	253 / 61760 (0.41%)	0.45 (0.34-0.58)	< 0.001
	Four	83 / 29560 (0.28%)	0.34 (0.25-0.47)	< 0.001
Osteoarthritis	Zero	449 / 5857 (7.67%)	-	
	One	2357 / 28851 (8.17%)	1.02 (0.93-1.13)	1
	Two	4913 / 60934 (8.06%)	0.99 (0.89-1.09)	1
	Three	4916 / 61760 (7.96%)	0.95 (0.86-1.05)	1
	Four	2218 / 29560 (7.5%)	0.87 (0.79-0.96)	0.05
Sleep Disorders	Zero	56 / 5857 (0.96%)	-	
	One	259 / 28851 (0.9%)	0.97 (0.73-1.3)	1
	Two	443 / 60934 (0.73%)	0.83 (0.63-1.1)	1
	Three	375 / 61760 (0.61%)	0.73 (0.55-0.96)	0.18
	Four	161 / 29560 (0.54%)	0.68 (0.5-0.92)	0.09
Mood Disorders	Zero	217 / 5857 (3.7%)	-	
	One	908 / 28851 (3.15%)	0.83 (0.71-0.96)	0.08
	Two	1741 / 60934 (2.86%)	0.73 (0.63-0.84)	< 0.001
	Three	1569 / 61760 (2.54%)	0.63 (0.54-0.72)	< 0.001
	Four	681 / 29560 (2.3%)	0.55 (0.48-0.65)	< 0.001

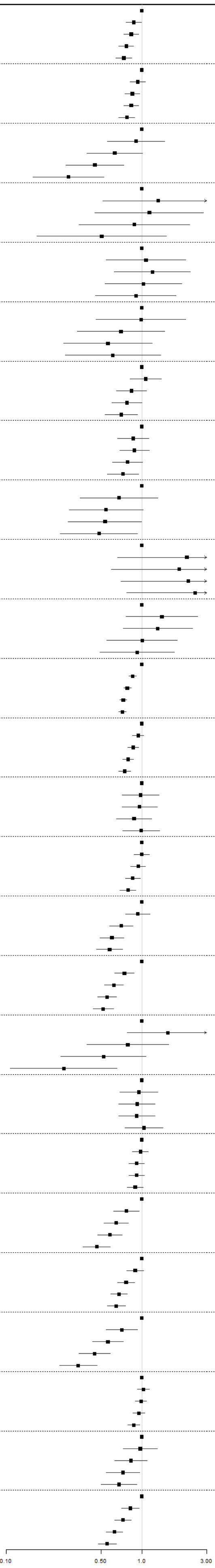

© 2023 Rassy N et al. *JAMA Network Open*.

A.

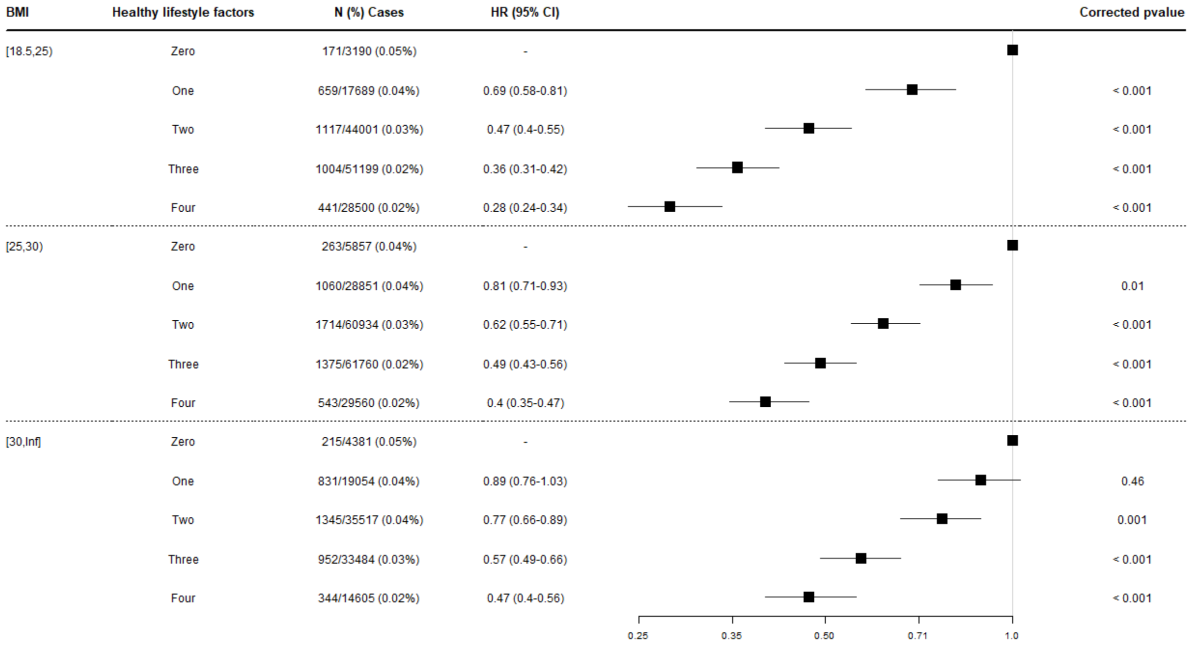

B.

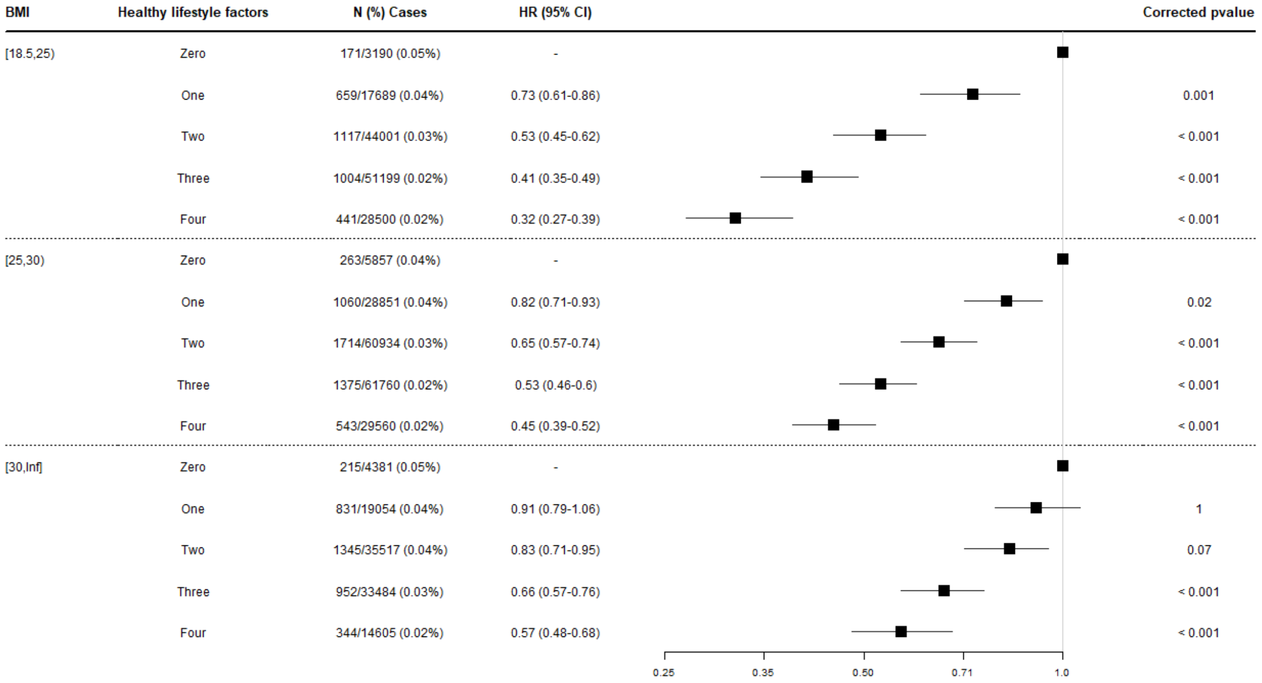

eFigure 6. Hazard ratios for All-Cause Mortality by Lifestyle Profile in Individuals With Normal Weight (BMI 18.5-24.9), Overweight (BMI 25-29.9), and Obesity (BMI ≥30) Before (A) and After Adjustment (B) for Age, Sex, and Townsend Deprivation Index

BMI, body mass index; HR, hazard ratio; CI, confidence interval

Hazard ratios (95% CI) for a lifestyle score equal to 1, 2, 3 or 4 versus a lifestyle score equal to zero were calculated in Cox proportional hazards models. All the p-values are presented after Bonferroni multiple testing adjustments.

For each lifestyle factor, individuals scored 1 if they met the criterion for a healthy lifestyle (never smoking, no or moderate alcohol consumption, physical activity, or healthy diet) and 0 otherwise. The healthy lifestyle score was computed by summing the four scores. It ranged between zero (lowest healthy score) and 4 (highest healthy score).

© 2023 Rassy N et al. *JAMA Network Open*.

eTable 9. Associations of Body Mass Index With Incident Diseases by Healthy Lifestyle Score After Exclusion of Participants With Diseases Diagnosed Within the First 2 Years of Follow-up

Diseases	Healthy lifestyle score<br>Adjusted HR (95% CI)[a]				
	4	3	2	1	0
**Infections**					
BMI 18.5-24.9 kg/m²	1 (Reference)	1.12 (1.02-1.23)	1.27 (1.16-1.39)	1.53 (1.37-1.71)	1.90 (1.58-2.29)
BMI 25-29.9 kg/m²	1.21 (1.09-1.34)	1.24 (1.14-1.36)	1.33 (1.22-1.46)	1.41 (1.28-1.56)	1.59 (1.36-1.86)
BMI ≥ 30 kg/m²	1.45 (1.29-1.62)	1.66 (1.51-1.82)	1.65 (1.5-1.81)	1.85 (1.67-2.05)	1.59 (1.34-1.9)
**Diabetes**					
BMI 18.5-24.9 kg/m²	1 (Reference)	1.18 (1.01-1.37)	1.19 (1.02-1.40)	1.24 (1.03-1.50)	1.43 (1.03-1.98)
BMI 25-29.9 kg/m²	2.47 (2.13-2.87)	2.69 (2.35-3.09)	2.75 (2.40-3.15)	3.16 (2.74-3.65)	3.29 (2.73-3.98)
BMI ≥ 30 kg/m²	8.04 (6.98-9.26)	8.54 (7.48-9.75)	8.43 (7.38-9.62)	8.84 (7.71-10.13)	8.85 (7.52-10.42)
**Esophageal adenocarcinoma**					
BMI 18.5-24.9 kg/m²	1 (Reference)	0.97 (0.51-1.83)	1.93 (1.08-3.47)	2.07 (1.06-4.01)	3.10 (1.20-1.80)
BMI 25-29.9 kg/m²	0.97 (0.49-1.93)	1.38 (0.78-2.45)	2.13 (1.23-3.69)	2.99 (1.70-5.27)	3.86 (1.92-7.74)
BMI ≥ 30 kg/m²	1.81 (0.87-3.76)	1.53 (0.82-2.85)	2.96 (1.69-5.18)	2.94 (1.62-5.36)	2.52 (1.07-5.96)
**Gastric cardia cancer**					
BMI 18.5-24.9 kg/m²	1 (Reference)	1.04 (0.41-2.6)	1.11 (0.44-2.82)	1.25 (0.42-3.71)	2.17 (0.45-10.45)
BMI 25-29.9 kg/m²	0.67 (0.23-20)	1.26 (0.54-2.91)	2.06 (0.92-4.57)	2.02 (0.86-4.45)	1.75 (0.51-6.01)
BMI ≥ 30 kg/m²	2.93 (1.12-7.71)	2.21 (0.93-5.23)	3.49 (1.55-7.83)	1.72 (0.67-4.46)	2.51 (0.73-8.61)
**Pancreatic cancer**					
BMI 18.5-24.9 kg/m²	1 (Reference)	0.91 (0.58-1.44)	1.18 (0.75-1.84)	1.60 (0.96-2.67)	1.24 (0.44-3.53)
BMI 25-29.9 kg/m²	1.09 (0.67-1.77)	1.19 (0.79-1.81)	1.40 (0.93-2.11)	1.32 (0.83-2.11)	1.21 (0.55-2.65)
BMI ≥ 30 kg/m²	1.10 (0.61-2.00)	1.46 (0.93-2.29)	1.65 (1.07-2.55)	1.57 (0.95-2.59)	1.27 (0.53-3.06)
**Liver cancer**					
BMI 18.5-24.9 kg/m²	1 (Reference)	2.09 (1.00-4.37)	1.78 (0.83-3.84)	3.3 (1.48-7.36)	5.15 (1.72-15.38)
BMI 25-29.9 kg/m²	1.93 (0.88-4.24)	1.43 (0.68-3.01)	1.70 (0.82-3.53)	2.6 (1.22-5.54)	2.90 (1.03-8.16)
BMI ≥ 30 kg/m²	3.29 (1.44-7.52)	2.15 (1-4.66)	3.62 (1.76-7.45)	4.3 (2.02-9.18)	0.68 (0.09-5.4)
**Colorectal cancer**					
BMI 18.5-24.9 kg/m²	1 (Reference)	1.08 (0.88-1.33)	1.18 (0.96-1.45)	1.29 (1.00-1.66)	0.80 (0.45-1.45)
BMI 25-29.9 kg/m²	1.14 (0.92-1.43)	1.19 (0.98-1.45)	1.32 (1.09-1.60)	1.75 (1.43-2.15)	1.27 (0.90-1.81)
BMI ≥ 30 kg/m²	1.30 (0.99-1.69)	1.27 (1.03-1.58)	1.40 (1.14-1.73)	1.65 (1.31-2.08)	1.67 (1.16-2.41)
**Breast cancer**					
BMI 18.5-24.9 kg/m²	1 (Reference)	1.08 (0.95-1.23)	1.17 (1.02-1.33)	1.32 (1.11-1.55)	1.08 (0.74-1.56)
BMI 25-29.9 kg/m²	1.06 (0.91-1.24)	1.17 (1.03-1.34)	1.33 (1.17-1.51)	1.26 (1.07-1.49)	1.45 (1.07-1.97)
BMI ≥ 30 kg/m²	1.25 (1.05-1.48)	1.28 (1.11-1.47)	1.4 (1.22-1.62)	1.45 (1.21-1.73)	1.46 (1.01-2.06)
**Ovarian cancer**					
BMI 18.5-24.9 kg/m²	1 (Reference)	1.04 (0.72-1.52)	0.95 (0.64-1.40)	0.83 (0.48-1.44)	0.60 (0.15-2.49)
BMI 25-29.9 kg/m²	0.96 (0.62-1.48)	1.19 (0.82-1.71)	1.19 (0.81-1.73)	1.34 (0.85-2.11)	2.71 (1.40-5.25)
BMI ≥ 30 kg/m²	1.29 (0.80-2.07)	1.18 (0.78-1.77)	1.11 (0.73-1.70)	1.34 (0.80-2.24)	0.99 (0.31-3.2)
**Endometrial cancer**					
BMI 18.5-24.9 kg/m²	1 (Reference)	1.05 (0.71-1.55)	0.73 (0.47-1.13)	0.87 (0.49-1.54)	0 (0-inf)
BMI 25-29.9 kg/m²	1.48 (0.98-2.24)	1.41 (0.97-2.05)	1.21 (0.82-1.8)	1.36 (0.84-2.19)	0.55 (0.13-2.28)
BMI ≥ 30 kg/m²	2.93 (1.95-4.40)	3.00 (2.09-4.32)	2.82 (1.94-4.1)	2.05 (1.27-3.3)	1.12 (0.35-3.62)
**Kidney cancer**					
BMI 18.5-24.9 kg/m²	1 (Reference)	1.18 (0.74-1.88)	0.89 (0.54-1.48)	1.06 (0.57-1.95)	1.37 (0.48-3.94)
BMI 25-29.9 kg/m²	1.39 (0.85-2.27)	1.50 (0.97-2.32)	1.97 (1.29-3.01)	1.91 (1.20-3.03)	0.79 (0.30-2.05)
BMI ≥ 30 kg/m²	1.86 (1.07-3.24)	2.07 (1.31-3.27)	2.39 (1.54-3.73)	2.71 (1.68-4.35)	1.79 (0.81-3.96)
**Hypertension**					
BMI 18.5-24.9 kg/m²	1 (Reference)	1.04 (0.98-1.11)	1.16 (1.10-1.23)	1.42 (1.33-1.53)	1.82 (1.62-2.04)
BMI 25-29.9 kg/m²	1.70 (1.60-1.80)	1.72 (1.63-1.81)	1.85 (1.76-1.95)	2.02 (1.90-2.13)	2.31 (2.13-2.51)
BMI ≥ 30 kg/m²	2.72 (2.55-2.89)	2.90 (2.74-3.06)	2.93 (2.78-3.10)	3.22 (3.04-3.41)	3.31 (3.05-3.60)
**Ischemic heart disease**					
BMI 18.5-24.9 kg/m²	1 (Reference)	1.08 (0.99-1.19)	1.21 (1.10-1.33)	1.49 (1.34-1.67)	2.02 (1.70-2.40)
BMI 25-29.9 kg/m²	1.50 (1.36-1.65)	1.57 (1.44-1.71)	1.72 (1.58-1.87)	1.90 (1.74-2.08)	2.06 (1.81-2.34)
BMI ≥ 30 kg/m²	2.13 (1.92-2.37)	2.41 (2.21-2.63)	2.60 (2.38-2.83)	2.83 (2.58-3.10)	2.94 (2.59-3.34)

© 2023 Rassy N et al. *JAMA Network Open*.

Diseases	Healthy lifestyle score<br>Adjusted HR (95% CI)[a]				
	4	3	2	1	0
**Pulmonary embolism**					
BMI 18.5-24.9 kg/m²	1 (Reference)	1.07 (0.82-1.39)	1.14 (0.87-1.49)	1.40 (1.03-1.92)	3.11 (2.04-4.75)
BMI 25-29.9 kg/m²	1.75 (1.34-2.27)	1.58 (1.24-2.01)	1.84 (1.46-2.34)	1.70 (1.31-2.22)	1.74 (1.17-2.61)
BMI ≥ 30 kg/m²	2.50 (1.88-3.33)	2.57 (2.01-3.28)	2.67 (2.09-3.4)	3.15 (2.43-4.08)	2.58 (1.74-3.83)
**Arrhythmias**					
BMI 18.5-24.9 kg/m²	1 (Reference)	1.00 (0.91-1.10)	1.04 (0.94-1.15)	1.16 (1.03-1.31)	1.42 (1.15-1.76)
BMI 25-29.9 kg/m²	1.02 (0.92-1.14)	1.11 (1.01-1.22)	1.21 (1.10-1.32)	1.28 (1.16-1.41)	1.31 (1.12-1.54)
BMI ≥ 30 kg/m²	1.43 (1.26-1.61)	1.68 (1.53-1.85)	1.80 (1.64-1.98)	2.03 (1.83-2.25)	2.05 (1.76-2.39)
**Heart failure**					
BMI 18.5-24.9 kg/m²	1 (Reference)	1.02 (0.83-1.26)	1.28 (1.04-1.57)	1.65 (1.30-2.08)	2.31 (1.61-3.31)
BMI 25-29.9 kg/m²	1.25 (1.01-1.56)	1.27 (1.05-1.54)	1.47 (1.22-1.78)	1.98 (1.62-2.41)	1.95 (1.46-2.59)
BMI ≥ 30 kg/m²	2.43 (1.94-3.05)	2.42 (2.00-2.94)	3.15 (2.26-3.80)	3.69 (3.03-4.48)	3.37 (2.57-4.41)
**Cerebrovascular diseases**					
BMI 18.5-24.9 kg/m²	1 (Reference)	0.91 (0.79-1.06)	1.12 (0.97-1.30)	1.45 (1.22-1.72)	1.84 (1.39-2.43)
BMI 25-29.9 kg/m²	1.02 (0.87-1.20)	1.07 (0.93-1.23)	1.17 (1.02-1.35)	1.43 (1.24-1.66)	1.81 (1.46-2.24)
BMI ≥ 30 kg/m²	1.21 (1.00-1.46)	1.49 (1.29-1.72)	1.50 (1.30-1.74)	1.61 (1.37-1.89)	1.36 (1.04-1.77)
**Arteriosclerosis**					
BMI 18.5-24.9 kg/m²	1 (Reference)	1.10 (0.28-4.41)	3.71 (1.09-12.60)	6.73 (1.94-23.26)	18.06 (4.78-68.28)
BMI 25-29.9 kg/m²	0.78 (0.16-3.89)	2.34 (0.69-7.88)	3.81 (1.17-12.42)	7.86 (2.42-25.55)	2.04 (0.34-12.25)
BMI ≥ 30 kg/m²	0.61 (0.06-5.85)	4.42 (1.31-14.96)	3.76 (1.11-12.72)	5.49 (1.60-18.89)	10 (2.87-41.15)
**Deep vein thrombosis**					
BMI 18.5-24.9 kg/m²	1 (Reference)	0.85 (0.66-1.09)	1.09 (0.85-1.39)	1.28 (0.95-1.71)	2.10 (1.35-3.27)
BMI 25-29.9 kg/m²	1.36 (1.06-1.75)	1.16 (0.93-1.46)	1.24 (0.99-1.55)	1.24 (0.96-1.59)	1.43 (0.96-2.12)
BMI ≥ 30 kg/m²	1.87 (1.42-2.47)	1.83 (1.45-2.31)	1.83 (1.45-2.31)	2.04 (1.58-2.63)	2.41 (1.67-3.48)
**Asthma**					
BMI 18.5-24.9 kg/m²	1 (Reference)	0.99 (0.90-1.08)	1.03 (0.94-1.13)	1.22 (1.09-1.36)	1.58 (1.30-1.92)
BMI 25-29.9 kg/m²	1.28 (1.16-1.41)	1.30 (1.19-1.41)	1.31 (1.20-1.42)	1.36 (1.23-1.50)	1.42 (1.21-1.66)
BMI ≥ 30 kg/m²	1.68 (1.51-1.87)	1.78 (1.63-1.95)	1.84 (1.69-2.01)	1.88 (1.70-2.08)	1.82 (1.55-2.15)
**Liver disease**					
BMI 18.5-24.9 kg/m²	1 (Reference)	1.12 (0.89-1.41)	1.58 (1.26-1.97)	2.58 (2.04-3.28)	3.19 (2.22-4.58)
BMI 25-29.9 kg/m²	1.68 (1.33-2.12)	1.85 (1.50-2.28)	2.03 (1.65-2.50)	2.67 (2.14-3.32)	3.15 (2.34-4.24)
BMI ≥ 30 kg/m²	3.10 (2.44-3.94)	3.42 (2.78-4.22)	4.02 (3.27-4.94)	4.37 (3.51-5.43)	4.26 (3.17-5.73)
**Renal failure**					
BMI 18.5-24.9 kg/m²	1 (Reference)	0.97 (0.85-1.11)	1.15 (1.00-1.31)	1.55 (1.33-1.80)	1.89 (1.47-2.45)
BMI 25-29.9 kg/m²3	1.35 (1.17-1.55)	1.43 (1.27-1.62)	1.58 (1.40-1.78)	1.87 (1.64-2.13)	1.97 (1.63-2.38)
BMI ≥ 30 kg/m²	2.31 (2.00-2.67)	2.57 (2.27-2.91)	2.89 (2.56-3.26)	3.19 (2.80-3.63)	2.97 (2.47-3.58)
**Gout**					
BMI 18.5-24.9 kg/m²	1 (Reference)	1.60 (0.92-2.77)	2.04 (1.19-3.52)	2.51 (1.39-4.53)	5.31 (2.58-10.93)
BMI 25-29.9 kg/m²	2.99 (1.75-5.09)	4.05 (2.47-6.65)	5.09 (3.12-8.31)	6.72 (4.09-11.04)	8.91 (5.16-15.40)
BMI ≥ 30 kg/m²	7.73 (4.53-13.19)	8.88 (5.42-14.57)	11.29 (6.93-18.39)	14.34 (8.76-23.47)	15.67 (9.19-26.73)
**Osteoarthritis**					
BMI 18.5-24.9 kg/m²	1 (Reference)	1.11 (1.03-1.20)	1.15 (1.06-1.24)	1.23 (1.12-1.35)	1.03 (0.84-1.26)
BMI 25-29.9 kg/m²	1.59 (1.47-1.72)	1.72 (1.61-1.85)	1.79 (1.67-1.92)	1.84 (1.71-1.99)	1.87 (1.65-2.11)
BMI ≥ 30 kg/m²	2.87 (2.65-3.11)	2.96 (2.76-3.17)	3.12 (2.91-3.35)	3.21 (2.97-3.46)	3.24 (2.89-3.63)
**Sleep disorders**					
BMI 18.5-24.9 kg/m²	1 (Reference)	0.96 (0.70-1.32)	1.06 (0.77-1.45)	1.28 (0.88-1.84)	1.64 (0.90-2.98)
BMI 25-29.9 kg/m²	1.76 (1.29-2.39)	1.81 (1.37-2.39)	2.10 (1.60-2.76)	2.37 (1.77-3.17)	2.34 (1.56-3.53)
BMI ≥ 30 kg/m²	5.96 (4.47-7.94)	6.13 (4.70-8.00)	7.21 (5.55-9.37)	8.41 (6.43-11.00)	8.92 (6.50-12.24)
**Mood disorders**					
BMI 18.5-24.9 kg/m²	1 (Reference)	1.19 (1.06-1.34)	1.39 (1.24-1.56)	1.94 (1.70-2.21)	2.23 (1.78-2.79)
BMI 25-29.9 kg/m²	1.38 (1.21-1.57)	1.49 (1.33-1.67)	1.71 (1.53-1.91)	1.98 (1.75-2.23)	2.23 (1.85-2.68)
BMI ≥ 30 kg/m²	1.98 (1.73-2.28)	2.41 (2.14-2.70)	2.52 (2.25-2.83)	2.83 (2.50-3.21)	3.21 (2.68-3.85)

[a] HR adjusted for age, sex, and Townsend deprivation index. Healthy lifestyle score included 4 lifestyle factors (never smoking, no or moderate alcohol consumption, physical activity, healthy diet). Participants scored 1 if they met the criterion for a healthy lifestyle and 0 otherwise

© 2023 Rassy N et al. *JAMA Network Open*.

eTable 10. Hazard ratios for Incident Diseases by Lifestyle Profile in Each Body Mass Index Category After Exclusion of Participants With Diseases Diagnosed Within the First 2 Years of Follow-up

Healthy lifestyle factor				HR (95% CI)			
**Physically active**	**No or moderate alcohol consumption**	**Never smoking**	**Healthy diet**				
				**Total population (n = 438 583)**	**BMI 18.5-24.9 (n = 144 579)**	**BMI 25-29.9 (n = 186 963)**	**BMI ≥ 30 (n = 107 041)**
No	No	No	No	*Reference*	*Reference*	*Reference*	*Reference*
No	No	No	Yes	0.71 (0.68-0.75)	0.61 (0.55-0.68)	0.71 (0.66-0.77)	0.82 (0.76-0.89)
No	No	Yes	No	0.92 (0.88-0.96)	0.91 (0.83-1.00)	0.92 (0.86-0.98)	1.01 (0.94-1.08)
No	No	Yes	Yes	0.70 (0.67-0.74)	0.66 (0.60-0.72)	0.75 (0.70-0.80)	0.80 (0.74-0.86)
No	Yes	No	No	1.13 (1.09-1.18)	1.04 (0.95-1.15)	1.11 (1.04-1.19)	1.16 (1.09-1.23)
No	Yes	No	Yes	0.77 (0.74-0.80)	0.63 (0.58-0.70)	0.76 (0.71-0.81)	0.88 (0.83-0.94)
No	Yes	Yes	No	1.08 (1.04-1.13)	0.92 (0.84-1.01)	1.08 (1.01-1.15)	1.18 (1.11-1.25)
No	Yes	Yes	Yes	0.82 (0.79-0.85)	0.70 (0.65-0.76)	0.83 (0.78-0.88)	0.96 (0.9-1.02)
Yes	No	No	No	0.86 (0.83-0.89)	0.84 (0.77-0.92)	0.91 (0.86-0.96)	0.92 (0.86-0.98)
Yes	No	No	Yes	0.62 (0.60-0.65)	0.58 (0.53-0.63)	0.67 (0.62-0.71)	0.75 (0.69-0.80)
Yes	No	Yes	No	0.78 (0.76-0.81)	0.74 (0.68-0.79)	0.88 (0.84-0.93)	0.92 (0.87-0.98)
Yes	No	Yes	Yes	0.61 (0.59-0.63)	0.61 (0.56-0.66)	0.69 (0.66-0.73)	0.72 (0.68-0.77)
Yes	Yes	No	No	0.97 (0.93-1.00)	0.90 (0.82-0.98)	1.00 (0.95-1.06)	1.02 (0.96-1.09)
Yes	Yes	No	Yes	0.67 (0.64-0.69)	0.61 (0.56-0.66)	0.71 (0.67-0.75)	0.80 (0.75-0.85)
Yes	Yes	Yes	No	0.89 (0.86-0.93)	0.82 (0.76-0.89)	0.94 (0.89-0.99)	1.04 (0.99-1.11)
Yes	Yes	Yes	Yes	0.69 (0.67-0.71)	0.65 (0.6-0.69)	0.77 (0.73-0.81)	0.85 (0.80-0.90)

HR, hazard ratio; CI, confidence interval

© 2023 Rassy N et al. *JAMA Network Open*.

eTable 11. Adjusted Hazard Ratios for Incident Diseases by Lifestyle Profile in Each Body Mass Index Category After Exclusion of Participants With Diseases Diagnosed Within the First 2 Years of Follow-up

Healthy lifestyle factor				HR (95% CI)[a]			
**Physically active**	**No or moderate alcohol consumption**	**Never smoking**	**Healthy diet**				
				**Total population (n = 438 583)**	**BMI 18.5-24.9 (n = 144 579)**	**BMI 25-29.9 (n = 186 963)**	**BMI ≥ 30 (n = 107 041)**
No	No	No	No	*Reference*	*Reference*	*Reference*	*Reference*
No	No	No	Yes	0.84 (0.80-0.88)	0.70 (0.63-0.79)	0.81 (0.75-0.88)	0.93 (0.86-1.01)
No	No	Yes	No	0.92 (0.89-0.96)	0.89 (0.81-0.98)	0.88 (0.82-0.94)	0.98 (0.92-1.05)
No	No	Yes	Yes	0.79 (0.76-0.83)	0.69 (0.63-0.77)	0.78 (0.72-0.83)	0.86 (0.80-0.93)
No	Yes	No	No	1.07 (1.03-1.11)	1.02 (0.93-1.12)	1.07 (1.01-1.14)	1.11 (1.04-1.18)
No	Yes	No	Yes	0.85 (0.82-0.89)	0.71 (0.65-0.78)	0.84 (0.79-0.90)	0.95 (0.89-1.01)
No	Yes	Yes	No	0.99 (0.95-1.03)	0.87 (0.79-0.95)	0.99 (0.93-1.06)	1.07 (1.00-1.13)
No	Yes	Yes	Yes	0.86 (0.83-0.89)	0.72 (0.66-0.78)	0.84 (0.80-0.89)	0.96 (0.91-1.02)
Yes	No	No	No	0.88 (0.85-0.92)	0.81 (0.75-0.89)	0.87 (0.82-0.92)	0.92 (0.86-0.98)
Yes	No	No	Yes	0.74 (0.71-0.77)	0.64 (0.58-0.70)	0.73 (0.68-0.78)	0.84 (0.78-0.90)
Yes	No	Yes	No	0.82 (0.79-0.85)	0.70 (0.65-0.76)	0.83 (0.78-0.87)	0.90 (0.84-0.95)
Yes	No	Yes	Yes	0.70 (0.68-0.73)	0.62 (0.57-0.67)	0.70 (0.66-0.74)	0.78 (0.73-0.83)
Yes	Yes	No	No	0.94 (0.90-0.97)	0.84 (0.77-0.92)	0.94 (0.89-1.00)	0.98 (0.92-1.04)
Yes	Yes	No	Yes	0.77 (0.74-0.80)	0.65 (0.60-0.70)	0.76 (0.72-0.81)	0.87 (0.82-0.93)
Yes	Yes	Yes	No	0.86 (0.83-0.90)	0.76 (0.70-0.82)	0.85 (0.80-0.89)	0.96 (0.91-1.02)
Yes	Yes	Yes	Yes	0.75 (0.73-0.78)	0.64 (0.59-0.69)	0.76 (0.72-0.80)	0.86 (0.82-0.91)

HR, hazard ratio; CI, confidence interval
[a] HR adjusted for age, sex, BMI, and Townsend deprivation index

© 2023 Rassy N et al. *JAMA Network Open*.

Disease	BMI	N (%) Cases	HR (95% CI)	Adjusted pvalue
Infections	18.5 ≤ BMI < 25	4058 / 137041 (2.96%)	-	
	25 ≤ BMI < 30	5725 / 172714 (3.31%)	1.12 (1.08-1.17)	<0.001
	BMI ≥ 30	4023 / 94004 (4.28%)	1.46 (1.4-1.53)	<0.001
Diabete	18.5 ≤ BMI < 25	1426 / 137041 (1.04%)	-	
	25 ≤ BMI < 30	4983 / 172714 (2.89%)	2.8 (2.64-2.97)	<0.001
	BMI ≥ 30	8033 / 94004 (8.55%)	8.57 (8.1-9.07)	<0.001
Esophageal Adenocarcinoma	18.5 ≤ BMI < 25	113 / 137041 (0.08%)	-	
	25 ≤ BMI < 30	246 / 172714 (0.14%)	1.73 (1.38-2.16)	<0.001
	BMI ≥ 30	157 / 94004 (0.17%)	2.04 (1.6-2.59)	<0.001
Gastric Cardia Cancer	18.5 ≤ BMI < 25	40 / 137041 (0.03%)	-	
	25 ≤ BMI < 30	101 / 172714 (0.06%)	2.01 (1.39-2.89)	<0.001
	BMI ≥ 30	83 / 94004 (0.09%)	3.04 (2.08-4.43)	<0.001
Pancreatic Cancer	18.5 ≤ BMI < 25	164 / 137041 (0.12%)	-	
	25 ≤ BMI < 30	271 / 172714 (0.16%)	1.31 (1.08-1.59)	0.012
	BMI ≥ 30	170 / 94004 (0.18%)	1.52 (1.23-1.88)	<0.001
Liver Cancer	18.5 ≤ BMI < 25	89 / 137041 (0.06%)	-	
	25 ≤ BMI < 30	121 / 172714 (0.07%)	1.08 (0.82-1.42)	1
	BMI ≥ 30	108 / 94004 (0.11%)	1.78 (1.34-2.35)	<0.001
Colorectal Cancer	18.5 ≤ BMI < 25	764 / 137041 (0.56%)	-	
	25 ≤ BMI < 30	1316 / 172714 (0.76%)	1.37 (1.25-1.5)	<0.001
	BMI ≥ 30	732 / 94004 (0.78%)	1.4 (1.27-1.55)	<0.001
Breast Cancer	18.5 ≤ BMI < 25	1873 / 137041 (1.37%)	-	
	25 ≤ BMI < 30	1942 / 172714 (1.12%)	0.82 (0.77-0.88)	<0.001
	BMI ≥ 30	1275 / 94004 (1.36%)	1 (0.93-1.07)	1
Ovarian Cancer	18.5 ≤ BMI < 25	200 / 137041 (0.15%)	-	
	25 ≤ BMI < 30	243 / 172714 (0.14%)	0.97 (0.8-1.16)	1
	BMI ≥ 30	145 / 94004 (0.15%)	1.06 (0.86-1.31)	1
Endometrial Cancer	18.5 ≤ BMI < 25	168 / 137041 (0.12%)	-	
	25 ≤ BMI < 30	246 / 172714 (0.14%)	1.16 (0.96-1.42)	0.259
	BMI ≥ 30	299 / 94004 (0.32%)	2.61 (2.16-3.15)	<0.001
Kidney Cancer	18.5 ≤ BMI < 25	136 / 137041 (0.1%)	-	
	25 ≤ BMI < 30	331 / 172714 (0.19%)	1.94 (1.58-2.36)	<0.001
	BMI ≥ 30	229 / 94004 (0.24%)	2.47 (2-3.05)	<0.001
Hypertension	18.5 ≤ BMI < 25	9818 / 137041 (7.16%)	-	
	25 ≤ BMI < 30	21693 / 172714 (12.56%)	1.81 (1.77-1.85)	<0.001
	BMI ≥ 30	18007 / 94004 (19.16%)	2.88 (2.81-2.95)	<0.001
Ischemic Heart Disease	18.5 ≤ BMI < 25	3948 / 137041 (2.88%)	-	
	25 ≤ BMI < 30	8843 / 172714 (5.12%)	1.8 (1.73-1.87)	<0.001
	BMI ≥ 30	6832 / 94004 (7.27%)	2.59 (2.49-2.7)	<0.001
Pulmonary Embolism	18.5 ≤ BMI < 25	495 / 137041 (0.36%)	-	
	25 ≤ BMI < 30	1040 / 172714 (0.6%)	1.67 (1.5-1.86)	<0.001
	BMI ≥ 30	871 / 94004 (0.93%)	2.58 (2.31-2.89)	<0.001
Arrhythmias	18.5 ≤ BMI < 25	3215 / 137041 (2.35%)	-	
	25 ≤ BMI < 30	5554 / 172714 (3.22%)	1.38 (1.32-1.44)	<0.001
	BMI ≥ 30	4302 / 94004 (4.58%)	1.98 (1.89-2.07)	<0.001
Heart Failure	18.5 ≤ BMI < 25	833 / 137041 (0.61%)	-	
	25 ≤ BMI < 30	1623 / 172714 (0.94%)	1.55 (1.43-1.69)	<0.001
	BMI ≥ 30	1699 / 94004 (1.81%)	3 (2.77-3.26)	<0.001
Cerebrovascular Diseases	18.5 ≤ BMI < 25	1521 / 137041 (1.11%)	-	
	25 ≤ BMI < 30	2506 / 172714 (1.45%)	1.31 (1.23-1.4)	<0.001
	BMI ≥ 30	1672 / 94004 (1.78%)	1.62 (1.51-1.73)	<0.001
Arteriosclerosis	18.5 ≤ BMI < 25	50 / 137041 (0.04%)	-	
	25 ≤ BMI < 30	97 / 172714 (0.06%)	1.54 (1.1-2.17)	0.026
	BMI ≥ 30	63 / 94004 (0.07%)	1.84 (1.27-2.67)	0.003
Deep Vein Thrombosis	18.5 ≤ BMI < 25	533 / 137041 (0.39%)	-	
	25 ≤ BMI < 30	908 / 172714 (0.53%)	1.35 (1.22-1.51)	<0.001
	BMI ≥ 30	741 / 94004 (0.79%)	2.04 (1.83-2.28)	<0.001
Asthma	18.5 ≤ BMI < 25	3848 / 137041 (2.81%)	-	
	25 ≤ BMI < 30	5950 / 172714 (3.45%)	1.23 (1.18-1.28)	<0.001
	BMI ≥ 30	4512 / 94004 (4.8%)	1.74 (1.66-1.81)	<0.001
Liver Disease	18.5 ≤ BMI < 25	812 / 137041 (0.59%)	-	
	25 ≤ BMI < 30	1536 / 172714 (0.89%)	1.51 (1.38-1.64)	<0.001
	BMI ≥ 30	1577 / 94004 (1.68%)	2.86 (2.63-3.11)	<0.001
Renal Failure	18.5 ≤ BMI < 25	1876 / 137041 (1.37%)	-	
	25 ≤ BMI < 30	3874 / 172714 (2.24%)	1.65 (1.56-1.74)	<0.001
	BMI ≥ 30	3674 / 94004 (3.91%)	2.9 (2.75-3.07)	<0.001
Gout	18.5 ≤ BMI < 25	165 / 137041 (0.12%)	-	
	25 ≤ BMI < 30	789 / 172714 (0.46%)	3.81 (3.22-4.5)	<0.001
	BMI ≥ 30	889 / 94004 (0.95%)	7.93 (6.72-9.37)	<0.001
Osteoarthritis	18.5 ≤ BMI < 25	5571 / 137041 (4.07%)	-	
	25 ≤ BMI < 30	11236 / 172714 (6.51%)	1.62 (1.57-1.68)	<0.001
	BMI ≥ 30	10359 / 94004 (11.02%)	2.83 (2.74-2.93)	<0.001
Sleep Disorders	18.5 ≤ BMI < 25	340 / 137041 (0.25%)	-	
	25 ≤ BMI < 30	932 / 172714 (0.54%)	2.18 (1.93-2.47)	<0.001
	BMI ≥ 30	1747 / 94004 (1.86%)	7.59 (6.75-8.52)	<0.001
Mood Disorders	18.5 ≤ BMI < 25	2700 / 137041 (1.97%)	-	
	25 ≤ BMI < 30	3878 / 172714 (2.25%)	1.14 (1.09-1.2)	<0.001
	BMI ≥ 30	3397 / 94004 (3.61%)	1.86 (1.77-1.96)	<0.001

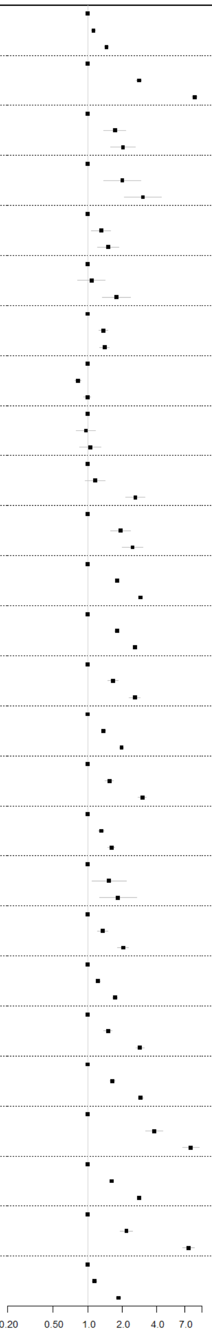

eFigure 7. Hazard Ratios for Incident Diseases by Body Mass Index Category After Exclusion of Participants With Diseases Diagnosed Within the First 2 Years of Follow-up

BMI, body mass index; HR, hazard ratio; CI, confidence interval

Hazard ratios (95% CI) for obesity (BMI ≥30.0 kg/m²) and overweight (25-29.9 kg/m²) versus healthy weight (18.5–24.9 kg/m²) were calculated in Cox proportional hazards models. All the p-values are presented after Bonferroni multiple testing adjustments.

Healthy lifestyle score included 4 lifestyle factors (never smoking, no or moderate alcohol consumption, physical activity, healthy diet). Individuals scored 1 if they met the criterion for a healthy lifestyle and 0 otherwise

© 2023 Rassy N et al. *JAMA Network Open*.

Disease	BMI	N (%) Cases	HR (95% CI)	Adjusted pvalue
Infections	18.5 ≤ BMI < 25	4058 / 137041 (2.96%)	-	
	25 ≤ BMI < 30	5725 / 172714 (3.31%)	1.06 (1.02-1.11)	0.024
	BMI ≥ 30	4023 / 94004 (4.28%)	1.34 (1.28-1.4)	<0.001
Diabete	18.5 ≤ BMI < 25	1426 / 137041 (1.04%)	-	
	25 ≤ BMI < 30	4983 / 172714 (2.89%)	2.37 (2.23-2.51)	<0.001
	BMI ≥ 30	8033 / 94004 (8.55%)	7.23 (6.83-7.65)	<0.001
Esophageal Adenocarcinoma	18.5 ≤ BMI < 25	113 / 137041 (0.08%)	-	
	25 ≤ BMI < 30	246 / 172714 (0.14%)	1.24 (0.99-1.56)	0.507
	BMI ≥ 30	157 / 94004 (0.17%)	1.47 (1.15-1.88)	0.017
Gastric Cardia Cancer	18.5 ≤ BMI < 25	40 / 137041 (0.03%)	-	
	25 ≤ BMI < 30	101 / 172714 (0.06%)	1.39 (0.96-2.02)	0.693
	BMI ≥ 30	83 / 94004 (0.09%)	2.28 (1.56-3.34)	<0.001
Pancreatic Cancer	18.5 ≤ BMI < 25	164 / 137041 (0.12%)	-	
	25 ≤ BMI < 30	271 / 172714 (0.16%)	1.13 (0.93-1.38)	1
	BMI ≥ 30	170 / 94004 (0.18%)	1.32 (1.06-1.64)	0.106
Liver Cancer	18.5 ≤ BMI < 25	89 / 137041 (0.06%)	-	
	25 ≤ BMI < 30	121 / 172714 (0.07%)	0.89 (0.68-1.18)	1
	BMI ≥ 30	108 / 94004 (0.11%)	1.49 (1.12-1.98)	0.051
Colorectal Cancer	18.5 ≤ BMI < 25	764 / 137041 (0.56%)	-	
	25 ≤ BMI < 30	1316 / 172714 (0.76%)	1.16 (1.06-1.27)	0.008
	BMI ≥ 30	732 / 94004 (0.78%)	1.23 (1.11-1.36)	<0.001
Breast Cancer	18.5 ≤ BMI < 25	1873 / 137041 (1.37%)	-	
	25 ≤ BMI < 30	1942 / 172714 (1.12%)	1.09 (1.02-1.16)	0.103
	BMI ≥ 30	1275 / 94004 (1.36%)	1.19 (1.11-1.28)	<0.001
Ovarian Cancer	18.5 ≤ BMI < 25	200 / 137041 (0.15%)	-	
	25 ≤ BMI < 30	243 / 172714 (0.14%)	1.22 (1.01-1.47)	0.348
	BMI ≥ 30	145 / 94004 (0.15%)	1.22 (0.98-1.51)	0.674
Endometrial Cancer	18.5 ≤ BMI < 25	168 / 137041 (0.12%)	-	
	25 ≤ BMI < 30	246 / 172714 (0.14%)	1.49 (1.22-1.81)	<0.001
	BMI ≥ 30	299 / 94004 (0.32%)	3.09 (2.55-3.73)	<0.001
Kidney Cancer	18.5 ≤ BMI < 25	136 / 137041 (0.1%)	-	
	25 ≤ BMI < 30	331 / 172714 (0.19%)	1.59 (1.3-1.94)	<0.001
	BMI ≥ 30	229 / 94004 (0.24%)	2.12 (1.71-2.63)	<0.001
Hypertension	18.5 ≤ BMI < 25	9818 / 137041 (7.16%)	-	
	25 ≤ BMI < 30	21693 / 172714 (12.56%)	1.59 (1.55-1.63)	<0.001
	BMI ≥ 30	18007 / 94004 (19.16%)	2.56 (2.5-2.62)	<0.001
Ischemic Heart Disease	18.5 ≤ BMI < 25	3948 / 137041 (2.88%)	-	
	25 ≤ BMI < 30	8843 / 172714 (5.12%)	1.4 (1.35-1.45)	<0.001
	BMI ≥ 30	6832 / 94004 (7.27%)	2.1 (2.01-2.18)	<0.001
Pulmonary Embolism	18.5 ≤ BMI < 25	495 / 137041 (0.36%)	-	
	25 ≤ BMI < 30	1040 / 172714 (0.6%)	1.46 (1.31-1.63)	<0.001
	BMI ≥ 30	871 / 94004 (0.93%)	2.28 (2.04-2.55)	<0.001
Arrhythmias	18.5 ≤ BMI < 25	3215 / 137041 (2.35%)	-	
	25 ≤ BMI < 30	5554 / 172714 (3.22%)	1.1 (1.06-1.15)	<0.001
	BMI ≥ 30	4302 / 94004 (4.58%)	1.66 (1.59-1.74)	<0.001
Heart Failure	18.5 ≤ BMI < 25	833 / 137041 (0.61%)	-	
	25 ≤ BMI < 30	1623 / 172714 (0.94%)	1.19 (1.09-1.29)	<0.001
	BMI ≥ 30	1699 / 94004 (1.81%)	2.34 (2.15-2.54)	<0.001
Cerebrovascular Diseases	18.5 ≤ BMI < 25	1521 / 137041 (1.11%)	-	
	25 ≤ BMI < 30	2506 / 172714 (1.45%)	1.07 (1-1.14)	0.321
	BMI ≥ 30	1672 / 94004 (1.78%)	1.32 (1.23-1.42)	<0.001
Arteriosclerosis	18.5 ≤ BMI < 25	50 / 137041 (0.04%)	-	
	25 ≤ BMI < 30	97 / 172714 (0.06%)	1.04 (0.74-1.47)	1
	BMI ≥ 30	63 / 94004 (0.07%)	1.17 (0.81-1.7)	1
Deep Vein Thrombosis	18.5 ≤ BMI < 25	533 / 137041 (0.39%)	-	
	25 ≤ BMI < 30	908 / 172714 (0.53%)	1.19 (1.07-1.33)	0.011
	BMI ≥ 30	741 / 94004 (0.79%)	1.82 (1.62-2.03)	<0.001
Asthma	18.5 ≤ BMI < 25	3848 / 137041 (2.81%)	-	
	25 ≤ BMI < 30	5950 / 172714 (3.45%)	1.25 (1.2-1.3)	<0.001
	BMI ≥ 30	4512 / 94004 (4.8%)	1.72 (1.65-1.8)	<0.001
Liver Disease	18.5 ≤ BMI < 25	812 / 137041 (0.59%)	-	
	25 ≤ BMI < 30	1536 / 172714 (0.89%)	1.36 (1.25-1.49)	<0.001
	BMI ≥ 30	1577 / 94004 (1.68%)	2.47 (2.27-2.69)	<0.001
Renal Failure	18.5 ≤ BMI < 25	1876 / 137041 (1.37%)	-	
	25 ≤ BMI < 30	3874 / 172714 (2.24%)	1.36 (1.29-1.44)	<0.001
	BMI ≥ 30	3674 / 94004 (3.91%)	2.4 (2.27-2.54)	<0.001
Gout	18.5 ≤ BMI < 25	165 / 137041 (0.12%)	-	
	25 ≤ BMI < 30	789 / 172714 (0.46%)	2.54 (2.14-3)	<0.001
	BMI ≥ 30	889 / 94004 (0.95%)	5.53 (4.67-6.53)	<0.001
Osteoarthritis	18.5 ≤ BMI < 25	5571 / 137041 (4.07%)	-	
	25 ≤ BMI < 30	11236 / 172714 (6.51%)	1.56 (1.51-1.61)	<0.001
	BMI ≥ 30	10359 / 94004 (11.02%)	2.72 (2.63-2.81)	<0.001
Sleep Disorders	18.5 ≤ BMI < 25	340 / 137041 (0.25%)	-	
	25 ≤ BMI < 30	932 / 172714 (0.54%)	1.86 (1.65-2.11)	<0.001
	BMI ≥ 30	1747 / 94004 (1.86%)	6.44 (5.73-7.24)	<0.001
Mood Disorders	18.5 ≤ BMI < 25	2700 / 137041 (1.97%)	-	
	25 ≤ BMI < 30	3878 / 172714 (2.25%)	1.21 (1.15-1.27)	<0.001
	BMI ≥ 30	3397 / 94004 (3.61%)	1.82 (1.73-1.91)	<0.001

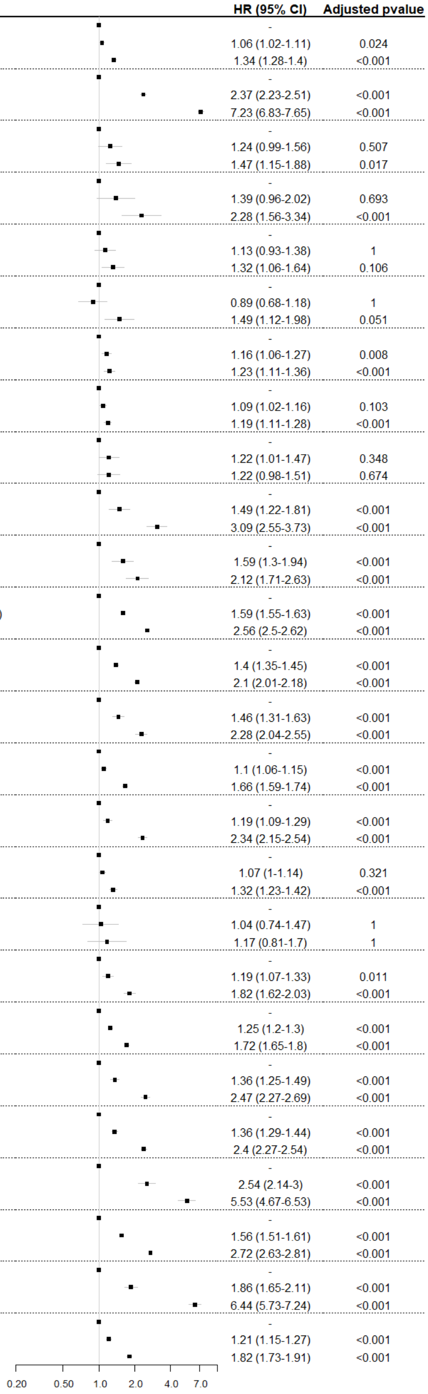

eFigure 8. Adjusted Hazard Ratios for Incident Diseases by Body Mass Index Category After Exclusion of Participants With Diseases Diagnosed Within the First 2 Years of Follow-up

BMI, body mass index; HR, hazard ratio; CI, confidence interval

Hazard ratios (95% CI) for obesity (BMI ≥30.0 kg/m²) and overweight (25-29.9 kg/m²) versus healthy weight (18.5–24.9 kg/m²) were calculated in Cox proportional hazards models after adjustment of age, sex, Townsend deprivation index, and healthy lifestyle score. All the p-values are presented after Bonferroni multiple testing adjustments.

Healthy lifestyle score included 4 lifestyle factors (never smoking, no or moderate alcohol consumption, physical activity, healthy diet). Individuals scored 1 if they met the criterion for a healthy lifestyle and 0 otherwise

© 2023 Rassy N et al. *JAMA Network Open*.

A.

Disease	Healthy lifestyle factors	N (%) Cases	HR (95% CI)	Adjusted pvalue
Infections	Zero	136 / 2942 (4.62%)	-	
	One	619 / 16517 (3.75%)	0.8 (0.67-0.96)	0.078
	Two	1297 / 41668 (3.11%)	0.66 (0.55-0.79)	<0.001
	Three	1336 / 48721 (2.74%)	0.58 (0.49-0.69)	<0.001
	Four	670 / 27193 (2.46%)	0.52 (0.43-0.63)	<0.001
Diabete	Zero	42 / 2942 (1.43%)	-	
	One	195 / 16517 (1.18%)	0.82 (0.59-1.15)	0.978
	Two	446 / 41668 (1.07%)	0.74 (0.54-1.02)	0.253
	Three	504 / 48721 (1.03%)	0.71 (0.52-0.98)	0.145
	Four	239 / 27193 (0.88%)	0.61 (0.44-0.84)	0.011
Esophageal Adenocarcinoma	Zero	6 / 2942 (0.2%)	-	
	One	21 / 16517 (0.13%)	0.62 (0.25-1.53)	1
	Two	45 / 41668 (0.11%)	0.52 (0.22-1.23)	0.547
	Three	26 / 48721 (0.05%)	0.26 (0.11-0.63)	0.011
	Four	15 / 27193 (0.06%)	0.27 (0.1-0.69)	0.025
Gastric Cardia Cancer	Zero	2 / 2942 (0.07%)	-	
	One	6 / 16517 (0.04%)	0.53 (0.11-2.63)	1
	Two	12 / 41668 (0.03%)	0.42 (0.09-1.87)	1
	Three	13 / 48721 (0.03%)	0.39 (0.09-1.71)	0.842
	Four	7 / 27193 (0.03%)	0.37 (0.08-1.79)	0.87
Pancreatic Cancer	Zero	4 / 2942 (0.14%)	-	
	One	29 / 16517 (0.18%)	1.28 (0.45-3.65)	1
	Two	53 / 41668 (0.13%)	0.93 (0.34-2.56)	1
	Three	48 / 48721 (0.1%)	0.72 (0.26-1.98)	1
	Four	30 / 27193 (0.11%)	0.8 (0.28-2.27)	1
Liver Cancer	Zero	5 / 2942 (0.17%)	-	
	One	18 / 16517 (0.11%)	0.64 (0.24-1.71)	1
	Two	24 / 41668 (0.06%)	0.33 (0.13-0.88)	0.104
	Three	33 / 48721 (0.07%)	0.39 (0.15-1.01)	0.206
	Four	9 / 27193 (0.03%)	0.19 (0.06-0.57)	0.012
Colorectal Cancer	Zero	12 / 2942 (0.41%)	-	
	One	108 / 16517 (0.65%)	1.6 (0.88-2.9)	0.499
	Two	244 / 41668 (0.59%)	1.42 (0.8-2.54)	0.933
	Three	262 / 48721 (0.54%)	1.3 (0.73-2.32)	1
	Four	138 / 27193 (0.51%)	1.23 (0.68-2.21)	1
Breast Cancer	Zero	30 / 2942 (1.02%)	-	
	One	237 / 16517 (1.43%)	1.4 (0.96-2.05)	0.326
	Two	596 / 41668 (1.43%)	1.39 (0.97-2.01)	0.307
	Three	662 / 48721 (1.36%)	1.32 (0.91-1.9)	0.553
	Four	348 / 27193 (1.28%)	1.24 (0.85-1.8)	1
Ovarian Cancer	Zero	2 / 2942 (0.07%)	-	
	One	18 / 16517 (0.11%)	1.59 (0.37-6.87)	1
	Two	59 / 41668 (0.14%)	2.06 (0.5-8.45)	1
	Three	78 / 48721 (0.16%)	2.33 (0.57-9.48)	0.952
	Four	43 / 27193 (0.16%)	2.3 (0.56-9.48)	1
Endometrial Cancer	Zero	0 / 2942 (0%)	-	
	One	17 / 16517 (0.1%)	2837019.78 (0-Inf)	1
	Two	41 / 41668 (0.1%)	2701769.23 (0-Inf)	1
	Three	71 / 48721 (0.15%)	3993159.48 (0-Inf)	1
	Four	39 / 27193 (0.14%)	3923323.98 (0-Inf)	1
Kidney Cancer	Zero	4 / 2942 (0.14%)	-	
	One	17 / 16517 (0.1%)	0.75 (0.25-2.23)	1
	Two	35 / 41668 (0.08%)	0.61 (0.22-1.72)	1
	Three	54 / 48721 (0.11%)	0.8 (0.29-2.22)	1
	Four	26 / 27193 (0.1%)	0.69 (0.24-1.98)	1
Hypertension	Zero	333 / 2942 (11.32%)	-	
	One	1486 / 16517 (9%)	0.78 (0.69-0.88)	<0.001
	Two	3045 / 41668 (7.31%)	0.63 (0.56-0.7)	<0.001
	Three	3210 / 48721 (6.59%)	0.56 (0.5-0.63)	<0.001
	Four	1744 / 27193 (6.41%)	0.55 (0.48-0.61)	<0.001
Ischemic Heart Disease	Zero	160 / 2942 (5.44%)	-	
	One	641 / 16517 (3.88%)	0.7 (0.59-0.84)	<0.001
	Two	1219 / 41668 (2.93%)	0.53 (0.45-0.62)	<0.001
	Three	1269 / 48721 (2.6%)	0.47 (0.4-0.55)	<0.001
	Four	659 / 27193 (2.42%)	0.43 (0.36-0.51)	<0.001
Pulmonary Embolism	Zero	29 / 2942 (0.99%)	-	
	One	73 / 16517 (0.44%)	0.44 (0.29-0.68)	<0.001
	Two	147 / 41668 (0.35%)	0.35 (0.24-0.53)	<0.001
	Three	161 / 48721 (0.33%)	0.33 (0.22-0.49)	<0.001
	Four	85 / 27193 (0.31%)	0.31 (0.2-0.48)	<0.001
Arrhythmias	Zero	99 / 2942 (3.37%)	-	
	One	447 / 16517 (2.71%)	0.8 (0.64-0.99)	0.167
	Two	964 / 41668 (2.31%)	0.68 (0.55-0.83)	<0.001
	Three	1087 / 48721 (2.23%)	0.65 (0.53-0.8)	<0.001
	Four	618 / 27193 (2.27%)	0.66 (0.54-0.82)	<0.001
Heart Failure	Zero	38 / 2942 (1.29%)	-	
	One	146 / 16517 (0.88%)	0.68 (0.47-0.97)	0.133
	Two	267 / 41668 (0.64%)	0.49 (0.35-0.69)	<0.001
	Three	246 / 48721 (0.5%)	0.38 (0.27-0.54)	<0.001
	Four	136 / 27193 (0.5%)	0.38 (0.27-0.54)	<0.001
Cerebrovascular Diseases	Zero	59 / 2942 (2.01%)	-	
	One	256 / 16517 (1.55%)	0.77 (0.58-1.02)	0.258
	Two	477 / 41668 (1.14%)	0.56 (0.43-0.74)	<0.001
	Three	450 / 48721 (0.92%)	0.45 (0.34-0.59)	<0.001
	Four	279 / 27193 (1.03%)	0.5 (0.38-0.66)	<0.001
Arteriosclerosis	Zero	8 / 2942 (0.27%)	-	
	One	15 / 16517 (0.09%)	0.33 (0.14-0.78)	0.047
	Two	18 / 41668 (0.04%)	0.16 (0.07-0.36)	<0.001
	Three	6 / 48721 (0.01%)	0.04 (0.02-0.13)	<0.001
	Four	3 / 27193 (0.01%)	0.04 (0.01-0.15)	<0.001
Deep Vein Thrombosis	Zero	24 / 2942 (0.82%)	-	
	One	81 / 16517 (0.49%)	0.6 (0.38-0.94)	0.106
	Two	170 / 41668 (0.41%)	0.49 (0.32-0.76)	0.005
	Three	155 / 48721 (0.32%)	0.38 (0.25-0.59)	<0.001
	Four	103 / 27193 (0.38%)	0.46 (0.29-0.71)	0.002
Asthma	Zero	120 / 2942 (4.08%)	-	
	One	533 / 16517 (3.23%)	0.78 (0.64-0.95)	0.059
	Two	1155 / 41668 (2.77%)	0.67 (0.55-0.81)	<0.001
	Three	1301 / 48721 (2.67%)	0.64 (0.53-0.77)	<0.001
	Four	739 / 27193 (2.72%)	0.65 (0.54-0.79)	<0.001
Liver Disease	Zero	40 / 2942 (1.36%)	-	
	One	178 / 16517 (1.08%)	0.79 (0.56-1.11)	0.671
	Two	267 / 41668 (0.64%)	0.46 (0.33-0.65)	<0.001
	Three	218 / 48721 (0.45%)	0.32 (0.23-0.45)	<0.001
	Four	109 / 27193 (0.4%)	0.29 (0.2-0.42)	<0.001
Renal Failure	Zero	71 / 2942 (2.41%)	-	
	One	321 / 16517 (1.94%)	0.8 (0.62-1.03)	0.336
	Two	581 / 41668 (1.39%)	0.57 (0.44-0.73)	<0.001
	Three	569 / 48721 (1.17%)	0.47 (0.37-0.61)	<0.001
	Four	334 / 27193 (1.23%)	0.5 (0.39-0.64)	<0.001
Gout	Zero	13 / 2942 (0.44%)	-	
	One	31 / 16517 (0.19%)	0.42 (0.22-0.8)	0.035
	Two	55 / 41668 (0.13%)	0.29 (0.16-0.54)	<0.001
	Three	49 / 48721 (0.1%)	0.22 (0.12-0.41)	<0.001
	Four	17 / 27193 (0.06%)	0.14 (0.07-0.29)	<0.001
Osteoarthritis	Zero	103 / 2942 (3.5%)	-	
	One	718 / 16517 (4.35%)	1.24 (1.01-1.52)	0.17
	Two	1736 / 41668 (4.17%)	1.18 (0.97-1.44)	0.393
	Three	1988 / 48721 (4.08%)	1.15 (0.95-1.41)	0.621
	Four	1026 / 27193 (3.77%)	1.06 (0.87-1.3)	1
Sleep Disorders	Zero	13 / 2942 (0.44%)	-	
	One	53 / 16517 (0.32%)	0.72 (0.39-1.32)	1
	Two	104 / 41668 (0.25%)	0.56 (0.31-0.99)	0.19
	Three	108 / 48721 (0.22%)	0.49 (0.28-0.88)	0.066
	Four	62 / 27193 (0.23%)	0.51 (0.28-0.92)	0.106
Mood Disorders	Zero	94 / 2942 (3.2%)	-	
	One	471 / 16517 (2.85%)	0.88 (0.71-1.1)	1
	Two	867 / 41668 (2.08%)	0.64 (0.52-0.79)	<0.001
	Three	864 / 48721 (1.77%)	0.54 (0.44-0.67)	<0.001
	Four	404 / 27193 (1.49%)	0.45 (0.36-0.57)	<0.001

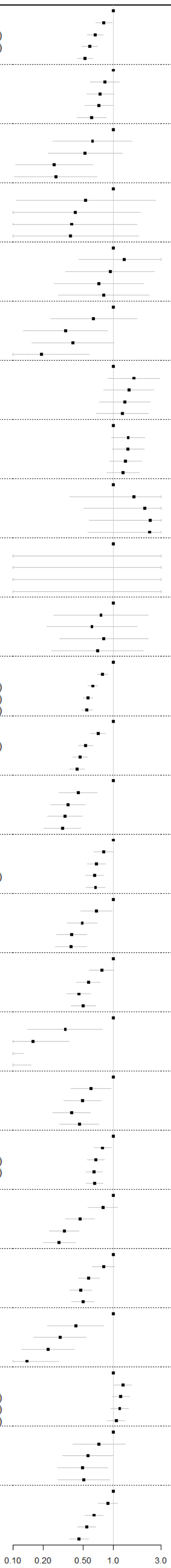

© 2023 Rassy N et al. *JAMA Network Open*.

B.

Disease	Healthy lifestyle factors	N (%) Cases	HR (95% CI)	Adjusted pvalue
Infections	Zero	210 / 5297 (3.96%)	-	
	One	941 / 26365 (3.57%)	0.9 (0.77-1.04)	0.625
	Two	1905 / 56202 (3.39%)	0.85 (0.74-0.98)	0.095
	Three	1812 / 57226 (3.17%)	0.79 (0.68-0.91)	0.005
	Four	857 / 27624 (3.1%)	0.77 (0.66-0.9)	0.003
Diabete	Zero	194 / 5297 (3.66%)	-	
	One	916 / 26365 (3.47%)	0.95 (0.81-1.1)	1
	Two	1633 / 56202 (2.91%)	0.79 (0.68-0.91)	0.006
	Three	1565 / 57226 (2.73%)	0.74 (0.64-0.86)	<0.001
	Four	675 / 27624 (2.44%)	0.66 (0.56-0.77)	<0.001
Esophageal Adenocarcinoma	Zero	17 / 5297 (0.32%)	-	
	One	64 / 26365 (0.24%)	0.76 (0.44-1.29)	1
	Two	91 / 56202 (0.16%)	0.5 (0.3-0.84)	0.036
	Three	56 / 57226 (0.1%)	0.3 (0.18-0.52)	<0.001
	Four	18 / 27624 (0.07%)	0.2 (0.1-0.39)	<0.001
Gastric Cardia Cancer	Zero	4 / 5297 (0.08%)	-	
	One	22 / 26365 (0.08%)	1.1 (0.38-3.2)	1
	Two	44 / 56202 (0.08%)	1.03 (0.37-2.87)	1
	Three	25 / 57226 (0.04%)	0.57 (0.2-1.65)	1
	Four	6 / 27624 (0.02%)	0.29 (0.08-1.01)	0.208
Pancreatic Cancer	Zero	8 / 5297 (0.15%)	-	
	One	44 / 26365 (0.17%)	1.1 (0.52-2.34)	1
	Two	98 / 56202 (0.17%)	1.15 (0.56-2.36)	1
	Three	84 / 57226 (0.15%)	0.96 (0.47-1.99)	1
	Four	37 / 27624 (0.13%)	0.88 (0.41-1.89)	1
Liver Cancer	Zero	6 / 5297 (0.11%)	-	
	One	27 / 26365 (0.1%)	0.9 (0.37-2.19)	1
	Two	37 / 56202 (0.07%)	0.58 (0.24-1.37)	0.851
	Three	31 / 57226 (0.05%)	0.47 (0.2-1.14)	0.378
	Four	20 / 27624 (0.07%)	0.63 (0.25-1.58)	1
Colorectal Cancer	Zero	40 / 5297 (0.76%)	-	
	One	274 / 26365 (1.04%)	1.38 (0.99-1.92)	0.236
	Two	433 / 56202 (0.77%)	1.02 (0.73-1.4)	1
	Three	390 / 57226 (0.68%)	0.9 (0.65-1.24)	1
	Four	179 / 27624 (0.65%)	0.85 (0.6-1.2)	1
Breast Cancer	Zero	48 / 5297 (0.91%)	-	
	One	248 / 26365 (0.94%)	1.04 (0.76-1.41)	1
	Two	652 / 56202 (1.16%)	1.28 (0.95-1.71)	0.414
	Three	669 / 57226 (1.17%)	1.28 (0.96-1.72)	0.383
	Four	325 / 27624 (1.18%)	1.29 (0.95-1.75)	0.399
Ovarian Cancer	Zero	11 / 5297 (0.21%)	-	
	One	33 / 26365 (0.13%)	0.6 (0.3-1.19)	0.58
	Two	74 / 56202 (0.13%)	0.63 (0.33-1.19)	0.616
	Three	87 / 57226 (0.15%)	0.73 (0.39-1.36)	1
	Four	38 / 27624 (0.14%)	0.66 (0.34-1.28)	0.877
Endometrial Cancer	Zero	2 / 5297 (0.04%)	-	
	One	30 / 26365 (0.11%)	3.01 (0.72-12.6)	0.524
	Two	68 / 56202 (0.12%)	3.19 (0.78-13.02)	0.424
	Three	93 / 57226 (0.16%)	4.28 (1.05-17.35)	0.168
	Four	53 / 27624 (0.19%)	5.04 (1.23-20.69)	0.099
Kidney Cancer	Zero	5 / 5297 (0.09%)	-	
	One	60 / 26365 (0.23%)	2.41 (0.97-6)	0.236
	Two	128 / 56202 (0.23%)	2.4 (0.98-5.87)	0.218
	Three	96 / 57226 (0.17%)	1.76 (0.72-4.33)	0.864
	Four	42 / 27624 (0.15%)	1.6 (0.63-4.04)	1
Hypertension	Zero	835 / 5297 (15.76%)	-	
	One	3701 / 26365 (14.04%)	0.88 (0.82-0.95)	0.003
	Two	7203 / 56202 (12.82%)	0.8 (0.74-0.85)	<0.001
	Three	6740 / 57226 (11.78%)	0.72 (0.67-0.78)	<0.001
	Four	3214 / 27624 (11.63%)	0.71 (0.66-0.77)	<0.001
Ischemic Heart Disease	Zero	363 / 5297 (6.85%)	-	
	One	1642 / 26365 (6.23%)	0.91 (0.81-1.01)	0.344
	Two	3010 / 56202 (5.36%)	0.77 (0.69-0.86)	<0.001
	Three	2650 / 57226 (4.63%)	0.66 (0.6-0.74)	<0.001
	Four	1178 / 27624 (4.26%)	0.61 (0.54-0.69)	<0.001
Pulmonary Embolism	Zero	33 / 5297 (0.62%)	-	
	One	161 / 26365 (0.61%)	0.98 (0.67-1.42)	1
	Two	366 / 56202 (0.65%)	1.04 (0.73-1.48)	1
	Three	314 / 57226 (0.55%)	0.87 (0.61-1.25)	1
	Four	166 / 27624 (0.6%)	0.96 (0.66-1.39)	1
Arrhythmias	Zero	203 / 5297 (3.83%)	-	
	One	979 / 26365 (3.71%)	0.97 (0.83-1.13)	1
	Two	1907 / 56202 (3.39%)	0.88 (0.76-1.02)	0.334
	Three	1718 / 57226 (3%)	0.78 (0.67-0.9)	0.002
	Four	747 / 27624 (2.7%)	0.7 (0.6-0.81)	<0.001
Heart Failure	Zero	71 / 5297 (1.34%)	-	
	One	356 / 26365 (1.35%)	1.01 (0.78-1.3)	1
	Two	538 / 56202 (0.96%)	0.71 (0.55-0.91)	0.026
	Three	451 / 57226 (0.79%)	0.58 (0.45-0.75)	<0.001
	Four	207 / 27624 (0.75%)	0.55 (0.42-0.72)	<0.001
Cerebrovascular Diseases	Zero	123 / 5297 (2.32%)	-	
	One	486 / 26365 (1.84%)	0.79 (0.65-0.96)	0.081
	Two	822 / 56202 (1.46%)	0.62 (0.52-0.75)	<0.001
	Three	740 / 57226 (1.29%)	0.55 (0.45-0.67)	<0.001
	Four	335 / 27624 (1.21%)	0.51 (0.42-0.63)	<0.001
Arteriosclerosis	Zero	2 / 5297 (0.04%)	-	
	One	37 / 26365 (0.14%)	3.72 (0.9-15.42)	0.282
	Two	35 / 56202 (0.06%)	1.64 (0.4-6.84)	1
	Three	20 / 57226 (0.03%)	0.92 (0.22-3.94)	1
	Four	3 / 27624 (0.01%)	0.29 (0.05-1.71)	0.682
Deep Vein Thrombosis	Zero	33 / 5297 (0.62%)	-	
	One	142 / 26365 (0.54%)	0.86 (0.59-1.26)	1
	Two	298 / 56202 (0.53%)	0.85 (0.59-1.21)	1
	Three	279 / 57226 (0.49%)	0.78 (0.54-1.11)	0.67
	Four	156 / 27624 (0.56%)	0.9 (0.62-1.31)	1
Asthma	Zero	189 / 5297 (3.57%)	-	
	One	918 / 26365 (3.48%)	0.97 (0.83-1.14)	1
	Two	1921 / 56202 (3.42%)	0.95 (0.82-1.11)	1
	Three	1973 / 57226 (3.45%)	0.96 (0.83-1.11)	1
	Four	949 / 27624 (3.44%)	0.95 (0.82-1.12)	1
Liver Disease	Zero	74 / 5297 (1.4%)	-	
	One	311 / 26365 (1.18%)	0.84 (0.65-1.09)	0.738
	Two	498 / 56202 (0.89%)	0.63 (0.49-0.8)	<0.001
	Three	455 / 57226 (0.8%)	0.56 (0.44-0.72)	<0.001
	Four	198 / 27624 (0.72%)	0.51 (0.39-0.66)	<0.001
Renal Failure	Zero	154 / 5297 (2.91%)	-	
	One	735 / 26365 (2.79%)	0.96 (0.8-1.14)	1
	Two	1292 / 56202 (2.3%)	0.78 (0.66-0.93)	0.018
	Three	1168 / 57226 (2.04%)	0.69 (0.59-0.82)	<0.001
	Four	525 / 27624 (1.9%)	0.64 (0.54-0.77)	<0.001
Gout	Zero	53 / 5297 (1%)	-	
	One	189 / 26365 (0.72%)	0.71 (0.53-0.97)	0.122
	Two	278 / 56202 (0.49%)	0.49 (0.37-0.66)	<0.001
	Three	203 / 57226 (0.35%)	0.35 (0.26-0.47)	<0.001
	Four	66 / 27624 (0.24%)	0.24 (0.16-0.34)	<0.001
Osteoarthritis	Zero	340 / 5297 (6.42%)	-	
	One	1743 / 26365 (6.61%)	1.03 (0.92-1.16)	1
	Two	3716 / 56202 (6.61%)	1.02 (0.92-1.15)	1
	Three	3724 / 57226 (6.51%)	1.01 (0.9-1.12)	1
	Four	1713 / 27624 (6.2%)	0.96 (0.85-1.07)	1
Sleep Disorders	Zero	37 / 5297 (0.7%)	-	
	One	179 / 26365 (0.68%)	0.97 (0.68-1.38)	1
	Two	323 / 56202 (0.57%)	0.82 (0.58-1.15)	0.992
	Three	271 / 57226 (0.47%)	0.67 (0.48-0.95)	0.094
	Four	122 / 27624 (0.44%)	0.63 (0.43-0.9)	0.05
Mood Disorders	Zero	152 / 5297 (2.87%)	-	
	One	686 / 26365 (2.6%)	0.91 (0.76-1.08)	1
	Two	1308 / 56202 (2.33%)	0.81 (0.68-0.95)	0.046
	Three	1188 / 57226 (2.08%)	0.72 (0.6-0.85)	<0.001
	Four	544 / 27624 (1.97%)	0.68 (0.57-0.81)	<0.001

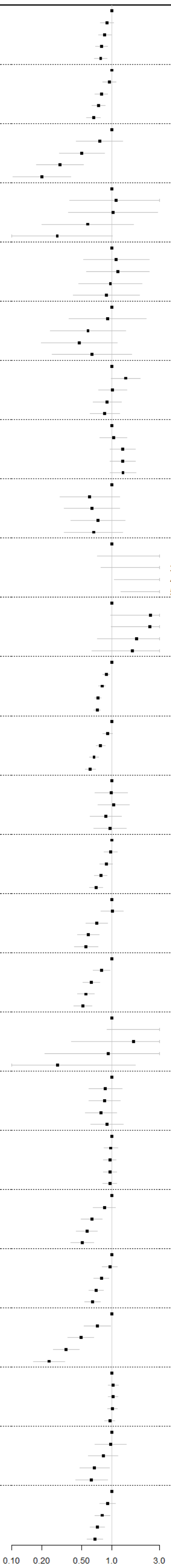

© 2023 Rassy N et al. *JAMA Network Open*.

C.

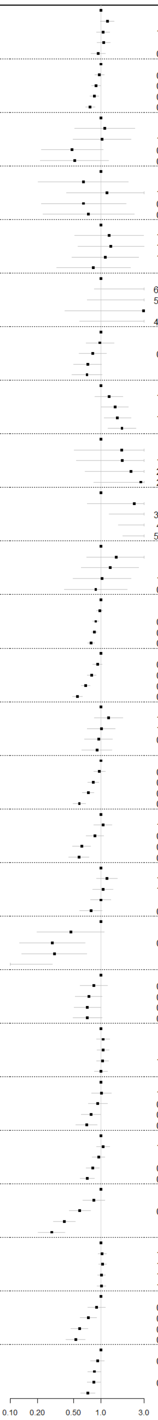

Disease	Healthy lifestyle factors	N (%) Cases	HR (95% CI)	Adjusted pvalue
Infections	Zero	154 / 3842 (4.01%)	-	
	One	779 / 16486 (4.73%)	1.17 (0.99-1.4)	0.27
	Two	1322 / 31114 (4.25%)	1.05 (0.89-1.24)	1
	Three	1274 / 29556 (4.31%)	1.07 (0.9-1.26)	1
	Four	494 / 13006 (3.8%)	0.94 (0.78-1.12)	1
Diabete	Zero	371 / 3842 (9.66%)	-	
	One	1543 / 16486 (9.36%)	0.96 (0.86-1.08)	1
	Two	2682 / 31114 (8.62%)	0.88 (0.79-0.98)	0.093
	Three	2464 / 29556 (8.34%)	0.85 (0.76-0.95)	0.014
	Four	973 / 13006 (7.48%)	0.76 (0.67-0.86)	<0.001
Esophageal Adenocarcinoma	Zero	8 / 3842 (0.21%)	-	
	One	38 / 16486 (0.23%)	1.1 (0.51-2.36)	1
	Two	67 / 31114 (0.22%)	1.03 (0.49-2.14)	1
	Three	30 / 29556 (0.1%)	0.48 (0.22-1.05)	0.268
	Four	14 / 13006 (0.11%)	0.51 (0.21-1.22)	0.519
Gastric Cardia Cancer	Zero	4 / 3842 (0.1%)	-	
	One	11 / 16486 (0.07%)	0.64 (0.2-2)	1
	Two	38 / 31114 (0.12%)	1.17 (0.42-3.26)	1
	Three	20 / 29556 (0.07%)	0.64 (0.22-1.88)	1
	Four	10 / 13006 (0.08%)	0.73 (0.23-2.33)	1
Pancreatic Cancer	Zero	6 / 3842 (0.16%)	-	
	One	32 / 16486 (0.19%)	1.24 (0.52-2.96)	1
	Two	63 / 31114 (0.2%)	1.29 (0.56-2.97)	1
	Three	52 / 29556 (0.18%)	1.11 (0.48-2.59)	1
	Four	17 / 13006 (0.13%)	0.83 (0.33-2.1)	1
Liver Cancer	Zero	1 / 3842 (0.03%)	-	
	One	27 / 16486 (0.16%)	6.26 (0.85-46.04)	0.287
	Two	42 / 31114 (0.13%)	5.15 (0.71-37.42)	0.421
	Three	23 / 29556 (0.08%)	2.96 (0.4-21.9)	1
	Four	15 / 13006 (0.12%)	4.38 (0.58-33.15)	0.611
Colorectal Cancer	Zero	37 / 3842 (0.96%)	-	
	One	156 / 16486 (0.95%)	0.98 (0.68-1.4)	1
	Two	245 / 31114 (0.79%)	0.81 (0.57-1.15)	0.94
	Three	205 / 29556 (0.69%)	0.71 (0.5-1.01)	0.228
	Four	89 / 13006 (0.68%)	0.7 (0.48-1.03)	0.281
Breast Cancer	Zero	36 / 3842 (0.94%)	-	
	One	190 / 16486 (1.15%)	1.22 (0.86-1.75)	1
	Two	417 / 31114 (1.34%)	1.42 (1.01-2)	0.169
	Three	423 / 29556 (1.43%)	1.52 (1.08-2.13)	0.067
	Four	209 / 13006 (1.61%)	1.7 (1.2-2.42)	0.013
Ovarian Cancer	Zero	3 / 3842 (0.08%)	-	
	One	22 / 16486 (0.13%)	1.7 (0.51-5.68)	1
	Two	42 / 31114 (0.13%)	1.72 (0.53-5.54)	1
	Three	50 / 29556 (0.17%)	2.14 (0.67-6.87)	0.798
	Four	28 / 13006 (0.22%)	2.73 (0.83-8.97)	0.395
Endometrial Cancer	Zero	3 / 3842 (0.08%)	-	
	One	30 / 16486 (0.18%)	2.32 (0.71-7.6)	0.659
	Two	95 / 31114 (0.31%)	3.89 (1.23-12.27)	0.082
	Three	114 / 29556 (0.39%)	4.9 (1.56-15.41)	0.026
	Four	57 / 13006 (0.44%)	5.56 (1.74-17.75)	0.015
Kidney Cancer	Zero	8 / 3842 (0.21%)	-	
	One	51 / 16486 (0.31%)	1.48 (0.7-3.11)	1
	Two	82 / 31114 (0.26%)	1.26 (0.61-2.6)	1
	Three	64 / 29556 (0.22%)	1.03 (0.49-2.15)	1
	Four	24 / 13006 (0.18%)	0.88 (0.39-1.95)	1
Hypertension	Zero	827 / 3842 (21.53%)	-	
	One	3460 / 16486 (20.99%)	0.97 (0.9-1.05)	1
	Two	5971 / 31114 (19.19%)	0.88 (0.81-0.94)	0.001
	Three	5496 / 29556 (18.6%)	0.84 (0.78-0.91)	<0.001
	Four	2253 / 13006 (17.32%)	0.78 (0.72-0.85)	<0.001
Ischemic Heart Disease	Zero	365 / 3842 (9.5%)	-	
	One	1442 / 16486 (8.75%)	0.91 (0.82-1.03)	0.505
	Two	2377 / 31114 (7.64%)	0.79 (0.71-0.88)	<0.001
	Three	1947 / 29556 (6.59%)	0.68 (0.61-0.76)	<0.001
	Four	701 / 13006 (5.39%)	0.55 (0.49-0.63)	<0.001
Pulmonary Embolism	Zero	35 / 3842 (0.91%)	-	
	One	183 / 16486 (1.11%)	1.21 (0.84-1.74)	1
	Two	288 / 31114 (0.93%)	1.01 (0.71-1.43)	1
	Three	257 / 29556 (0.87%)	0.94 (0.66-1.34)	1
	Four	108 / 13006 (0.83%)	0.9 (0.62-1.32)	1
Arrhythmias	Zero	220 / 3842 (5.73%)	-	
	One	914 / 16486 (5.54%)	0.96 (0.83-1.11)	1
	Two	1484 / 31114 (4.77%)	0.82 (0.71-0.95)	0.028
	Three	1244 / 29556 (4.21%)	0.72 (0.63-0.83)	<0.001
	Four	440 / 13006 (3.38%)	0.58 (0.49-0.68)	<0.001
Heart Failure	Zero	88 / 3842 (2.29%)	-	
	One	399 / 16486 (2.42%)	1.05 (0.83-1.32)	1
	Two	617 / 31114 (1.98%)	0.86 (0.69-1.07)	0.711
	Three	422 / 29556 (1.43%)	0.61 (0.49-0.77)	<0.001
	Four	173 / 13006 (1.33%)	0.57 (0.44-0.74)	<0.001
Cerebrovascular Diseases	Zero	67 / 3842 (1.74%)	-	
	One	335 / 16486 (2.03%)	1.16 (0.89-1.51)	1
	Two	574 / 31114 (1.84%)	1.05 (0.82-1.35)	1
	Three	518 / 29556 (1.75%)	1 (0.77-1.28)	1
	Four	178 / 13006 (1.37%)	0.78 (0.59-1.03)	0.303
Arteriosclerosis	Zero	8 / 3842 (0.21%)	-	
	One	16 / 16486 (0.1%)	0.46 (0.2-1.08)	0.304
	Two	19 / 31114 (0.06%)	0.29 (0.13-0.67)	0.014
	Three	19 / 29556 (0.06%)	0.31 (0.13-0.7)	0.02
	Four	1 / 13006 (0.01%)	0.04 (0-0.29)	0.007
Deep Vein Thrombosis	Zero	40 / 3842 (1.04%)	-	
	One	144 / 16486 (0.87%)	0.83 (0.59-1.18)	1
	Two	239 / 31114 (0.77%)	0.73 (0.52-1.02)	0.271
	Three	221 / 29556 (0.75%)	0.71 (0.51-0.99)	0.185
	Four	97 / 13006 (0.75%)	0.71 (0.49-1.02)	0.263
Asthma	Zero	176 / 3842 (4.58%)	-	
	One	800 / 16486 (4.85%)	1.06 (0.9-1.24)	1
	Two	1512 / 31114 (4.86%)	1.05 (0.9-1.23)	1
	Three	1421 / 29556 (4.81%)	1.04 (0.89-1.22)	1
	Four	603 / 13006 (4.64%)	1 (0.85-1.19)	1
Liver Disease	Zero	74 / 3842 (1.93%)	-	
	One	324 / 16486 (1.97%)	1.01 (0.79-1.31)	1
	Two	557 / 31114 (1.79%)	0.92 (0.72-1.18)	1
	Three	446 / 29556 (1.51%)	0.77 (0.61-0.99)	0.164
	Four	176 / 13006 (1.35%)	0.69 (0.53-0.91)	0.032
Renal Failure	Zero	167 / 3842 (4.35%)	-	
	One	760 / 16486 (4.61%)	1.06 (0.89-1.25)	1
	Two	1283 / 31114 (4.12%)	0.94 (0.8-1.11)	1
	Three	1057 / 29556 (3.58%)	0.81 (0.69-0.96)	0.05
	Four	407 / 13006 (3.13%)	0.71 (0.59-0.85)	<0.001
Gout	Zero	66 / 3842 (1.72%)	-	
	One	238 / 16486 (1.44%)	0.83 (0.64-1.1)	0.776
	Two	316 / 31114 (1.02%)	0.59 (0.45-0.76)	<0.001
	Three	204 / 29556 (0.69%)	0.4 (0.3-0.52)	<0.001
	Four	65 / 13006 (0.5%)	0.29 (0.2-0.4)	<0.001
Osteoarthritis	Zero	411 / 3842 (10.7%)	-	
	One	1824 / 16486 (11.06%)	1.03 (0.93-1.15)	1
	Two	3475 / 31114 (11.17%)	1.04 (0.94-1.15)	1
	Three	3225 / 29556 (10.91%)	1.01 (0.91-1.12)	1
	Four	1424 / 13006 (10.95%)	1.02 (0.91-1.13)	1
Sleep Disorders	Zero	102 / 3842 (2.65%)	-	
	One	394 / 16486 (2.39%)	0.89 (0.72-1.11)	1
	Two	604 / 31114 (1.94%)	0.72 (0.59-0.89)	0.01
	Three	462 / 29556 (1.56%)	0.58 (0.47-0.72)	<0.001
	Four	185 / 13006 (1.42%)	0.53 (0.41-0.67)	<0.001
Mood Disorders	Zero	163 / 3842 (4.24%)	-	
	One	643 / 16486 (3.9%)	0.91 (0.77-1.08)	1
	Two	1125 / 31114 (3.62%)	0.85 (0.72-1)	0.18
	Three	1063 / 29556 (3.6%)	0.84 (0.71-0.99)	0.142
	Four	403 / 13006 (3.1%)	0.72 (0.6-0.86)	0.002

eFigure 9. Hazard Ratios for Incident Diseases by Lifestyle Profile in Individuals With Normal Weight (BMI 18.5-24.9) (A), Overweight (BMI 25-29.9) (B), and Obesity (BMI ≥30) (C) After Exclusion of Participants With Diseases Diagnosed Within the First 2 years of Follow-up

BMI, body mass index; HR, hazard ratio; CI, confidence interval

Hazard ratios (95% CI) for a lifestyle score equal to 1, 2, 3 or 4 versus a lifestyle score equal to zero were calculated in Cox proportional hazards models. All the p-values are presented after Bonferroni multiple testing adjustments.

For each lifestyle factor, individuals scored 1 if they met the criterion for a healthy lifestyle (never smoking, no or moderate alcohol consumption, physical activity, or healthy diet) and 0 otherwise. The healthy lifestyle score was computed by summing the four scores. It ranged between zero (lowest healthy score) and 4 (highest healthy score).

© 2023 Rassy N et al. *JAMA Network Open*.

A.

Disease	Healthy lifestyle factors	N (%) Cases	HR (95% CI)	Adjusted pvalue
Infections	Zero	136 / 2942 (4.62%)	-	
	One	619 / 16517 (3.75%)	0.81 (0.67-0.97)	0.162
	Two	1297 / 41668 (3.11%)	0.67 (0.56-0.8)	<0.001
	Three	1336 / 48721 (2.74%)	0.59 (0.5-0.71)	<0.001
	Four	670 / 27193 (2.46%)	0.53 (0.44-0.64)	<0.001
Diabete	Zero	42 / 2942 (1.43%)	-	
	One	195 / 16517 (1.18%)	0.89 (0.63-1.24)	1
	Two	446 / 41668 (1.07%)	0.87 (0.63-1.2)	1
	Three	504 / 48721 (1.03%)	0.87 (0.63-1.19)	1
	Four	239 / 27193 (0.88%)	0.74 (0.53-1.02)	0.477
Esophageal Adenocarcinoma	Zero	6 / 2942 (0.2%)	-	
	One	21 / 16517 (0.13%)	0.65 (0.26-1.61)	1
	Two	45 / 41668 (0.11%)	0.59 (0.25-1.38)	1
	Three	26 / 48721 (0.05%)	0.3 (0.12-0.72)	0.053
	Four	15 / 27193 (0.06%)	0.3 (0.12-0.78)	0.096
Gastric Cardia Cancer	Zero	2 / 2942 (0.07%)	-	
	One	6 / 16517 (0.04%)	0.57 (0.11-2.81)	1
	Two	12 / 41668 (0.03%)	0.49 (0.11-2.21)	1
	Three	13 / 48721 (0.03%)	0.46 (0.1-2.05)	1
	Four	7 / 27193 (0.03%)	0.44 (0.09-2.14)	1
Pancreatic Cancer	Zero	4 / 2942 (0.14%)	-	
	One	29 / 16517 (0.18%)	1.3 (0.46-3.71)	1
	Two	53 / 41668 (0.13%)	0.97 (0.35-2.68)	1
	Three	48 / 48721 (0.1%)	0.75 (0.27-2.09)	1
	Four	30 / 27193 (0.11%)	0.83 (0.29-2.36)	1
Liver Cancer	Zero	5 / 2942 (0.17%)	-	
	One	18 / 16517 (0.11%)	0.63 (0.24-1.71)	1
	Two	24 / 41668 (0.06%)	0.34 (0.13-0.89)	0.193
	Three	33 / 48721 (0.07%)	0.4 (0.15-1.02)	0.382
	Four	9 / 27193 (0.03%)	0.19 (0.06-0.57)	0.021
Colorectal Cancer	Zero	12 / 2942 (0.41%)	-	
	One	108 / 16517 (0.65%)	1.59 (0.87-2.88)	0.911
	Two	244 / 41668 (0.59%)	1.42 (0.8-2.54)	1
	Three	262 / 48721 (0.54%)	1.29 (0.72-2.31)	1
	Four	138 / 27193 (0.51%)	1.2 (0.66-2.16)	1
Breast Cancer	Zero	30 / 2942 (1.02%)	-	
	One	237 / 16517 (1.43%)	1.22 (0.84-1.79)	1
	Two	596 / 41668 (1.43%)	1.09 (0.76-1.57)	1
	Three	662 / 48721 (1.36%)	1.01 (0.7-1.46)	1
	Four	348 / 27193 (1.28%)	0.94 (0.65-1.36)	1
Ovarian Cancer	Zero	2 / 2942 (0.07%)	-	
	One	18 / 16517 (0.11%)	1.37 (0.32-5.9)	1
	Two	59 / 41668 (0.14%)	1.56 (0.38-6.4)	1
	Three	78 / 48721 (0.16%)	1.73 (0.43-7.05)	1
	Four	43 / 27193 (0.16%)	1.65 (0.4-6.83)	1
Endometrial Cancer	Zero	0 / 2942 (0%)	-	
	One	17 / 16517 (0.1%)	45225002.97 (0-Inf)	1
	Two	41 / 41668 (0.1%)	37860290.34 (0-Inf)	1
	Three	71 / 48721 (0.15%)	54628744.56 (0-Inf)	1
	Four	39 / 27193 (0.14%)	52026593.5 (0-Inf)	1
Kidney Cancer	Zero	4 / 2942 (0.14%)	-	
	One	17 / 16517 (0.1%)	0.76 (0.26-2.27)	1
	Two	35 / 41668 (0.08%)	0.64 (0.23-1.81)	1
	Three	54 / 48721 (0.11%)	0.84 (0.3-2.33)	1
	Four	26 / 27193 (0.1%)	0.71 (0.25-2.05)	1
Hypertension	Zero	333 / 2942 (11.32%)	-	
	One	1486 / 16517 (9%)	0.78 (0.69-0.88)	<0.001
	Two	3045 / 41668 (7.31%)	0.64 (0.57-0.71)	<0.001
	Three	3210 / 48721 (6.59%)	0.57 (0.51-0.64)	<0.001
	Four	1744 / 27193 (6.41%)	0.54 (0.48-0.61)	<0.001
Ischemic Heart Disease	Zero	160 / 2942 (5.44%)	-	
	One	641 / 16517 (3.88%)	0.74 (0.62-0.88)	0.004
	Two	1219 / 41668 (2.93%)	0.6 (0.51-0.71)	<0.001
	Three	1269 / 48721 (2.6%)	0.54 (0.46-0.63)	<0.001
	Four	659 / 27193 (2.42%)	0.49 (0.42-0.59)	<0.001
Pulmonary Embolism	Zero	29 / 2942 (0.99%)	-	
	One	73 / 16517 (0.44%)	0.46 (0.3-0.7)	0.003
	Two	147 / 41668 (0.35%)	0.38 (0.25-0.56)	<0.001
	Three	161 / 48721 (0.33%)	0.36 (0.24-0.53)	<0.001
	Four	85 / 27193 (0.31%)	0.33 (0.22-0.51)	<0.001
Arrhythmias	Zero	99 / 2942 (3.37%)	-	
	One	447 / 16517 (2.71%)	0.82 (0.66-1.02)	0.572
	Two	964 / 41668 (2.31%)	0.74 (0.6-0.91)	0.029
	Three	1087 / 48721 (2.23%)	0.71 (0.58-0.88)	0.01
	Four	618 / 27193 (2.27%)	0.72 (0.58-0.89)	0.015
Heart Failure	Zero	38 / 2942 (1.29%)	-	
	One	146 / 16517 (0.88%)	0.71 (0.5-1.02)	0.456
	Two	267 / 41668 (0.64%)	0.56 (0.39-0.78)	0.005
	Three	246 / 48721 (0.5%)	0.44 (0.32-0.63)	<0.001
	Four	136 / 27193 (0.5%)	0.43 (0.3-0.62)	<0.001
Cerebrovascular Diseases	Zero	59 / 2942 (2.01%)	-	
	One	256 / 16517 (1.55%)	0.79 (0.6-1.05)	0.776
	Two	477 / 41668 (1.14%)	0.62 (0.47-0.81)	0.003
	Three	450 / 48721 (0.92%)	0.5 (0.38-0.66)	<0.001
	Four	279 / 27193 (1.03%)	0.55 (0.42-0.73)	<0.001
Arteriosclerosis	Zero	8 / 2942 (0.27%)	-	
	One	15 / 16517 (0.09%)	0.38 (0.16-0.9)	0.189
	Two	18 / 41668 (0.04%)	0.21 (0.09-0.49)	0.002
	Three	6 / 48721 (0.01%)	0.06 (0.02-0.19)	<0.001
	Four	3 / 27193 (0.01%)	0.06 (0.02-0.22)	<0.001
Deep Vein Thrombosis	Zero	24 / 2942 (0.82%)	-	
	One	81 / 16517 (0.49%)	0.62 (0.39-0.98)	0.28
	Two	170 / 41668 (0.41%)	0.54 (0.35-0.83)	0.033
	Three	155 / 48721 (0.32%)	0.42 (0.27-0.65)	<0.001
	Four	103 / 27193 (0.38%)	0.5 (0.32-0.78)	0.017
Asthma	Zero	120 / 2942 (4.08%)	-	
	One	533 / 16517 (3.23%)	0.78 (0.64-0.95)	0.106
	Two	1155 / 41668 (2.77%)	0.67 (0.56-0.81)	<0.001
	Three	1301 / 48721 (2.67%)	0.65 (0.54-0.78)	<0.001
	Four	739 / 27193 (2.72%)	0.65 (0.54-0.79)	<0.001
Liver Disease	Zero	40 / 2942 (1.36%)	-	
	One	178 / 16517 (1.08%)	0.83 (0.59-1.17)	1
	Two	267 / 41668 (0.64%)	0.52 (0.37-0.72)	<0.001
	Three	218 / 48721 (0.45%)	0.37 (0.26-0.52)	<0.001
	Four	109 / 27193 (0.4%)	0.33 (0.23-0.48)	<0.001
Renal Failure	Zero	71 / 2942 (2.41%)	-	
	One	321 / 16517 (1.94%)	0.82 (0.64-1.07)	0.976
	Two	581 / 41668 (1.39%)	0.62 (0.48-0.79)	<0.001
	Three	569 / 48721 (1.17%)	0.52 (0.41-0.67)	<0.001
	Four	334 / 27193 (1.23%)	0.54 (0.42-0.7)	<0.001
Gout	Zero	13 / 2942 (0.44%)	-	
	One	31 / 16517 (0.19%)	0.47 (0.25-0.9)	0.158
	Two	55 / 41668 (0.13%)	0.38 (0.21-0.71)	0.014
	Three	49 / 48721 (0.1%)	0.3 (0.16-0.55)	<0.001
	Four	17 / 27193 (0.06%)	0.19 (0.09-0.39)	<0.001
Osteoarthritis	Zero	103 / 2942 (3.5%)	-	
	One	718 / 16517 (4.35%)	1.18 (0.96-1.45)	0.798
	Two	1736 / 41668 (4.17%)	1.09 (0.89-1.33)	1
	Three	1988 / 48721 (4.08%)	1.06 (0.87-1.29)	1
	Four	1026 / 27193 (3.77%)	0.95 (0.77-1.16)	1
Sleep Disorders	Zero	13 / 2942 (0.44%)	-	
	One	53 / 16517 (0.32%)	0.78 (0.43-1.43)	1
	Two	104 / 41668 (0.25%)	0.65 (0.37-1.16)	1
	Three	108 / 48721 (0.22%)	0.59 (0.33-1.06)	0.534
	Four	62 / 27193 (0.23%)	0.62 (0.34-1.12)	0.795
Mood Disorders	Zero	94 / 2942 (3.2%)	-	
	One	471 / 16517 (2.85%)	0.89 (0.71-1.11)	1
	Two	867 / 41668 (2.08%)	0.65 (0.52-0.8)	<0.001
	Three	864 / 48721 (1.77%)	0.56 (0.45-0.69)	<0.001
	Four	404 / 27193 (1.49%)	0.47 (0.37-0.59)	<0.001

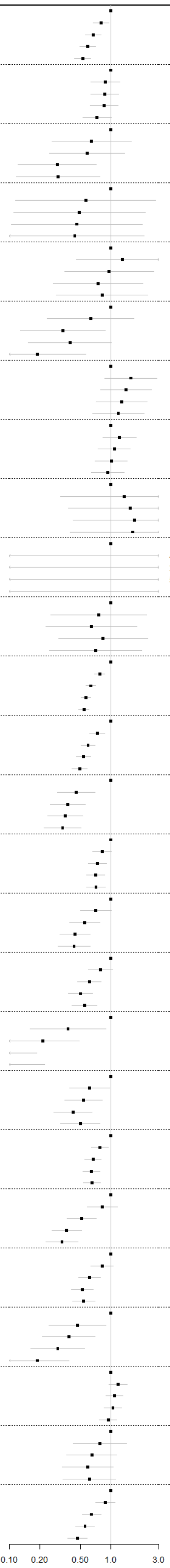

© 2023 Rassy N et al. *JAMA Network Open*.

B.

Disease	Healthy lifestyle factors	N (%) Cases	HR (95% CI)	Adjusted pvalue
Infections	Zero	210 / 5297 (3.96%)	-	
	One	941 / 26365 (3.57%)	0.88 (0.76-1.03)	0.716
	Two	1905 / 56202 (3.39%)	0.83 (0.72-0.96)	0.081
	Three	1812 / 57226 (3.17%)	0.77 (0.67-0.89)	0.003
	Four	857 / 27624 (3.1%)	0.75 (0.64-0.87)	0.001
Diabete	Zero	194 / 5297 (3.66%)	-	
	One	916 / 26365 (3.47%)	0.96 (0.82-1.12)	1
	Two	1633 / 56202 (2.91%)	0.84 (0.72-0.97)	0.141
	Three	1565 / 57226 (2.73%)	0.83 (0.71-0.96)	0.082
	Four	675 / 27624 (2.44%)	0.76 (0.65-0.89)	0.006
Esophageal Adenocarcinoma	Zero	17 / 5297 (0.32%)	-	
	One	64 / 26365 (0.24%)	0.78 (0.45-1.33)	1
	Two	91 / 56202 (0.16%)	0.55 (0.33-0.93)	0.178
	Three	56 / 57226 (0.1%)	0.36 (0.21-0.62)	0.002
	Four	18 / 27624 (0.07%)	0.25 (0.13-0.49)	<0.001
Gastric Cardia Cancer	Zero	4 / 5297 (0.08%)	-	
	One	22 / 26365 (0.08%)	1.16 (0.4-3.38)	1
	Two	44 / 56202 (0.08%)	1.2 (0.43-3.34)	1
	Three	25 / 57226 (0.04%)	0.74 (0.26-2.14)	1
	Four	6 / 27624 (0.02%)	0.4 (0.11-1.44)	1
Pancreatic Cancer	Zero	8 / 5297 (0.15%)	-	
	One	44 / 26365 (0.17%)	1.08 (0.51-2.3)	1
	Two	98 / 56202 (0.17%)	1.14 (0.55-2.34)	1
	Three	84 / 57226 (0.15%)	0.96 (0.47-1.99)	1
	Four	37 / 27624 (0.13%)	0.87 (0.41-1.88)	1
Liver Cancer	Zero	6 / 5297 (0.11%)	-	
	One	27 / 26365 (0.1%)	0.89 (0.37-2.15)	1
	Two	37 / 56202 (0.07%)	0.58 (0.24-1.37)	1
	Three	31 / 57226 (0.05%)	0.48 (0.2-1.15)	0.703
	Four	20 / 27624 (0.07%)	0.64 (0.26-1.6)	1
Colorectal Cancer	Zero	40 / 5297 (0.76%)	-	
	One	274 / 26365 (1.04%)	1.38 (0.99-1.92)	0.417
	Two	433 / 56202 (0.77%)	1.04 (0.75-1.44)	1
	Three	390 / 57226 (0.68%)	0.94 (0.68-1.3)	1
	Four	179 / 27624 (0.65%)	0.9 (0.64-1.28)	1
Breast Cancer	Zero	48 / 5297 (0.91%)	-	
	One	248 / 26365 (0.94%)	0.87 (0.64-1.18)	1
	Two	652 / 56202 (1.16%)	0.91 (0.68-1.22)	1
	Three	669 / 57226 (1.17%)	0.8 (0.6-1.08)	1
	Four	325 / 27624 (1.18%)	0.73 (0.54-0.99)	0.28
Ovarian Cancer	Zero	11 / 5297 (0.21%)	-	
	One	33 / 26365 (0.13%)	0.5 (0.25-0.98)	0.311
	Two	74 / 56202 (0.13%)	0.44 (0.23-0.83)	0.077
	Three	87 / 57226 (0.15%)	0.44 (0.24-0.83)	0.074
	Four	38 / 27624 (0.14%)	0.36 (0.18-0.7)	0.018
Endometrial Cancer	Zero	2 / 5297 (0.04%)	-	
	One	30 / 26365 (0.11%)	2.46 (0.59-10.28)	1
	Two	68 / 56202 (0.12%)	2.18 (0.53-8.89)	1
	Three	93 / 57226 (0.16%)	2.53 (0.62-10.27)	1
	Four	53 / 27624 (0.19%)	2.65 (0.65-10.87)	1
Kidney Cancer	Zero	5 / 5297 (0.09%)	-	
	One	60 / 26365 (0.23%)	2.41 (0.97-5.99)	0.414
	Two	128 / 56202 (0.23%)	2.47 (1.01-6.03)	0.334
	Three	96 / 57226 (0.17%)	1.86 (0.76-4.59)	1
	Four	42 / 27624 (0.15%)	1.71 (0.68-4.33)	1
Hypertension	Zero	835 / 5297 (15.76%)	-	
	One	3701 / 26365 (14.04%)	0.87 (0.81-0.94)	0.002
	Two	7203 / 56202 (12.82%)	0.8 (0.74-0.86)	<0.001
	Three	6740 / 57226 (11.78%)	0.74 (0.69-0.8)	<0.001
	Four	3214 / 27624 (11.63%)	0.73 (0.68-0.79)	<0.001
Ischemic Heart Disease	Zero	363 / 5297 (6.85%)	-	
	One	1642 / 26365 (6.23%)	0.92 (0.82-1.03)	1
	Two	3010 / 56202 (5.36%)	0.83 (0.75-0.93)	0.006
	Three	2650 / 57226 (4.63%)	0.76 (0.68-0.84)	<0.001
	Four	1178 / 27624 (4.26%)	0.72 (0.64-0.81)	<0.001
Pulmonary Embolism	Zero	33 / 5297 (0.62%)	-	
	One	161 / 26365 (0.61%)	0.98 (0.67-1.42)	1
	Two	366 / 56202 (0.65%)	1.06 (0.74-1.52)	1
	Three	314 / 57226 (0.55%)	0.91 (0.64-1.31)	1
	Four	166 / 27624 (0.6%)	1.01 (0.7-1.47)	1
Arrhythmias	Zero	203 / 5297 (3.83%)	-	
	One	979 / 26365 (3.71%)	0.97 (0.83-1.13)	1
	Two	1907 / 56202 (3.39%)	0.91 (0.79-1.06)	1
	Three	1718 / 57226 (3%)	0.84 (0.72-0.97)	0.12
	Four	747 / 27624 (2.7%)	0.77 (0.66-0.9)	0.006
Heart Failure	Zero	71 / 5297 (1.34%)	-	
	One	356 / 26365 (1.35%)	1.01 (0.79-1.31)	1
	Two	538 / 56202 (0.96%)	0.75 (0.59-0.96)	0.175
	Three	451 / 57226 (0.79%)	0.65 (0.51-0.84)	0.006
	Four	207 / 27624 (0.75%)	0.64 (0.49-0.84)	0.009
Cerebrovascular Diseases	Zero	123 / 5297 (2.32%)	-	
	One	486 / 26365 (1.84%)	0.79 (0.65-0.96)	0.127
	Two	822 / 56202 (1.46%)	0.64 (0.53-0.77)	<0.001
	Three	740 / 57226 (1.29%)	0.58 (0.48-0.7)	<0.001
	Four	335 / 27624 (1.21%)	0.55 (0.45-0.68)	<0.001
Arteriosclerosis	Zero	2 / 5297 (0.04%)	-	
	One	37 / 26365 (0.14%)	3.8 (0.91-15.75)	0.463
	Two	35 / 56202 (0.06%)	1.81 (0.43-7.51)	1
	Three	20 / 57226 (0.03%)	1.08 (0.25-4.64)	1
	Four	3 / 27624 (0.01%)	0.35 (0.06-2.13)	1
Deep Vein Thrombosis	Zero	33 / 5297 (0.62%)	-	
	One	142 / 26365 (0.54%)	0.87 (0.59-1.27)	1
	Two	298 / 56202 (0.53%)	0.87 (0.61-1.25)	1
	Three	279 / 57226 (0.49%)	0.82 (0.57-1.18)	1
	Four	156 / 27624 (0.56%)	0.96 (0.66-1.4)	1
Asthma	Zero	189 / 5297 (3.57%)	-	
	One	918 / 26365 (3.48%)	0.96 (0.82-1.12)	1
	Two	1921 / 56202 (3.42%)	0.92 (0.79-1.07)	1
	Three	1973 / 57226 (3.45%)	0.91 (0.79-1.06)	1
	Four	949 / 27624 (3.44%)	0.89 (0.77-1.05)	1
Liver Disease	Zero	74 / 5297 (1.4%)	-	
	One	311 / 26365 (1.18%)	0.84 (0.65-1.08)	1
	Two	498 / 56202 (0.89%)	0.64 (0.5-0.81)	0.002
	Three	455 / 57226 (0.8%)	0.57 (0.45-0.74)	<0.001
	Four	198 / 27624 (0.72%)	0.52 (0.4-0.68)	<0.001
Renal Failure	Zero	154 / 5297 (2.91%)	-	
	One	735 / 26365 (2.79%)	0.95 (0.8-1.13)	1
	Two	1292 / 56202 (2.3%)	0.8 (0.68-0.94)	0.059
	Three	1168 / 57226 (2.04%)	0.72 (0.61-0.86)	0.001
	Four	525 / 27624 (1.9%)	0.68 (0.57-0.81)	<0.001
Gout	Zero	53 / 5297 (1%)	-	
	One	189 / 26365 (0.72%)	0.76 (0.56-1.03)	0.536
	Two	278 / 56202 (0.49%)	0.58 (0.43-0.78)	0.002
	Three	203 / 57226 (0.35%)	0.47 (0.35-0.63)	<0.001
	Four	66 / 27624 (0.24%)	0.35 (0.24-0.5)	<0.001
Osteoarthritis	Zero	340 / 5297 (6.42%)	-	
	One	1743 / 26365 (6.61%)	0.99 (0.88-1.11)	1
	Two	3716 / 56202 (6.61%)	0.96 (0.86-1.08)	1
	Three	3724 / 57226 (6.51%)	0.93 (0.83-1.04)	1
	Four	1713 / 27624 (6.2%)	0.86 (0.76-0.96)	0.069
Sleep Disorders	Zero	37 / 5297 (0.7%)	-	
	One	179 / 26365 (0.68%)	1.01 (0.71-1.44)	1
	Two	323 / 56202 (0.57%)	0.89 (0.64-1.26)	1
	Three	271 / 57226 (0.47%)	0.77 (0.54-1.08)	0.929
	Four	122 / 27624 (0.44%)	0.74 (0.51-1.08)	0.822
Mood Disorders	Zero	152 / 5297 (2.87%)	-	
	One	686 / 26365 (2.6%)	0.88 (0.74-1.05)	1
	Two	1308 / 56202 (2.33%)	0.76 (0.64-0.9)	0.01
	Three	1188 / 57226 (2.08%)	0.66 (0.56-0.78)	<0.001
	Four	544 / 27624 (1.97%)	0.61 (0.51-0.73)	<0.001

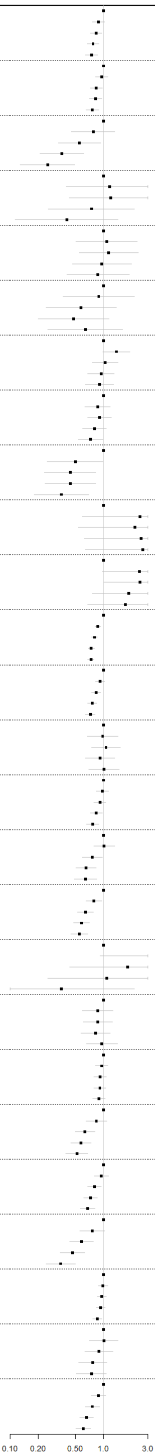

© 2023 Rassy N et al. *JAMA Network Open*.

C.

Disease	Healthy lifestyle factors	N (%) Cases	HR (95% CI)	Adjusted pvalue
Infections	Zero	154 / 3842 (4.01%)	-	
	One	779 / 16486 (4.73%)	1.16 (0.98-1.38)	0.588
	Two	1322 / 31114 (4.25%)	1.04 (0.88-1.23)	1
	Three	1274 / 29556 (4.31%)	1.05 (0.89-1.24)	1
	Four	494 / 13006 (3.8%)	0.92 (0.76-1.1)	1
Diabete	Zero	371 / 3842 (9.66%)	-	
	One	1543 / 16486 (9.36%)	0.99 (0.89-1.11)	1
	Two	2682 / 31114 (8.62%)	0.94 (0.85-1.05)	1
	Three	2464 / 29556 (8.34%)	0.95 (0.85-1.06)	1
	Four	973 / 13006 (7.48%)	0.88 (0.78-1)	0.33
Esophageal Adenocarcinoma	Zero	8 / 3842 (0.21%)	-	
	One	38 / 16486 (0.23%)	1.19 (0.56-2.55)	1
	Two	67 / 31114 (0.22%)	1.23 (0.59-2.56)	1
	Three	30 / 29556 (0.1%)	0.66 (0.3-1.44)	1
	Four	14 / 13006 (0.11%)	0.81 (0.34-1.95)	1
Gastric Cardia Cancer	Zero	4 / 3842 (0.1%)	-	
	One	11 / 16486 (0.07%)	0.68 (0.22-2.14)	1
	Two	38 / 31114 (0.12%)	1.37 (0.49-3.84)	1
	Three	20 / 29556 (0.07%)	0.86 (0.29-2.51)	1
	Four	10 / 13006 (0.08%)	1.12 (0.35-3.6)	1
Pancreatic Cancer	Zero	6 / 3842 (0.16%)	-	
	One	32 / 16486 (0.19%)	1.24 (0.52-2.97)	1
	Two	63 / 31114 (0.2%)	1.31 (0.57-3.04)	1
	Three	52 / 29556 (0.18%)	1.16 (0.5-2.72)	1
	Four	17 / 13006 (0.13%)	0.88 (0.34-2.24)	1
Liver Cancer	Zero	1 / 3842 (0.03%)	-	
	One	27 / 16486 (0.16%)	6.46 (0.88-47.55)	0.469
	Two	42 / 31114 (0.13%)	5.58 (0.77-40.61)	0.625
	Three	23 / 29556 (0.08%)	3.43 (0.46-25.47)	1
	Four	15 / 13006 (0.12%)	5.44 (0.72-41.4)	0.712
Colorectal Cancer	Zero	37 / 3842 (0.96%)	-	
	One	156 / 16486 (0.95%)	1 (0.7-1.43)	1
	Two	245 / 31114 (0.79%)	0.85 (0.6-1.21)	1
	Three	205 / 29556 (0.69%)	0.79 (0.55-1.12)	1
	Four	89 / 13006 (0.68%)	0.81 (0.55-1.2)	1
Breast Cancer	Zero	36 / 3842 (0.94%)	-	
	One	190 / 16486 (1.15%)	0.99 (0.69-1.41)	1
	Two	417 / 31114 (1.34%)	0.96 (0.68-1.35)	1
	Three	423 / 29556 (1.43%)	0.87 (0.62-1.22)	1
	Four	209 / 13006 (1.61%)	0.85 (0.6-1.21)	1
Ovarian Cancer	Zero	3 / 3842 (0.08%)	-	
	One	22 / 16486 (0.13%)	1.36 (0.41-4.53)	1
	Two	42 / 31114 (0.13%)	1.13 (0.35-3.64)	1
	Three	50 / 29556 (0.17%)	1.2 (0.37-3.84)	1
	Four	28 / 13006 (0.22%)	1.31 (0.4-4.33)	1
Endometrial Cancer	Zero	3 / 3842 (0.08%)	-	
	One	30 / 16486 (0.18%)	1.84 (0.56-6.04)	1
	Two	95 / 31114 (0.31%)	2.54 (0.8-8.02)	0.784
	Three	114 / 29556 (0.39%)	2.71 (0.86-8.52)	0.62
	Four	57 / 13006 (0.44%)	2.65 (0.83-8.45)	0.704
Kidney Cancer	Zero	8 / 3842 (0.21%)	-	
	One	51 / 16486 (0.31%)	1.53 (0.73-3.23)	1
	Two	82 / 31114 (0.26%)	1.37 (0.66-2.84)	1
	Three	64 / 29556 (0.22%)	1.21 (0.58-2.52)	1
	Four	24 / 13006 (0.18%)	1.1 (0.49-2.47)	1
Hypertension	Zero	827 / 3842 (21.53%)	-	
	One	3460 / 16486 (20.99%)	0.97 (0.9-1.05)	1
	Two	5971 / 31114 (19.19%)	0.89 (0.82-0.95)	0.008
	Three	5496 / 29556 (18.6%)	0.87 (0.81-0.94)	0.001
	Four	2253 / 13006 (17.32%)	0.81 (0.75-0.88)	<0.001
Ischemic Heart Disease	Zero	365 / 3842 (9.5%)	-	
	One	1442 / 16486 (8.75%)	0.96 (0.86-1.08)	1
	Two	2377 / 31114 (7.64%)	0.88 (0.79-0.99)	0.2
	Three	1947 / 29556 (6.59%)	0.82 (0.73-0.92)	0.003
	Four	701 / 13006 (5.39%)	0.72 (0.63-0.82)	<0.001
Pulmonary Embolism	Zero	35 / 3842 (0.91%)	-	
	One	183 / 16486 (1.11%)	1.21 (0.84-1.74)	1
	Two	288 / 31114 (0.93%)	1.01 (0.71-1.44)	1
	Three	257 / 29556 (0.87%)	0.96 (0.68-1.38)	1
	Four	108 / 13006 (0.83%)	0.93 (0.63-1.36)	1
Arrhythmias	Zero	220 / 3842 (5.73%)	-	
	One	914 / 16486 (5.54%)	0.99 (0.85-1.15)	1
	Two	1484 / 31114 (4.77%)	0.88 (0.77-1.02)	0.598
	Three	1244 / 29556 (4.21%)	0.82 (0.71-0.95)	0.057
	Four	440 / 13006 (3.38%)	0.7 (0.59-0.82)	<0.001
Heart Failure	Zero	88 / 3842 (2.29%)	-	
	One	399 / 16486 (2.42%)	1.1 (0.87-1.38)	1
	Two	617 / 31114 (1.98%)	0.94 (0.75-1.18)	1
	Three	422 / 29556 (1.43%)	0.73 (0.58-0.91)	0.045
	Four	173 / 13006 (1.33%)	0.73 (0.56-0.95)	0.127
Cerebrovascular Diseases	Zero	67 / 3842 (1.74%)	-	
	One	335 / 16486 (2.03%)	1.2 (0.92-1.56)	1
	Two	574 / 31114 (1.84%)	1.13 (0.87-1.45)	1
	Three	518 / 29556 (1.75%)	1.12 (0.87-1.45)	1
	Four	178 / 13006 (1.37%)	0.92 (0.7-1.23)	1
Arteriosclerosis	Zero	8 / 3842 (0.21%)	-	
	One	16 / 16486 (0.1%)	0.52 (0.22-1.21)	0.887
	Two	19 / 31114 (0.06%)	0.36 (0.16-0.83)	0.116
	Three	19 / 29556 (0.06%)	0.45 (0.19-1.02)	0.397
	Four	1 / 13006 (0.01%)	0.07 (0.01-0.52)	0.072
Deep Vein Thrombosis	Zero	40 / 3842 (1.04%)	-	
	One	144 / 16486 (0.87%)	0.83 (0.59-1.18)	1
	Two	239 / 31114 (0.77%)	0.73 (0.52-1.03)	0.502
	Three	221 / 29556 (0.75%)	0.72 (0.51-1.01)	0.423
	Four	97 / 13006 (0.75%)	0.73 (0.5-1.05)	0.645
Asthma	Zero	176 / 3842 (4.58%)	-	
	One	800 / 16486 (4.85%)	1.02 (0.87-1.2)	1
	Two	1512 / 31114 (4.86%)	0.99 (0.84-1.15)	1
	Three	1421 / 29556 (4.81%)	0.94 (0.8-1.1)	1
	Four	603 / 13006 (4.64%)	0.88 (0.74-1.04)	0.859
Liver Disease	Zero	74 / 3842 (1.93%)	-	
	One	324 / 16486 (1.97%)	1.02 (0.8-1.32)	1
	Two	557 / 31114 (1.79%)	0.94 (0.74-1.2)	1
	Three	446 / 29556 (1.51%)	0.8 (0.63-1.03)	0.55
	Four	176 / 13006 (1.35%)	0.72 (0.55-0.95)	0.149
Renal Failure	Zero	167 / 3842 (4.35%)	-	
	One	760 / 16486 (4.61%)	1.07 (0.91-1.27)	1
	Two	1283 / 31114 (4.12%)	0.97 (0.82-1.14)	1
	Three	1057 / 29556 (3.58%)	0.86 (0.73-1.01)	0.497
	Four	407 / 13006 (3.13%)	0.77 (0.64-0.92)	0.035
Gout	Zero	66 / 3842 (1.72%)	-	
	One	238 / 16486 (1.44%)	0.91 (0.69-1.19)	1
	Two	316 / 31114 (1.02%)	0.71 (0.54-0.92)	0.071
	Three	204 / 29556 (0.69%)	0.55 (0.41-0.72)	<0.001
	Four	65 / 13006 (0.5%)	0.47 (0.33-0.66)	<0.001
Osteoarthritis	Zero	411 / 3842 (10.7%)	-	
	One	1824 / 16486 (11.06%)	0.99 (0.89-1.1)	1
	Two	3475 / 31114 (11.17%)	0.97 (0.87-1.07)	1
	Three	3225 / 29556 (10.91%)	0.92 (0.83-1.02)	0.661
	Four	1424 / 13006 (10.95%)	0.89 (0.8-0.99)	0.276
Sleep Disorders	Zero	102 / 3842 (2.65%)	-	
	One	394 / 16486 (2.39%)	0.94 (0.76-1.17)	1
	Two	604 / 31114 (1.94%)	0.81 (0.66-1)	0.348
	Three	462 / 29556 (1.56%)	0.69 (0.56-0.86)	0.005
	Four	185 / 13006 (1.42%)	0.67 (0.53-0.86)	0.009
Mood Disorders	Zero	163 / 3842 (4.24%)	-	
	One	643 / 16486 (3.9%)	0.88 (0.74-1.04)	0.958
	Two	1125 / 31114 (3.62%)	0.78 (0.66-0.92)	0.02
	Three	1063 / 29556 (3.6%)	0.74 (0.63-0.87)	0.002
	Four	403 / 13006 (3.1%)	0.61 (0.5-0.73)	<0.001

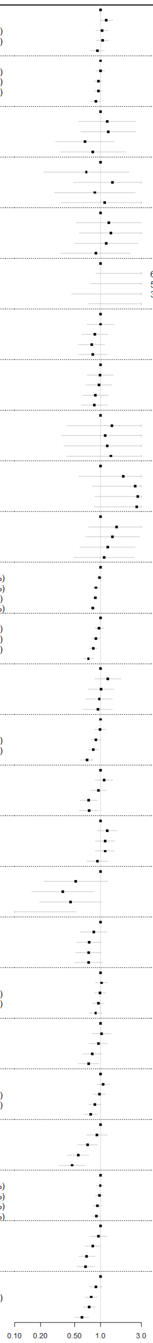

eFigure 10. Adjusted Hazard Ratios for Incident Diseases by Lifestyle Profile in Individuals With Normal Weight (BMI 18.5-24.9) (A), Overweight (BMI 25-29.9) (B), and Obesity (BMI ≥30) (C) After Exclusion of Participants With Diseases Diagnosed Within the First 2 Years of Follow-up

BMI, body mass index; HR, hazard ratio; CI, confidence interval

Hazard ratios (95% CI) for a lifestyle score equal to 1, 2, 3 or 4 versus a lifestyle score equal to zero were calculated in Cox proportional hazards models. All the p-values are presented after Bonferroni multiple testing adjustments.

For each lifestyle factor, individuals scored 1 if they met the criterion for a healthy lifestyle (never smoking, no or moderate alcohol consumption, physical activity, or healthy diet) and 0 otherwise. The healthy lifestyle score was computed by summing the four scores. It ranged between zero (lowest healthy score) and 4 (highest healthy score).

© 2023 Rassy N et al. *JAMA Network Open*.

eFigure 11. Association of Healthy Lifestyle Score With Incident Diseases in Adults With a Healthy Weight

Disease	Healthy lifestyle factors	N (%) Cases	HR (95% CI)	Corrected pvalue
Infections	Zero	169 / 3190 (5.3%)	–	
	One	794 / 17689 (4.49%)	0.84 (0.71–1)	0.31
	Two	1617 / 44001 (3.67%)	0.69 (0.59–0.81)	< 0.001
	Three	1652 / 51199 (3.23%)	0.61 (0.52–0.72)	< 0.001
	Four	819 / 28500 (2.87%)	0.54 (0.46–0.64)	< 0.001
Diabete	Zero	67 / 3190 (2.1%)	–	
	One	281 / 17689 (1.59%)	0.81 (0.62–1.06)	0.85
	Two	633 / 44001 (1.44%)	0.8 (0.62–1.03)	0.61
	Three	732 / 51199 (1.43%)	0.82 (0.64–1.06)	0.87
	Four	348 / 28500 (1.22%)	0.7 (0.54–0.91)	0.06
Esophageal Adenocarcinoma	Zero	11 / 3190 (0.34%)	–	
	One	33 / 17689 (0.19%)	0.57 (0.29–1.13)	0.75
	Two	61 / 44001 (0.14%)	0.46 (0.24–0.87)	0.13
	Three	41 / 51199 (0.08%)	0.27 (0.14–0.53)	< 0.001
	Four	18 / 28500 (0.06%)	0.21 (0.1–0.45)	< 0.001
Gastric Cardia Cancer	Zero	5 / 3190 (0.16%)	–	
	One	11 / 17689 (0.06%)	0.42 (0.15–1.22)	0.78
	Two	18 / 44001 (0.04%)	0.31 (0.11–0.84)	0.15
	Three	19 / 51199 (0.04%)	0.28 (0.11–0.77)	0.09
	Four	7 / 28500 (0.02%)	0.19 (0.06–0.6)	0.03
Pancreatic Cancer	Zero	6 / 3190 (0.19%)	–	
	One	40 / 17689 (0.23%)	1.21 (0.51–2.85)	1
	Two	71 / 44001 (0.16%)	0.89 (0.38–2.04)	1
	Three	63 / 51199 (0.12%)	0.68 (0.29–1.57)	1
	Four	39 / 28500 (0.14%)	0.74 (0.31–1.75)	1
Liver Cancer	Zero	6 / 3190 (0.19%)	–	
	One	21 / 17689 (0.12%)	0.63 (0.25–1.57)	1
	Two	34 / 44001 (0.08%)	0.42 (0.17–1)	0.35
	Three	40 / 51199 (0.08%)	0.42 (0.18–1.01)	0.36
	Four	13 / 28500 (0.05%)	0.24 (0.09–0.65)	0.03
Colorectal Cancer	Zero	22 / 3190 (0.69%)	–	
	One	148 / 17689 (0.84%)	1.2 (0.77–1.88)	1
	Two	320 / 44001 (0.73%)	1.05 (0.68–1.63)	1
	Three	335 / 51199 (0.65%)	0.94 (0.61–1.45)	1
	Four	183 / 28500 (0.64%)	0.91 (0.58–1.41)	1
Breast Cancer	Zero	43 / 3190 (1.35%)	–	
	One	302 / 17689 (1.71%)	1.09 (0.8–1.51)	1
	Two	771 / 44001 (1.75%)	0.99 (0.73–1.35)	1
	Three	871 / 51199 (1.7%)	0.94 (0.69–1.28)	1
	Four	449 / 28500 (1.58%)	0.85 (0.62–1.17)	1
Ovarian Cancer	Zero	2 / 3190 (0.06%)	–	
	One	25 / 17689 (0.14%)	1.92 (0.46–8.11)	1
	Two	76 / 44001 (0.17%)	2.05 (0.5–8.35)	1
	Three	99 / 51199 (0.19%)	2.25 (0.55–9.12)	1
	Four	63 / 28500 (0.22%)	2.49 (0.61–10.17)	1
Endometrial Cancer	Zero	0 / 3190 (0%)	–	
	One	23 / 17689 (0.13%)	46643858.31 (0–Inf)	1
	Two	52 / 44001 (0.12%)	36721818.75 (0–Inf)	1
	Three	88 / 51199 (0.17%)	51875604.79 (0–Inf)	1
	Four	53 / 28500 (0.19%)	54142208.55 (0–Inf)	1
Kidney Cancer	Zero	8 / 3190 (0.25%)	–	
	One	27 / 17689 (0.15%)	0.62 (0.28–1.36)	1
	Two	49 / 44001 (0.11%)	0.47 (0.22–1)	0.34
	Three	66 / 51199 (0.13%)	0.54 (0.26–1.13)	0.73
	Four	28 / 28500 (0.1%)	0.41 (0.18–0.89)	0.18
Hypertension	Zero	436 / 3190 (13.67%)	–	
	One	1961 / 17689 (11.09%)	0.8 (0.72–0.88)	< 0.001
	Two	3966 / 44001 (9.01%)	0.65 (0.59–0.72)	< 0.001
	Three	4134 / 51199 (8.07%)	0.58 (0.53–0.64)	< 0.001
	Four	2281 / 28500 (8%)	0.56 (0.51–0.63)	< 0.001
Ischemic Heart Disease	Zero	219 / 3190 (6.87%)	–	
	One	930 / 17689 (5.26%)	0.8 (0.69–0.93)	0.02
	Two	1720 / 44001 (3.91%)	0.64 (0.55–0.74)	< 0.001
	Three	1744 / 51199 (3.41%)	0.56 (0.49–0.65)	< 0.001
	Four	908 / 28500 (3.19%)	0.52 (0.45–0.6)	< 0.001
Pulmonary Embolism	Zero	31 / 3190 (0.97%)	–	
	One	116 / 17689 (0.66%)	0.69 (0.46–1.02)	0.46
	Two	193 / 44001 (0.44%)	0.48 (0.33–0.7)	0.001
	Three	204 / 51199 (0.4%)	0.44 (0.3–0.64)	< 0.001
	Four	110 / 28500 (0.39%)	0.42 (0.28–0.63)	< 0.001
Arrhythmias	Zero	134 / 3190 (4.2%)	–	
	One	619 / 17689 (3.5%)	0.85 (0.71–1.03)	0.65
	Two	1317 / 44001 (2.99%)	0.76 (0.64–0.91)	0.02
	Three	1387 / 51199 (2.71%)	0.69 (0.58–0.83)	< 0.001
	Four	800 / 28500 (2.81%)	0.71 (0.59–0.85)	0.002
Heart Failure	Zero	52 / 3190 (1.63%)	–	
	One	209 / 17689 (1.18%)	0.76 (0.56–1.03)	0.53
	Two	364 / 44001 (0.83%)	0.57 (0.43–0.77)	0.001
	Three	334 / 51199 (0.65%)	0.46 (0.34–0.62)	< 0.001
	Four	177 / 28500 (0.62%)	0.43 (0.32–0.59)	< 0.001
Cerebrovascular Diseases	Zero	85 / 3190 (2.66%)	–	
	One	349 / 17689 (1.97%)	0.76 (0.6–0.97)	0.19
	Two	650 / 44001 (1.48%)	0.61 (0.48–0.76)	< 0.001
	Three	597 / 51199 (1.17%)	0.49 (0.39–0.61)	< 0.001
	Four	356 / 28500 (1.25%)	0.52 (0.41–0.65)	< 0.001
Arteriosclerosis	Zero	12 / 3190 (0.38%)	–	
	One	33 / 17689 (0.19%)	0.56 (0.29–1.08)	0.58
	Two	36 / 44001 (0.08%)	0.28 (0.15–0.55)	0.001
	Three	19 / 51199 (0.04%)	0.13 (0.06–0.28)	< 0.001
	Four	10 / 28500 (0.04%)	0.13 (0.06–0.3)	< 0.001
Deep Vein Thrombosis	Zero	30 / 3190 (0.94%)	–	
	One	114 / 17689 (0.64%)	0.71 (0.47–1.06)	0.63
	Two	227 / 44001 (0.52%)	0.59 (0.4–0.86)	0.05
	Three	217 / 51199 (0.42%)	0.49 (0.33–0.72)	0.002
	Four	143 / 28500 (0.5%)	0.58 (0.39–0.86)	0.04
Asthma	Zero	156 / 3190 (4.89%)	–	
	One	682 / 17689 (3.86%)	0.78 (0.65–0.93)	0.03
	Two	1540 / 44001 (3.5%)	0.7 (0.6–0.83)	< 0.001
	Three	1745 / 51199 (3.41%)	0.69 (0.58–0.81)	< 0.001
	Four	959 / 28500 (3.36%)	0.67 (0.57–0.8)	< 0.001
Liver Disease	Zero	53 / 3190 (1.66%)	–	
	One	230 / 17689 (1.3%)	0.83 (0.61–1.12)	1
	Two	361 / 44001 (0.82%)	0.56 (0.42–0.74)	< 0.001
	Three	307 / 51199 (0.6%)	0.42 (0.31–0.56)	< 0.001
	Four	143 / 28500 (0.5%)	0.35 (0.26–0.48)	< 0.001
Renal Failure	Zero	93 / 3190 (2.92%)	–	
	One	436 / 17689 (2.46%)	0.87 (0.69–1.09)	1
	Two	794 / 44001 (1.8%)	0.67 (0.54–0.83)	0.002
	Three	740 / 51199 (1.45%)	0.54 (0.44–0.68)	< 0.001
	Four	422 / 28500 (1.48%)	0.55 (0.44–0.69)	< 0.001
Gout	Zero	13 / 3190 (0.41%)	–	
	One	41 / 17689 (0.23%)	0.63 (0.34–1.18)	1
	Two	72 / 44001 (0.16%)	0.52 (0.29–0.94)	0.22
	Three	61 / 51199 (0.12%)	0.39 (0.21–0.71)	0.01
	Four	26 / 28500 (0.09%)	0.3 (0.15–0.59)	0.003
Osteoarthritis	Zero	149 / 3190 (4.67%)	–	
	One	964 / 17689 (5.45%)	1.11 (0.93–1.32)	1
	Two	2224 / 44001 (5.05%)	0.99 (0.84–1.17)	1
	Three	2480 / 51199 (4.84%)	0.94 (0.8–1.11)	1
	Four	1295 / 28500 (4.54%)	0.85 (0.72–1.01)	0.50
Sleep Disorders	Zero	16 / 3190 (0.5%)	–	
	One	81 / 17689 (0.46%)	0.99 (0.58–1.69)	1
	Two	139 / 44001 (0.32%)	0.74 (0.44–1.24)	1
	Three	160 / 51199 (0.31%)	0.75 (0.45–1.26)	1
	Four	83 / 28500 (0.29%)	0.71 (0.41–1.21)	1
Mood Disorders	Zero	131 / 3190 (4.11%)	–	
	One	592 / 17689 (3.35%)	0.81 (0.67–0.98)	0.21
	Two	1121 / 44001 (2.55%)	0.62 (0.52–0.74)	< 0.001
	Three	1085 / 51199 (2.12%)	0.52 (0.43–0.62)	< 0.001
	Four	484 / 28500 (1.7%)	0.42 (0.34–0.51)	< 0.001

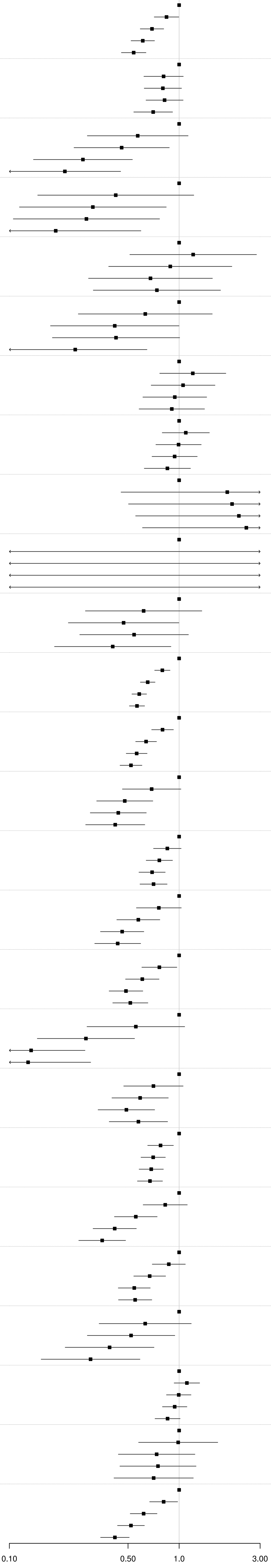

© 2023 Rassy N et al. *JAMA Network Open.*

eFigure 12. Association of Healthy Lifestyle Score With Incident Diseases in Adults With Obesity

Disease	Healthy lifestyle factors	N (%) Cases	HR (95% CI)	Corrected pvalue
Infections	Zero	221 / 4381 (5.04%)	–	
	One	1079 / 19054 (5.66%)	1.12 (0.97–1.29)	0.97
	Two	1899 / 35517 (5.35%)	1.05 (0.91–1.2)	1
	Three	1707 / 33484 (5.1%)	0.99 (0.86–1.14)	1
	Four	663 / 14605 (4.54%)	0.88 (0.75–1.02)	0.66
Diabete	Zero	522 / 4381 (11.92%)	–	
	One	2218 / 19054 (11.64%)	1 (0.91–1.1)	1
	Two	3884 / 35517 (10.94%)	0.97 (0.88–1.06)	1
	Three	3518 / 33484 (10.51%)	0.97 (0.88–1.06)	1
	Four	1358 / 14605 (9.3%)	0.88 (0.8–0.98)	0.11
Esophageal Adenocarcinoma	Zero	11 / 4381 (0.25%)	–	
	One	61 / 19054 (0.32%)	1.37 (0.72–2.61)	1
	Two	96 / 35517 (0.27%)	1.28 (0.69–2.39)	1
	Three	48 / 33484 (0.14%)	0.78 (0.4–1.5)	1
	Four	17 / 14605 (0.12%)	0.74 (0.34–1.58)	1
Gastric Cardia Cancer	Zero	4 / 4381 (0.09%)	–	
	One	23 / 19054 (0.12%)	1.41 (0.49–4.07)	1
	Two	47 / 35517 (0.13%)	1.7 (0.61–4.72)	1
	Three	30 / 33484 (0.09%)	1.32 (0.46–3.75)	1
	Four	11 / 14605 (0.08%)	1.29 (0.41–4.06)	1
Pancreatic Cancer	Zero	8 / 4381 (0.18%)	–	
	One	41 / 19054 (0.22%)	1.17 (0.55–2.5)	1
	Two	84 / 35517 (0.24%)	1.29 (0.63–2.67)	1
	Three	75 / 33484 (0.22%)	1.24 (0.6–2.58)	1
	Four	18 / 14605 (0.12%)	0.69 (0.3–1.59)	1
Liver Cancer	Zero	4 / 4381 (0.09%)	–	
	One	37 / 19054 (0.19%)	2.21 (0.79–6.2)	0.92
	Two	71 / 35517 (0.2%)	2.41 (0.88–6.6)	0.61
	Three	36 / 33484 (0.11%)	1.4 (0.5–3.94)	1
	Four	17 / 14605 (0.12%)	1.63 (0.55–4.89)	1
Colorectal Cancer	Zero	54 / 4381 (1.23%)	–	
	One	226 / 19054 (1.19%)	0.98 (0.73–1.32)	1
	Two	346 / 35517 (0.97%)	0.84 (0.63–1.12)	1
	Three	278 / 33484 (0.83%)	0.75 (0.56–1.01)	0.41
	Four	123 / 14605 (0.84%)	0.8 (0.58–1.11)	1
Breast Cancer	Zero	51 / 4381 (1.16%)	–	
	One	264 / 19054 (1.39%)	0.96 (0.71–1.3)	1
	Two	582 / 35517 (1.64%)	0.94 (0.7–1.25)	1
	Three	573 / 33484 (1.71%)	0.83 (0.62–1.1)	1
	Four	287 / 14605 (1.97%)	0.82 (0.61–1.1)	1
Ovarian Cancer	Zero	4 / 4381 (0.09%)	–	
	One	29 / 19054 (0.15%)	1.33 (0.47–3.79)	1
	Two	56 / 35517 (0.16%)	1.12 (0.41–3.09)	1
	Three	67 / 33484 (0.2%)	1.19 (0.44–3.27)	1
	Four	39 / 14605 (0.27%)	1.37 (0.49–3.82)	1
Endometrial Cancer	Zero	6 / 4381 (0.14%)	–	
	One	43 / 19054 (0.23%)	1.31 (0.56–3.08)	1
	Two	137 / 35517 (0.39%)	1.81 (0.8–4.1)	1
	Three	164 / 33484 (0.49%)	1.93 (0.85–4.36)	0.80
	Four	76 / 14605 (0.52%)	1.75 (0.76–4.01)	1
Kidney Cancer	Zero	11 / 4381 (0.25%)	–	
	One	71 / 19054 (0.37%)	1.52 (0.8–2.87)	1
	Two	127 / 35517 (0.36%)	1.52 (0.82–2.81)	1
	Three	88 / 33484 (0.26%)	1.18 (0.63–2.21)	1
	Four	32 / 14605 (0.22%)	1.03 (0.52–2.06)	1
Hypertension	Zero	1120 / 4381 (25.56%)	–	
	One	4843 / 19054 (25.42%)	1 (0.94–1.07)	1
	Two	8270 / 35517 (23.28%)	0.91 (0.85–0.97)	0.02
	Three	7517 / 33484 (22.45%)	0.89 (0.83–0.94)	0.001
	Four	3081 / 14605 (21.1%)	0.84 (0.78–0.9)	< 0.001
Ischemic Heart Disease	Zero	544 / 4381 (12.42%)	–	
	One	2261 / 19054 (11.87%)	1 (0.91–1.1)	1
	Two	3709 / 35517 (10.44%)	0.92 (0.84–1.01)	0.62
	Three	2944 / 33484 (8.79%)	0.83 (0.76–0.91)	< 0.001
	Four	1031 / 14605 (7.06%)	0.72 (0.65–0.8)	< 0.001
Pulmonary Embolism	Zero	49 / 4381 (1.12%)	–	
	One	249 / 19054 (1.31%)	1.16 (0.86–1.58)	1
	Two	414 / 35517 (1.17%)	1.04 (0.77–1.4)	1
	Three	355 / 33484 (1.06%)	0.96 (0.71–1.29)	1
	Four	155 / 14605 (1.06%)	0.97 (0.7–1.34)	1
Arrhythmias	Zero	306 / 4381 (6.98%)	–	
	One	1355 / 19054 (7.11%)	1.05 (0.93–1.19)	1
	Two	2180 / 35517 (6.14%)	0.94 (0.83–1.05)	1
	Three	1809 / 33484 (5.4%)	0.87 (0.77–0.98)	0.19
	Four	609 / 14605 (4.17%)	0.71 (0.61–0.81)	< 0.001
Heart Failure	Zero	140 / 4381 (3.2%)	–	
	One	620 / 19054 (3.25%)	1.06 (0.88–1.27)	1
	Two	991 / 35517 (2.79%)	0.95 (0.8–1.13)	1
	Three	701 / 33484 (2.09%)	0.77 (0.64–0.92)	0.03
	Four	241 / 14605 (1.65%)	0.65 (0.53–0.8)	< 0.001
Cerebrovascular Diseases	Zero	114 / 4381 (2.6%)	–	
	One	537 / 19054 (2.82%)	1.11 (0.91–1.36)	1
	Two	877 / 35517 (2.47%)	1 (0.82–1.22)	1
	Three	756 / 33484 (2.26%)	0.96 (0.79–1.17)	1
	Four	248 / 14605 (1.7%)	0.75 (0.6–0.94)	0.09
Arteriosclerosis	Zero	11 / 4381 (0.25%)	–	
	One	42 / 19054 (0.22%)	0.93 (0.48–1.81)	1
	Two	44 / 35517 (0.12%)	0.56 (0.29–1.09)	0.63
	Three	46 / 33484 (0.14%)	0.69 (0.36–1.34)	1
	Four	5 / 14605 (0.03%)	0.19 (0.07–0.56)	0.02
Deep Vein Thrombosis	Zero	59 / 4381 (1.35%)	–	
	One	219 / 19054 (1.15%)	0.85 (0.64–1.14)	1
	Two	385 / 35517 (1.08%)	0.81 (0.62–1.07)	0.96
	Three	332 / 33484 (0.99%)	0.75 (0.57–1)	0.33
	Four	135 / 14605 (0.92%)	0.71 (0.52–0.97)	0.21
Asthma	Zero	243 / 4381 (5.55%)	–	
	One	1167 / 19054 (6.12%)	1.07 (0.93–1.23)	1
	Two	2160 / 35517 (6.08%)	1.02 (0.9–1.17)	1
	Three	1998 / 33484 (5.97%)	0.97 (0.85–1.11)	1
	Four	850 / 14605 (5.82%)	0.91 (0.79–1.05)	1
Liver Disease	Zero	107 / 4381 (2.44%)	–	
	One	496 / 19054 (2.6%)	1.08 (0.87–1.33)	1
	Two	792 / 35517 (2.23%)	0.93 (0.76–1.14)	1
	Three	657 / 33484 (1.96%)	0.83 (0.68–1.02)	0.52
	Four	253 / 14605 (1.73%)	0.74 (0.59–0.93)	0.07
Renal Failure	Zero	250 / 4381 (5.71%)	–	
	One	1132 / 19054 (5.94%)	1.06 (0.92–1.21)	1
	Two	1901 / 35517 (5.35%)	0.96 (0.84–1.1)	1
	Three	1546 / 33484 (4.62%)	0.85 (0.75–0.98)	0.15
	Four	566 / 14605 (3.88%)	0.73 (0.63–0.85)	< 0.001
Gout	Zero	85 / 4381 (1.94%)	–	
	One	325 / 19054 (1.71%)	0.95 (0.74–1.2)	1
	Two	458 / 35517 (1.29%)	0.79 (0.63–1)	0.34
	Three	287 / 33484 (0.86%)	0.6 (0.47–0.77)	< 0.001
	Four	91 / 14605 (0.62%)	0.51 (0.38–0.69)	< 0.001
Osteoarthritis	Zero	583 / 4381 (13.31%)	–	
	One	2587 / 19054 (13.58%)	0.98 (0.9–1.08)	1
	Two	4944 / 35517 (13.92%)	0.97 (0.89–1.06)	1
	Three	4612 / 33484 (13.77%)	0.94 (0.86–1.02)	0.97
	Four	1974 / 14605 (13.52%)	0.88 (0.81–0.97)	0.07
Sleep Disorders	Zero	152 / 4381 (3.47%)	–	
	One	603 / 19054 (3.16%)	0.96 (0.8–1.15)	1
	Two	937 / 35517 (2.64%)	0.85 (0.71–1.01)	0.41
	Three	732 / 33484 (2.19%)	0.74 (0.62–0.89)	0.006
	Four	274 / 14605 (1.88%)	0.68 (0.56–0.83)	0.001
Mood Disorders	Zero	214 / 4381 (4.88%)	–	
	One	935 / 19054 (4.91%)	0.97 (0.84–1.13)	1
	Two	1642 / 35517 (4.62%)	0.88 (0.76–1.01)	0.48
	Three	1495 / 33484 (4.46%)	0.81 (0.7–0.93)	0.03
	Four	560 / 14605 (3.83%)	0.66 (0.56–0.78)	< 0.001

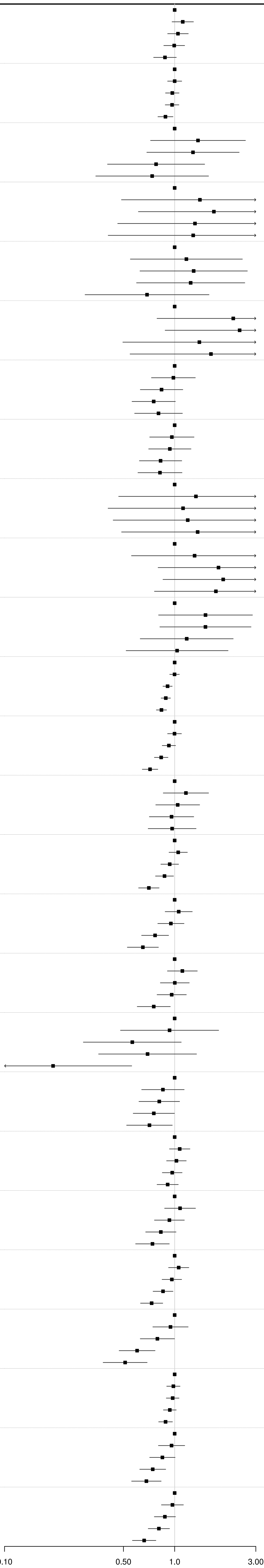

© 2023 Rassy N et al. *JAMA Network Open.*
