# Supplementary material for: Association of Healthy Lifestyle Factors and Obesity-Related Diseases in Adults in the UK
Source: JAMA Netw Open. 2023 May 26;6(5):e2314741. doi: 10.1001/jamanetworkopen.2023.14741 (PMC10220514; doi:10.1001/jamanetworkopen.2023.14741)
Supplement: Supplement 2. — Data Sharing Statement [file jamanetwopen-e2314741-s002.pdf]

Data Sharing Statement

Rassy. Association of Healthy Lifestyle Factors and Obesity-Related Diseases in Adults in the UK. *JAMA Netw Open*. Published May 26, 2023. doi:10.1001/jamanetworkopen.2023.14741

Data
Data available: Yes
Data types: Deidentified participant data, Other (please specify)
Additional Information: Data from UK Biobank
How to access data: Data from UK Biobank are available on application at www.ukbiobank.ac.uk/register-apply
When available: With publication

Supporting Documents
Document types: None

Additional Information
Who can access the data: anyone requesting the data
Types of analyses: for any purpose or for a specified purpose
Mechanisms of data availability: NA
